# Identification of potential and novel target genes in pituitary prolactinoma by bioinformatics analysis

**DOI:** 10.3934/Neuroscience.2021014

**Published:** 2021-02-07

**Authors:** Vikrant Ghatnatti, Basavaraj Vastrad, Swetha Patil, Chanabasayya Vastrad, Iranna Kotturshetti

**Affiliations:** 1 Department of Endocrinology, J N Medical College, Belagavi and KLE Academy of Higher Education & Research 590010, Karnataka, India; 2 Department of Biochemistry, Basaveshwar College of Pharmacy, Gadag, Karnataka 582103, India; 3 Department of Obstetrics and Gynaecology, J N Medical College, Belagavi and KLE Academy of Higher Education & Research 590010, Karnataka, India; 4 Biostatistics and Bioinformatics, Chanabasava Nilaya, Bharthinagar, Dharwad 580001, Karanataka, India; 5 Department of Ayurveda, Rajiv Gandhi Education Society's Ayurvedic Medical College, Ron 562209, Karanataka, India

**Keywords:** pituitary prolactinoma, differentially expressed genes, gene ontology, pathway enrichment analysis, protein-protein interactions

## Abstract

Pituitary prolactinoma is one of the most complicated and fatally pathogenic pituitary adenomas. Therefore, there is an urgent need to improve our understanding of the underlying molecular mechanism that drives the initiation, progression, and metastasis of pituitary prolactinoma. The aim of the present study was to identify the key genes and signaling pathways associated with pituitary prolactinoma using bioinformatics analysis. Transcriptome microarray dataset GSE119063 was downloaded from Gene Expression Omnibus (GEO) database. Limma package in R software was used to screen DEGs. Pathway and Gene ontology (GO) enrichment analysis were conducted to identify the biological role of DEGs. A protein-protein interaction (PPI) network was constructed and analyzed by using HIPPIE database and Cytoscape software. Module analyses was performed. In addition, a target gene-miRNA regulatory network and target gene-TF regulatory network were constructed by using NetworkAnalyst and Cytoscape software. Finally, validation of hub genes by receiver operating characteristic (ROC) curve analysis. A total of 989 DEGs were identified, including 461 up regulated genes and 528 down regulated genes. Pathway enrichment analysis showed that the DEGs were significantly enriched in the retinoate biosynthesis II, signaling pathways regulating pluripotency of stem cells, ALK2 signaling events, vitamin D3 biosynthesis, cell cycle and aurora B signaling. Gene Ontology (GO) enrichment analysis showed that the DEGs were significantly enriched in the sensory organ morphogenesis, extracellular matrix, hormone activity, nuclear division, condensed chromosome and microtubule binding. In the PPI network and modules, SOX2, PRSS45, CLTC, PLK1, B4GALT6, RUNX1 and GTSE1 were considered as hub genes. In the target gene-miRNA regulatory network and target gene-TF regulatory network, LINC00598, SOX4, IRX1 and UNC13A were considered as hub genes. Using integrated bioinformatics analysis, we identified candidate genes in pituitary prolactinoma, which might improve our understanding of the molecular mechanisms of pituitary prolactinoma.

## 1. Introduction

Prolactinoma is named as prolactin secreting pituitary adenoma seen more frequently in women and is characterized by irregular menstrual, erectile dysfunction, eye problems and loss of sexual function [1]. Signs and symptoms include amenorrhoea, glactorrhoea, headache, anaemia and hypertension [2]. Prolactinoma involves 20.7% among other pituitary adenomas [3]. Although treatment methods, including surgery [4], chemotherapy [5] and radiotherapy [6] are improving, overall survival rate remains lower. Consequently, elucidating the molecular mechanism associated in the pituitary prolactinoma is essential for the improvement of efficacious diagnosis and treatment strategies.

Presently, a wealth of previous studies has been enforced to advance a better understanding of the molecular mechanisms of pituitary prolactinoma. One study showed that allelic loss of DRD2 was responsible for development of pituitary prolactinoma [7]. Elevated expression of HMGA1 and HMGA2 were responsible for progression of pituitary prolactinoma [8],[9]. BMP4 was associated with progression of pituitary prolactinoma [10]. FGF4 was responsible for improvement of pituitary prolactinoma through invasion and cell proliferation [11]. Alteration in oncogene GNAS was important for progression of pituitary prolactinoma [12]. Stimulation of Raf/MEK/ERK and PI3K/Akt/mTOR signaling pathways were linked with development of prolactinoma [13]. Abnormal expression of CTNNB1 was involved in progression of prolactinoma [14]. Epigenetic inactivation of CDKN2A was responsible for advancement of prolactinoma [15]. AIP play key role in pathogenesis of pituitary prolactinoma [16]. Hence, searching for specific and sensitive molecular marker as well as some core genes or proteins will benefit the diagnosis and treatment of pituitary prolactinoma.

At current study, microarray analyses have been applied for medical research [17]. In this analysis, we chose GSE119063 dataset from Gene Expression Omnibus (GEO) (http://www.ncbi.nlm.nih.gov/geo/) and used limma bioconductor package to find the differentially expressed genes (DEGs). Subsequently, we made pathway enrichment and gene ontology (biological process (BP), molecular function (MF), cellular component (CC) analyses were performed. In addition, we constructed and analyzed PPI network of the DEGs and selected core genes with a high degree of connectivity, high betweenness centrality, high stress centrality, high closeness centrality and low clustering coefficient, and modules analysis were performed. Moreover, miRNA-target gene regulatory network and TF-target gene regulatory network were constructed and analyzed. Finally, hub genes are validated by receiver operating characteristic (ROC) curve analysis. Briefly, this study would provide novel targets for diagnosis, prognosis and treatment of pituitary prolactinoma.

## 2. Materials

### 2.1. Microarray data

We chose a gene expression profile of GSE119063 from GEO database. GSE119063 was based on the Agilent GPL13607 platform (Agilent-028004 SurePrint G3 Human GE 8x60K Microarray (Feature Number version). The GSE119063 dataset included 9 samples, containing 5 pituitary prolactinoma samples and 4 normal pituitaries samples. Besides, we downloaded the Series Matrix File of GSE119063 from GEO database.

### 2.2. Data preprocessing

The raw data used in this study were downloaded. The raw GSE119063 data was preprocessed by the Limma package (http://www.bioconductor.org/packages/release/bioc/html/limma.html) [18] in Bioconductor. The data preprocessing included background correction and quantile normalization. Probe identities (IDs) are mapped to gene IDs using the corresponding platform files.

### 2.3. Identification of DEGs

The Limma package was subsequently used for identifying DEGs. P < 0.05 and absolute fold change ≥0.93 for up regulated gene and fold change ≥−0.29 for down regulated gene [19] were considered as the cutoff values for DEG screening using the Benjamini & Hochberg procedure. R software was used to produce heat maps of common significant differentially expressed genes between pituitary prolactinoma samples and normal pituitaries samples. Genes are ordered according to the fold change in the expression values. This information was presented as a heat map and a volcano plot.

### 2.4. Pathway enrichment analysis of DEGs

BIOCYC (https://biocyc.org/) [20], Kyoto Encyclopedia of Genes and Genomes (KEGG) (http://www.genome.jp/kegg/pathway.html) [21], Pathway Interaction Database (PID) (https://wiki.nci.nih.gov/pages/viewpage.action?pageId=315491760) [22], REACTOME (https://reactome.org/) [23], GenMAPP (http://www.genmapp.org/) [24], MSigDB C2 BIOCARTA (http://software.broadinstitute.org/gsea/msigdb/collections.jsp) [25], PantherDB (http://www.pantherdb.org/) [26], Pathway Ontology (http://www.obofoundry.org/ontology/pw.html) [27] and Small Molecule Pathway Database (SMPDB) (http://smpdb.ca/) [28] were a collection of databases which helps to handle genomes, biological pathways, diseases, chemical substances, and drugs. ToppGene (https://toppgene.cchmc.org/enrichment.jsp) is a web-based online bioinformatics resource that aims to provide tools for the functional interpretation of large lists of genes or proteins [29]. P value < 0.05 is regarded as the cutoff criterion. We could visualize the pathways among those DEGs using ToppGene.

### 2.5. Gene Ontology (GO) enrichment analysis of DEGs

Gene ontology (GO) (http://www.geneontology.org/) enrichment analysis served as a useful approach to annotate genes and gene products and also analyze characteristic biological attributing to high-throughput genome or transcriptome data [30]. ToppGene (https://toppgene.cchmc.org/enrichment.jsp) is a web-based online bioinformatics resource that aims to provide tools for the functional interpretation of large lists of genes or proteins [29]. P value < 0.05 is regarded as the cutoff criterion. We could visualize the core biological process (BP), molecular function (MF) and cellular component (CC) among those DEGs using ToppGene.

### 2.6. PPI network construction and topology analysis

The Human Integrated Protein-Protein Interaction rEference (HIPPIE) (http://cbdm.uni-mainz.de/hippie/) is an online tool providing experimental and predicted PPI information [31] through interfacing different data bases such as IntAct Molecular Interaction Database (https://www.ebi.ac.uk/intact/) [32], Biological General Repository for Interaction Datasets (BioGRID) (https://thebiogrid.org/) [33], The Human Protein Reference Database (HPRD) (http://www.hprd.org/) [34], the Molecular INTeraction database (MINT) (https://mint.bio.uniroma2.it/) [35], The Biomolecular Interaction Network Database (BIND) (http://baderlab.org/BINDTranslation) [36], MIPS (http://mips.helmholtz-muenchen.de/proj/ppi/) [37] and DIP (http://dip.doe-mbi.ucla.edu/dip/Main.cgi) [38]. In this study, the HIPPIE [31] was used to analyze the PPIs among the proteins encoded by the DEGs, then the PPI networks for the up-regulated and the down-regulated genes are separately visualized by Cytoscape version 3.5.1 software (http://www.cytoscape.org/) [39]. The degree of a gene in a PPI network is equal to the number of edges containing that node [40]. Betweenness centrality of a gene which is located on the shortest path between two other genes has most influence over the “information transfer” between them [41]. Stress centrality is number of genes in the shortest path between two other genes [42]. Closeness centrality is an inverse of the average length of the shortest paths to/from all the other genes in the PPI network [43]. Cluster coefficient measures the density of interactions in the network neighborhood of a gene [44].

### 2.7. Module analysis

In PPI networks, genes in the same module typically show the same or similar function and work together to implement their biological function. To visualize the network and identify the modules in the network, PEWCC1 java plug-in [45] on the Cytospace software (www.cytoscape.org/) [39] was used. The parameters were set as follows: Degree cutoff ≥ 10 (degrees of each node in module were at least larger than 2), K-core ≥ 2 (subgraphs of each node in module were at least 2 and more than 2).

### 2.8. Construction of the target gene-miRNA regulatory network

The NetworkAnalyst (http://www.networkanalyst.ca/) is a online tool available comprehensive resource containing the predicted and the experimentally validated target gene-miRNA interaction pairs [46]. The DEGs-associated predicted miRNA were selected when they were included two TarBase (http://diana.imis.athena-innovation.gr/DianaTools/index.php?r=tarbase/index) [47] and miRTarBase (http://mirtarbase.mbc.nctu.edu.tw/php/download.php) [48]. Subsequently, the overlapping target genes were identified and the gene-miRNA pair was selected. The target gene-miRNA regulatory network was constructed and visualized using the Cytoscape version 3.5.1 software (http://www.cytoscape.org/) [39].

### 2.9. Construction of the target gene-regulatory TF network

The DEGs and transcription factors (TFs) that potentially regulated the DEGs are predicted using Overrepresentation Enrichment Analysis (ORA) in NetworkAnalyst (http://www.networkanalyst.ca/) [49]. The DEGs-associated predicted TF were selected when they were included database such as ENCODE (http://cistrome.org/BETA/) [50]. Then target gene-regulatory TF network was constructed and visualized using version 3.5.1 software (http://www.cytoscape.org/) [39].

### 2.10. Receiver operating characteristic (ROC) curve analysis

Receiver operating characteristic (ROC) curve analysis was executed to calculate the sensitivity and specificity of the DEGs for pituitary prolactinoma diagnosis using the pROC package in R software [51]. An area under the curve (AUC) value was calculated and used to designate the ROC effect.

## 3. Results

### 3.1. Identification of DEGs

**Figure 1.**
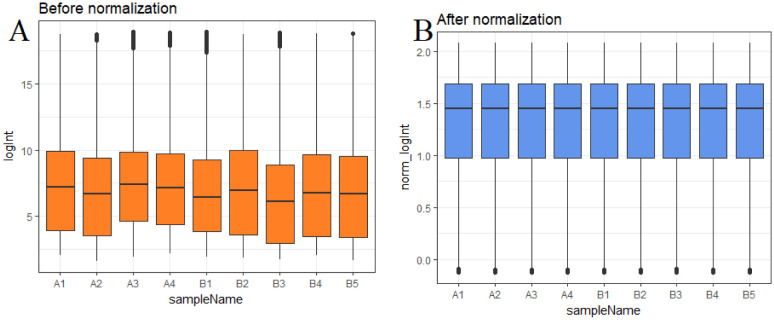
Box plots of the gene expression data before and after normalization. Horizontal axis represents the sample symbol and the vertical axis represents the gene expression values. The black line in the box plot represents the median value of gene expression. (A1, A2, A3, A4 = normal pituitaries samples; B1, B2, B3, B4, B5 = 5 pituitary prolactinoma).

**Figure 2.**
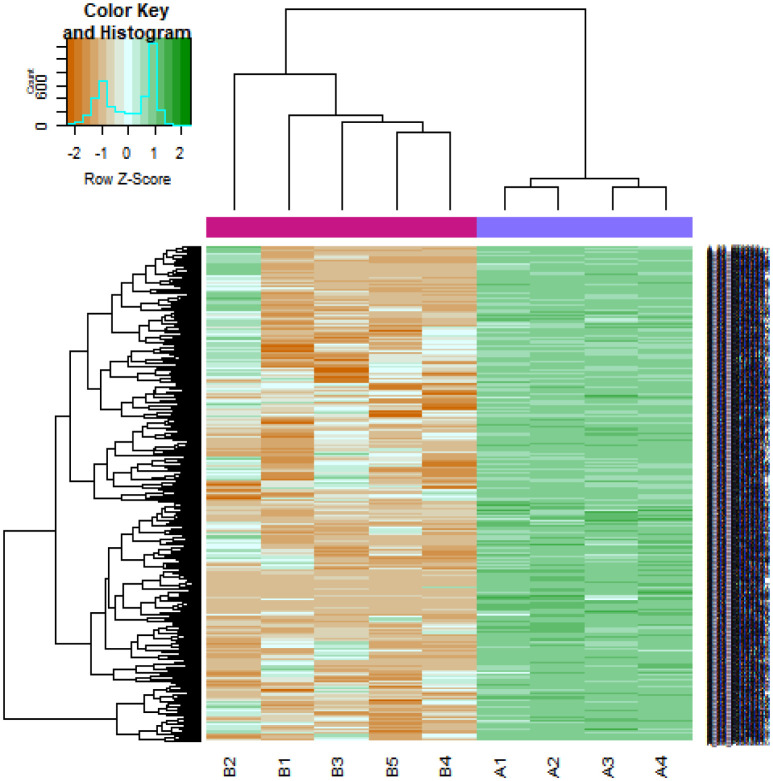
Heat map of up regulated differentially expressed genes. Legend on the top left indicate log fold change of genes. (A1, A2, A3, A4 = normal pituitaries samples; B1, B2, B3, B4, B5 = 5 pituitary prolactinoma).

**Figure 3.**
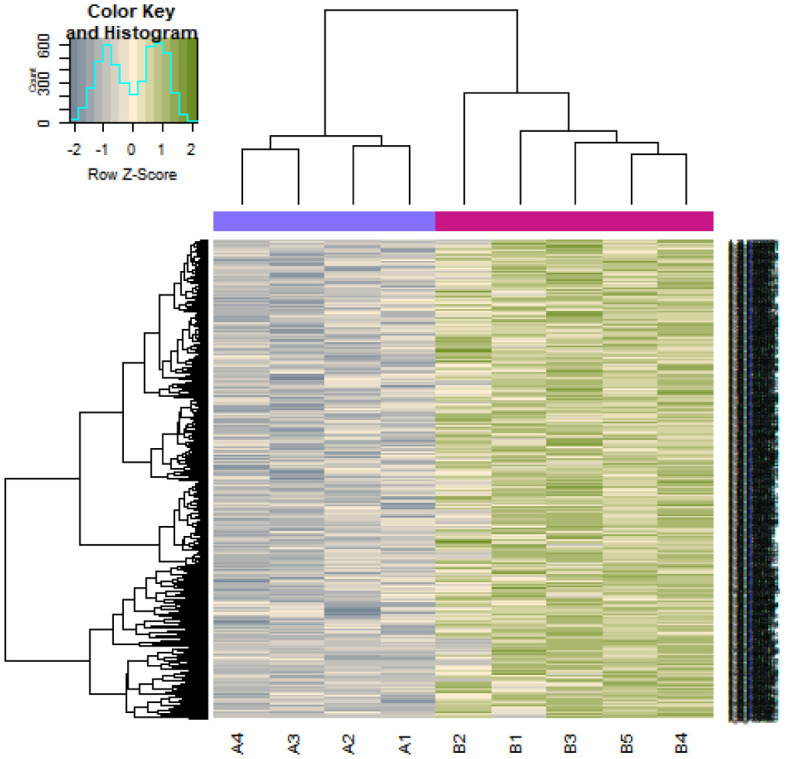
Heat map of down regulated differentially expressed genes. Legend on the top left indicate log fold change of genes. (A1, A2, A3, A4 = normal pituitaries samples; B1, B2, B3, B4, B5 = 5 pituitary prolactinoma).

**Figure 4.**
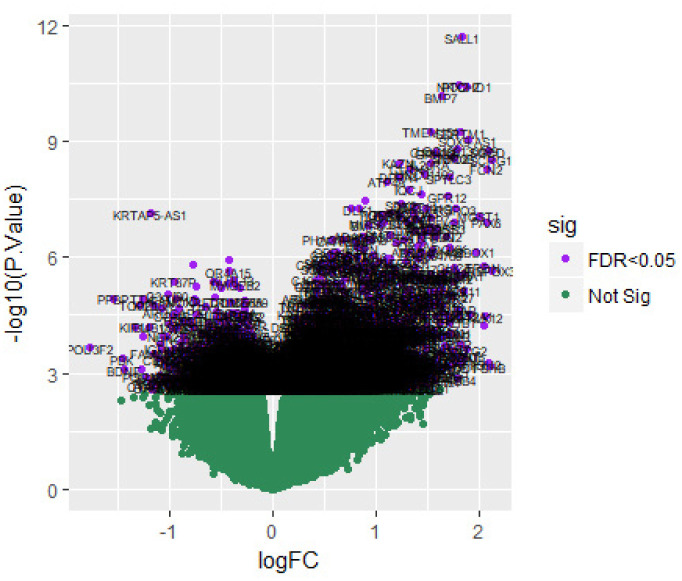
Volcano plot of differentially expressed genes. Genes with a significant change of more than two-fold were selected.

After data preprocessing, the raw data of nine samples is proved to be eligible (Figure 1). The GSE119063 expression profile data from GEO was investigated to screen for DEGs between the prolactinoma and normal groups. Under the threshold of FDR < 0.05, and fold change ≥0.93 for up regulated gene and fold change ≥−0.29 for down regulated gene. Comparison of prolactinoma with normal pituitaries identified total of 989 DEGs, including 461 up regulated genes and 528 down regulated genes, were revealed (Table 1, see the supplementary). A corresponding heat map is shown in Figure 2 and Figure 3. All the DEGs were presented by volcano plot in the study (Figure 4).

### 3.2. Pathway enrichment analysis of DEGs

Several significant enriched pathways are acquired through BIOCYC, KEGG, PID, REACTOME, GenMAPP, MSigDB C2 BIOCARTA, PantherDB, Pathway Ontology and SMPDB pathway enrichment analysis (Table 2 and Table 3, see the supplementary). The top enriched pathways for up regulated genes included retinoate biosynthesis II, retinoate biosynthesis I, signaling pathways regulating pluripotency of stem cells, neuroactive ligand-receptor interaction, ALK2 signaling events, BMP receptor signaling, peptide hormone biosynthesis, glycoprotein hormones, tyrosine metabolism, androgen and estrogen metabolism, ensemble of genes encoding extracellular matrix and extracellular matrix-associated proteins, genes encoding secreted soluble factors, adenine and hypoxanthine salvage pathway, 5-Hydroxytryptamine biosynthesis, melanocortin system, androgen and estrogen metabolic, tryptophan metabolism and xanthine dehydrogenase deficiency (Xanthinuria). Meanwhile, down regulated DEGs strikingly enriched in vitamin D3 biosynthesis, cell cycle, pancreatic secretion, aurora B signaling, FOXM1 transcription factor network, mitotic prometaphase, resolution of sister chromatid cohesion, role of ran in mitotic spindle regulation, Eph kinases and ephrins support platelet aggregation, inflammation mediated by chemokine and cytokine signaling pathway, o-glycans biosynthetic, ganglioside biosynthetic, eptifibatide pathway and ticlopidine pathway.

### 3.3. Gene Ontology (GO) enrichment analysis of DEGs

All significant DEGs were divided into up regulated genes and down regulated genes. GO categories analyses are conducted for these 2 lists of genes, respectively. Results of GO categories are presented by 3 functional groups, which are group BP, CC, and MF (Table 4 and Table 5, see the supplementary). In group BP, up and down regulated DEGs are significantly enriched in sensory organ morphogenesis, embryonic organ morphogenesis, nuclear division and organelle fission. For group CC, up and down regulated DEGs mainly enriched in extracellular matrix and extracellular space, condensed chromosome and kinetochore. In addition, GO results of group MF showed that up and down regulated DEGs mainly enriched in hormone activity, signaling receptor binding, microtubule binding and microtubule motor activity.

### 3.4. PPI network construction and topology analysis

**Figure 5.**
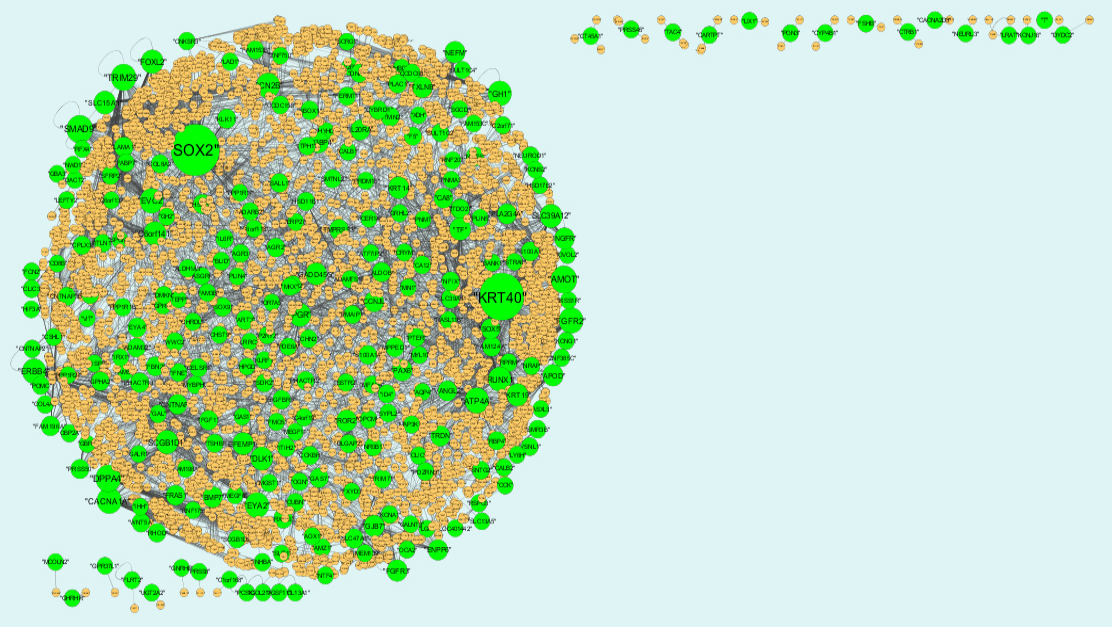
Protein-protein interaction network of differentially expressed genes (DEGs). Green nodes denotes up regulated genes.

**Figure 6.**
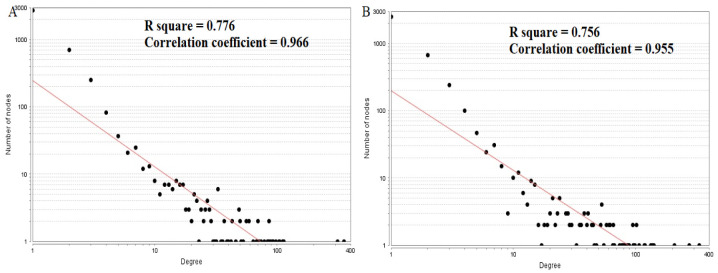
Node degree distribution (A: Up regulated genes; B: Down regulated genes).

**Figure 7.**
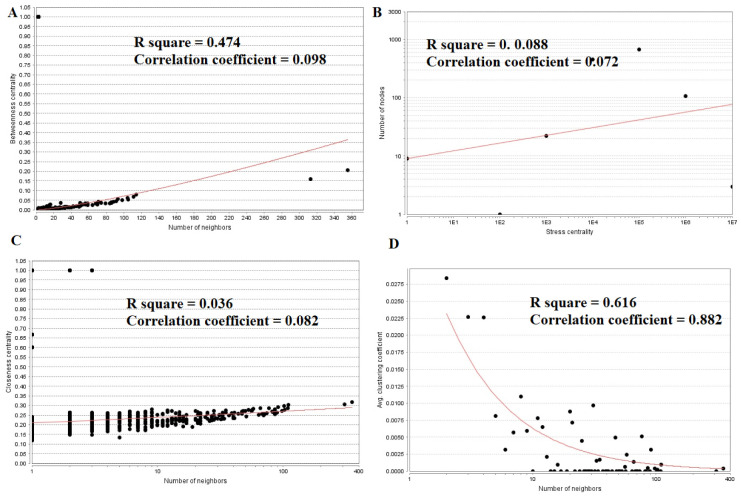
Regression diagrams for up regulated genes (A: Betweenness centrality; B: Stress centrality; C: Closeness centrality; D: Clustering coefficient).

PPI networks were constructed on the basis of HIPPIE online tool. We also analyzed the network properties such as node degree, betweenness centrality, stress centrality, closeness centrality and cluster coefficient. The PPI network for up regulated DEGs is shown in Figure 5, which has 4005 nodes and 5562 interactions. The top 5 nodes with greater degrees are listed in Table 6 (see the supplementary), including SOX2 (degree = 355), KRT40 (degree = 313), SMAD9 (degree = 114), AMOT (degree = 111) and DPPA4 (degree = 105). R square and correlation coefficient are 0.776 and 0.966, respectively (Figure 6A). Top 5 up regulated genes with high betweenness centrality are SOX2 (betweeness = 0.20715558), KRT40 (betweeness = 0.15884752), SMAD9 (betweeness = 0.07865032), AMOT (betweeness = 0.06814966) and TRIM29 (betweeness = 0.0613444) shown in Table 6. R square and correlation coefficient are 0.474 and 0.098, respectively (Figure 7A). Top 5 up regulated high stress genes are SOX2 (stress = 37524600), KRT40 (stress = 22565808), AMOT (stress = 13889668), C6orf141 (stress = 9207512) and TRIM29 (stress = 9041322) shown in Table 6. R square and correlation coefficient are 0.088 and 0.072, respectively (Figure 7B). Top 5 up regulated gene with high closeness centrality are SOX2 (closeness = 0.31817447), KRT40 (closeness = 0.30482897), SMAD9 (closeness = 0.30309326), TRIM29 (closeness = 0.2975525) and FGFR2 (closeness = 0.29117386) shown in Table 6. R square and correlation coefficient are 0.036 and 0.082, respectively (Figure 7C). Top 5 up regulated gene with low clustering coefficient are PRSS45 (clustering coefficient = 0), CARTPT (clustering coefficient = 0), TAC4 (clustering coefficient = 0), CT45A1 (clustering coefficient = 0) and PON3 (clustering coefficient = 0) shown in Table 6. R square and correlation coefficient are 0.616 and 0.882, respectively (Figure 7D).

**Figure 8.**
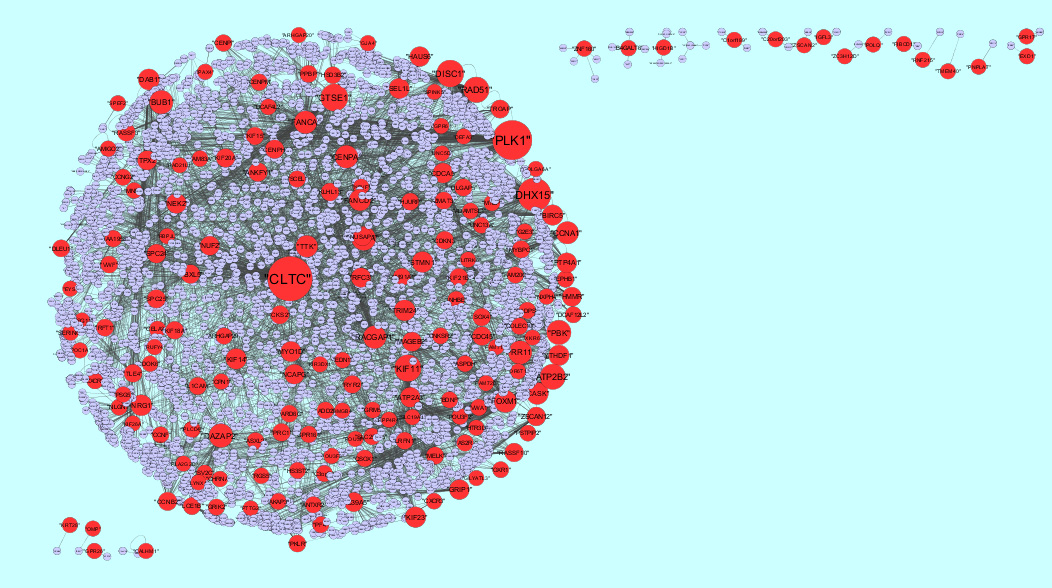
Protein-protein interaction network of differentially expressed genes (DEGs). Red nodes denotes up regulated genes.

**Figure 9.**
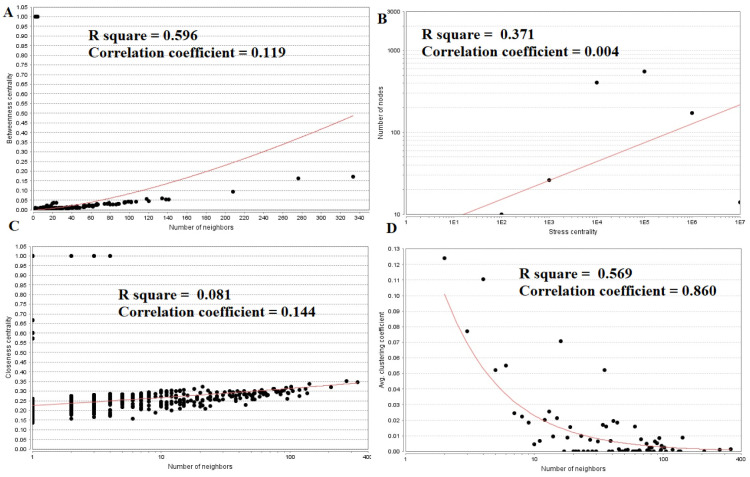
Regression diagrams for down regulated genes (A: Betweenness centrality; B: Stress centrality; C: Closeness centrality; D: Clustering coefficient).

The PPI network for down regulated DEGs is shown in Figure 8, which has 5441 nodes and 9866 interactions. The top 5 nodes with grater degrees are listed in Table 6, including CLTC (degree = 333), PLK1 (degree = 276), DHX15 (degree = 208), GTSE1 (degree = 141) and DISC1 (degree = 138). R square and correlation coefficient are 0.756 and 0.955, respectively (Figure 6B). Top 5 down regulated genes with high betweenness centrality are CLTC (betweeness = 0.17279707), PLK1 (betweeness = 0.16393694), DHX15 (betweeness = 0.09387272), KIF11 (betweeness = 0.05972209) and ATP2B2 (betweeness = 0.0557287) shown in Table 6. R square and correlation coefficient are 0.596 and 0.119, respectively (Figure 9A). Top 5 down regulated genes with high stress genes are CLTC (stress = 59969246), DHX15 (stress = 39966314), PLK1 (stress = 39488508), ATP2B3 (stress = 23062588) and KIF11 (stress = 17181634) shown in Table 6. R square and correlation coefficient are 0.371 and 0.004, respectively (Figure 9B). Top 5 down regulated genes with high closeness centrality are PLK1 (closeness = 0.35244041), CLTC (closeness = 0.34583256), ATP2B4 (closeness = 0.33762233), ATP2B8 (closeness = 0.32332523) and DHX15 (closeness = 0.32084733) shown in Table 6. R square and correlation coefficient are 0.081 and 0.144, respectively (Figure 9C). Top 5 up regulated gene with low clustering coefficient are B4GALT6 (clustering coefficient = 0), ZNF160 (clustering coefficient = 0), HIGD1B (clustering coefficient = 0), CCL3L3 (clustering coefficient = 0) and C20orf203 (clustering coefficient = 0) shown in Table 6. R square and correlation coefficient are 0.569 and 0.860, respectively (Figure 9D).

### 3.5. Module analysis

**Figure 10.**
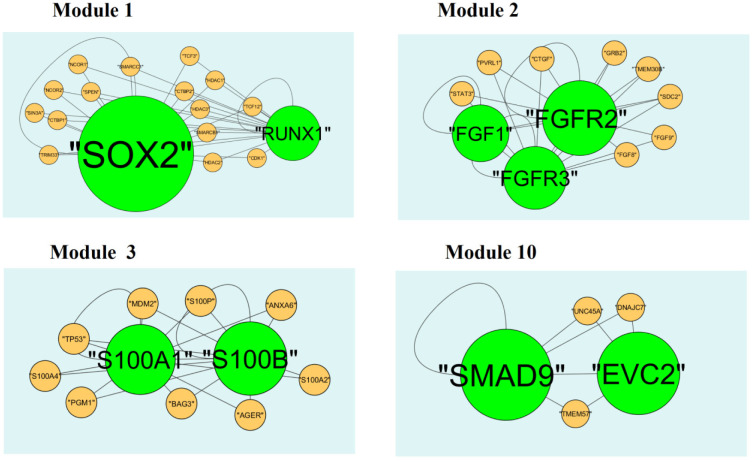
Modules in PPI network. The green nodes denote the up-regulated genes.

A total of 332 modules are identified in up regulated PPI network, among which the best are module 1, module 2, module 3 and module 10 (Figure 10). Module 1 is composed of 17 nodes and 33 edges. The hub proteins in this module such as RUNX1 (degree = 79) and SOX2 (degree = 355) are involved in module 1. Module 2 is composed of 11 nodes and 23 edges. The hub proteins in this module such as FGFR2 (degree = 93), FGF1 (degree = 27) and FGFR3 (degree = 49) are involved in module 2. Module 3 is composed of 11 nodes and 21 edges. The hub proteins in this module such as S100B (degree = 33) and S100A1 (degree = 23) are involved in module 3. Module 10 is composed of 5 nodes and 8 edges. The hub proteins in this module such as SMAD9 (degree = 114) and EVC2 (degree = 86) are involved in module 10.

**Figure 11.**
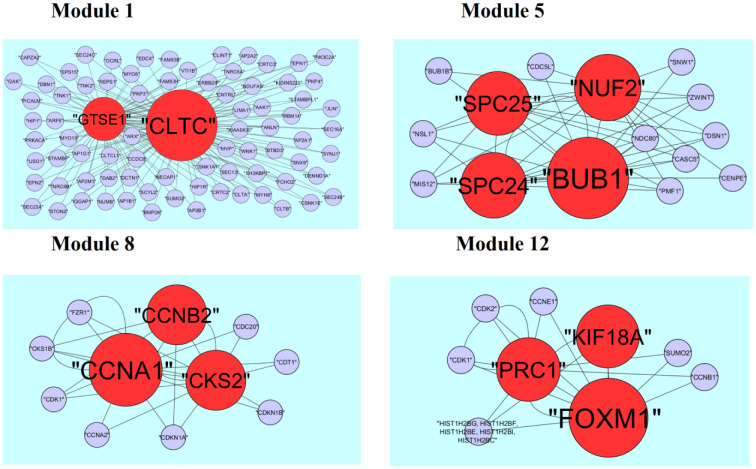
Modules in PPI network. The red nodes denote the down-regulated genes.

A total of 425 modules are identified in down regulated PPI network, among which the best are module 1, module 5, module 8 and module 12 (Figure 11). Module 1 is composed of 80 nodes and 157 edges. The hub proteins in this module such as CLTC (degree = 333) and GTSE1 (degree = 141) are involved in module 1. Module 5 is composed of 15 nodes and 43 edges. The hub proteins in this module such as NUF2 (degree = 44), BUB1 (degree = 95), SPC24 (degree = 41) and SPC25 (degree = 35) are involved in module 5. Module 8 is composed of 11 nodes and 23 edges. The hub proteins in this module such as CKS2 (degree = 36), CCNB2 (degree = 36) and CCNA1 (degree = 83) are involved in module 8. Module 12 is composed of 9 nodes and 17 edges. The hub proteins in this module such as KIF18A (degree = 27), FOXM1 (degree = 80) and PRC1 (degree = 29) are involved in module 12.

### 3.6. Construction of the target gene-miRNA network

**Figure 12.**
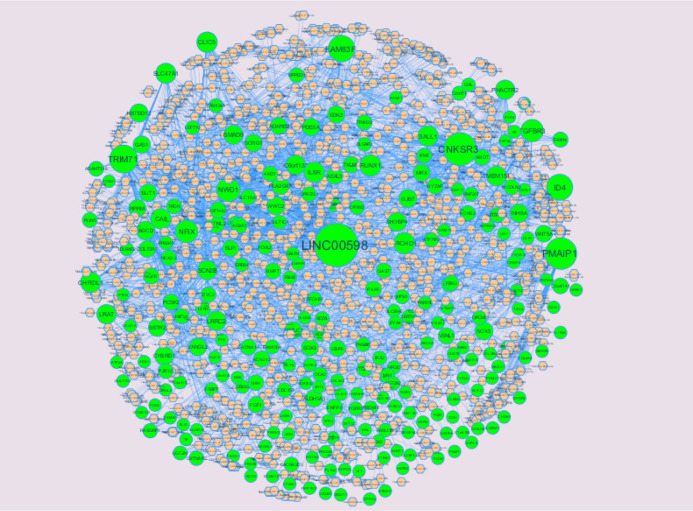
The network of up-regulated DEGs and their related miRNAs. The green circles nodes are the up regulated DEGs and pinkiamond nodes are the miRNAs.

**Figure 13.**
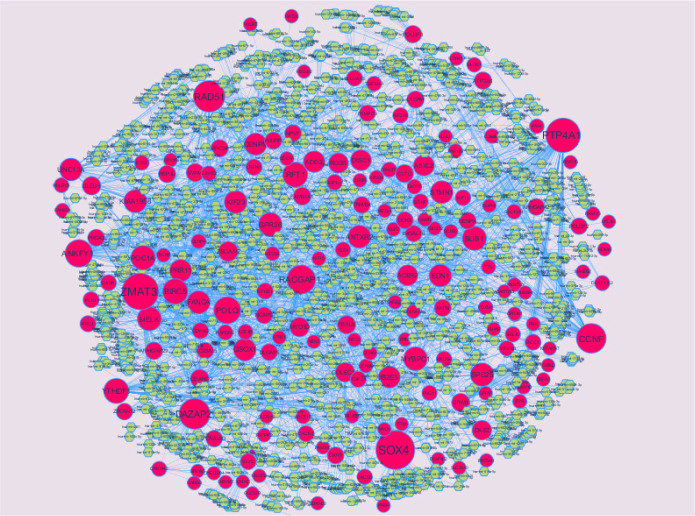
The network of up-regulated DEGs and their related miRNAs. The pink circles nodes are the up regulated DEGs and yellow diamond nodes are the miRNAs.

The miRNAs that may control the DEGs are diagnosed based on the up and down regulation expressions (Figure 12 and Figure 13). Top 5 up regulated targeted genes such as LINC00598 regulated by 209 miRNAs, CNKSR3 regulated by 138 miRNAs, PMAIP1 regulated by 128 miRNAs, TRIM71 regulated by 104 miRNAs and FAM83F regulated by 94 miRNAs are given in Table 7 (see the supplementary). Top 5 down regulated targeted genes such as SOX4 regulated by 160 miRNAs, ZMAT3 regulated by 145 miRNAs, PTP4A1 regulated by 132 miRNAs, RAD51 regulated by 113 miRNAs and DAZAP2 regulated by 109 miRNAs are given in Table 7.

### 3.7. Construction of the target gene-TF network

**Figure 14.**
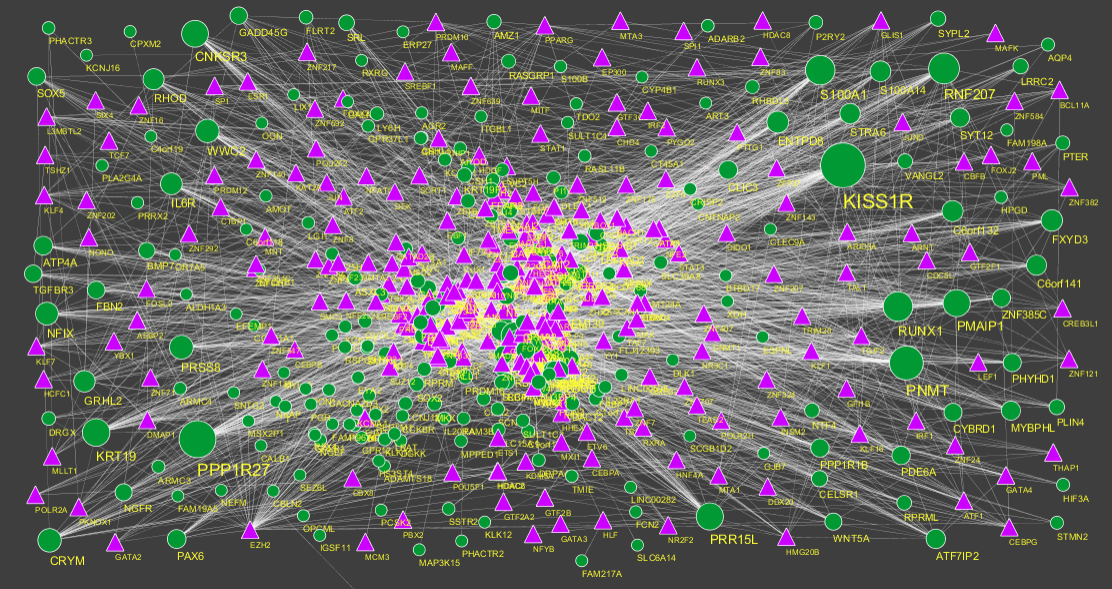
TF-gene network of predicted target up regulated genes. (Purple triangle-TFs and green circles-target up regulated genes).

**Figure 15.**
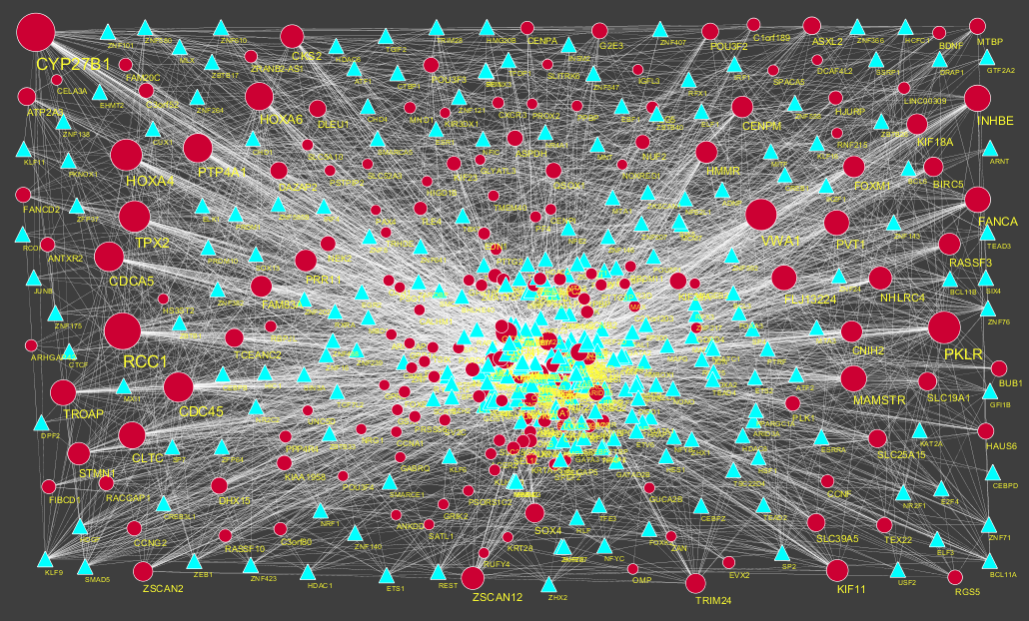
TF-gene network of predicted target up regulated genes. (Sky blue triangle-TFs and red circles-target up regulated genes).

The TFs for target up and down regulated genes are shown in Figure 14 and Figure 15, respectively. Top 5 up regulated targeted genes such as IRX1 regulated by 73 TFs, CACNA2D3 regulated by 30 TFs, VSNL1 regulated by 29 TFs, BMP7 regulated by 28 TFs and DACT2 regulated by 27 TFs are given in Table 8 (see the supplementary). Top 5 down regulated targeted genes such as UNC13A regulated by 43 TFs, CCNF regulated by 35 TFs, BDNF regulated by 34 TFs, POU3F4 regulated by 34 TFs and AKAP3 regulated by 31 TFs are given in Table 8.

### 3.8. Receiver operating characteristic (ROC) curve analysis

**Figure 16.**
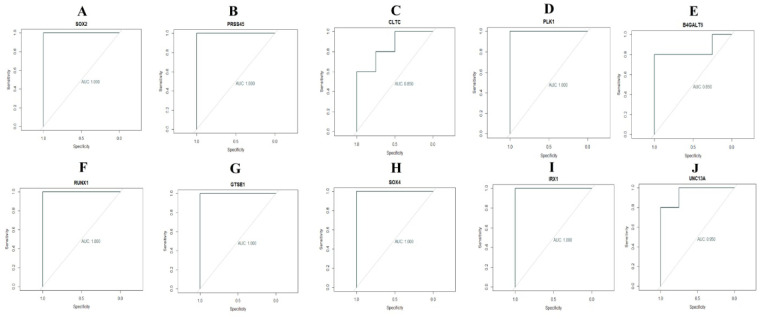
ROC curve analyses of hub genes. A) SOX2; B) PRSS45; C) CLTC; D) PLK1; E) B4GALT6; F) RUNX1; G) GTSE1; H) SOX4; I) IRX1; J) UNC13A.

As these 10 hub genes are prominently expressed in pituitary prolactinoma, we performed a ROC curve analysis to evaluate their sensitivity and specificity for the diagnosis of pituitary prolactinoma. As shown in Figure 16, SOX2, PRSS45, CLTC, PLK1, B4GALT6, RUNX1, GTSE1, SOX4, IRX1 and UNC13A achieved an AUC value of >0.9, demonstrating that these genes have high sensitivity and specificity for pituitary prolactinoma diagnosis. The results suggested that SOX2, PRSS45, CLTC, PLK1, B4GALT6, RUNX1, GTSE1, SOX4, IRX1 and UNC13A can be used as biomarkers for the diagnosis of pituitary prolactinoma.

## 4. Discussion

A molecular understanding of pituitary prolactinoma is particularly essential to eventually improve effective approaches for its control, treatment, and prevention. In the present study, important candidate genes of pituitary prolactinoma were identified by bioinformatics analysis. We downloaded the gene expression data from GSE119063 and obtained 461 up regulated and 528 down regulated genes in pituitary prolactinoma. SALL1 action as a tumor suppressor and epigenetic inactivation of this gene is responsible for development of cancer [52], but this gene might be liable for pituitary prolactinoma. Wang et al. [53] showed that expression of homeodomain transcription factor NKX2-2 is linked with progression of neuro endocrine tumor, but this gene might be the key for progression of pituitary prolactinoma. High expression of pleiotropic signaling molecule BMP7 controls proliferation, migration, and invasion of cancer cells [54], but this gene might be linked with proliferation, migration, and invasion of pituitary prolactinoma cells. Cipriano et al. [55] identified that ontogeny FAM83F is associated with epithelial cell transformation in cancer, but involvement of this gene might be responsible for advancement of pituitary prolactinoma. Oncogene GRHL2 is a transcriptional controller of proliferation and differentiation in epithelial cells, both during progression and tumor development, but this gene might be linked with proliferation of pituitary prolactinoma cells [56]. TRIM24 play key role in proliferation and invasion of cancer cells [57], but high expression of this gene might be responsible for invasion of pituitary prolactinoma cells. Kaistha et al. [58] describe that high expression of dual specificity kinase TTK shows proliferative potential of cancer cells, but this gene might be linked with proliferation of pituitary prolactinoma cells. Over expression of TOP2A is responsible for growth of cancer [59], but increase expression of this gene might associate with growth of pituitary prolactinoma. Potential role KIF18A is associated with cell division and checkpoint activation in cancer progression [60], but this gene might be responsible for cell division and checkpoint activation in pituitary prolactinoma. Over expression of microtubule associated protein TPX2 stimulates the cell cycle in cancer [61], but this gene might be activates cell cycle in pituitary prolactinoma. Genes such as FGFR2 [62], SOX2 [63], POMC (proopiomelanocortin) [64], FSHB (follicle stimulating hormone beta subunit) [65], EFEMP1 [66], SFRP2 [67], CSH2 [68], IHH (indian hedgehog) [69], GH1 [70], PTTG2 [71], CCNB2 [72], RACGAP1 [73], CXCR2 [74], CXCR3 [75], FOXL2 [76], RUNX2 [77], TF (transferrin) [78], CCK (cholecystokinin) [79], DPPA4 [80], RUNX1 [81] and BDNF (brain derived neurotrophic factor) [82] are associated with progression of pituitary prolactinoma.

In present study indicated that retinoate biosynthesis II is the most significant BIOCYC pathway for up regulated genes. XDH and RBP4 are novel biomarkers for pathogenesis of pituitary prolactinoma. Signaling pathways regulating pluripotency of stem cells is the most significant KEGG pathway for up regulated genes. Alteration in FGFR3 is responsible for progression of bladder cancer [83], but this gene might be important for development of pituitary prolactinoma. WNT5A was linked with invasion of cancer cells [84], but this gene might be involved in invasion of pituitary prolactinoma cells. ID4 is potential tumor suppressor and epigenetic inactivation of this gene linked with progression of prostate cancer [85], but loss gene this might be liable for advancement of pituitary prolactinoma. High expression of INHBA (inhibin beta A subunit) linked with cell proliferation in lung adenocarcinoma [86], but this gene might be associated with cell proliferation in pituitary prolactinoma. Epigenetic inactivation of PAX6 gene was liable for progression of breast cancer [87], but loss this gene might be culpable for progression of pituitary prolactinoma. LEFTY2 and SMAD9 are novel biomarkers for pathogenesis of pituitary prolactinoma. ALK2 signaling events are the most significant PID pathway for up regulated genes. Peptide hormone biosynthesis is the most significant REACTOME pathway for up-regulated genes. CGB3 and TSHB are novel biomarkers for pathogenesis of pituitary prolactinoma. Tyrosine metabolism was the most significant GenMAPP pathway for up regulated genes. Duan et al. [88] showed that ALDH1A3 play a regulatory role in the initiation and development of cancer, but this gene might be linked with pathogenesis of pituitary prolactinoma. PNMT and AOX1 are novel biomarkers for pathogenesis of pituitary prolactinoma. Ensemble of genes encoding extracellular matrix and extracellular matrix-associated proteins is the most significant MSigDB C2 BIOCARTA pathway for up regulated genes. S100A14 is a modulator of HER2 signaling pathway in breast cancer [89], but this gene might be responsible for development of pituitary prolactinoma. DeRycke et al. [90] found that high expression of S100A1 is identified with ovarian cancer, but this gene might be important for progression of pituitary prolactinoma. Harpio and Einarsson [91] identified that high level of S100B is answerable for development of melanoma, but this gene might be identified with pituitary prolactinoma. Methylation inactivation of FBN2 was associated with invasion and metastasis of non-small cell lung cancer [92], but loss of this gene might be involved in pathogenesis of pituitary prolactinoma. Yoshimura et al. [93] reported that FGF1 is an inducer of angiogenesis in breast cancer, but this gene might associated with angiogenesis in pituitary prolactinoma. SLIT1 is tumor suppressor gene and epigenetic suppression of this gene is associated with cancer progression [94], but loss of this gene might be culpable for development pituitary prolactinoma. ADAMTS18 and CHRDL1 are putative tumor suppressor genes and epigenetic silencing of these genes diagnosed with various cancer types [95]–[96], but inactivation of these genes might be linked with progression of pituitary prolactinoma. VIT, MFAP4, FCN2, SBSPON, SPON1, ITIH6, COL4A6, COL8A2, FRAS1, COL13A1, ITLN1, ADAM32, CSH1, CSHL1, SLPI, IFNE, MEGF11, LAMA1, ITIH2, GH2, COL21A1, CLEC9A, NTF4, OGN, RSPO3, MEGF6, CBLN2 and LGI1 are novel biomarkers for pathogenesis of pituitary prolactinoma. Adenine and hypoxanthine salvage pathway is the most significant PantherDB pathway for up-regulated genes. Melanocortin system is the most significant Pathway Ontology for up-regulated genes. PCSK2 is novel biomarker for pathogenesis of pituitary prolactinoma. Tryptophan metabolism is the most significant PantherDB pathway for up-regulated genes. TDO2 and TPH1 are novel biomarkers for pathogenesis of pituitary prolactinoma. Vitamin D3 biosynthesis is the most significant BIOCYC pathway for down-regulated genes. Polymorphisms of CYP27B1 is responsible for progression of colon cancer [97], but this polymorphic gene might be responsible for growth of pituitary prolactinoma. Cell cycle is the most significant KEGG pathway for down-regulated genes. PLK1 is linked with proliferation of cancer cells [98], but this might be gene is liable for proliferation of pituitary prolactinoma. Mitotic spindle checkpoint gene BUB1 is linked with proliferation of gastric cancer cells [99], but this gene might be responsible for proliferation of pituitary prolactinoma cells. Epigenetic inactivation of CCNA1 is responsible for advancement of cervical cancer [100], but silencing of this gene might be responsible for progression of pituitary prolactinoma. CDC45 is novel biomarker for pathogenesis of pituitary prolactinoma. Aurora B signaling is the most significant PID pathway for down-regulated genes. KIF23 plays key role in progression of lung cancer [101], but this gene might play crucial role in pituitary prolactinoma. BIRC5 play key role in cell division and proliferation of liver cancer cells [102], but this gene might be linked with cell division and proliferation in pituitary prolactinoma. NCAPG associated with cell proliferation and migration of liver cancer cells [103], but this gene might be linked with cell proliferation and migration of pituitary prolactinoma cells. Nie et al. [104] suggest that STMN1 play essential role in the control of cellular division and proliferation in non-small cell lung cancer, but this gene might be linked with cellular division and proliferation in pituitary prolactinoma. KIF20A is associated with progression of pancreatic cancer [105], but this gene might be play key role in progression of pituitary prolactinoma. CENPA and KLHL13 are novel biomarkers for pathogenesis of pituitary prolactinoma. Mitotic prometaphase is the most significant REACTOME pathway for down-regulated genes. SPC24 and SPC25 are associated with genomic instability and disrupted regulation of cell cycle in lung cancer, but this gene might be responsible for progression of pituitary prolactinoma [106]. Cell cycle marker CENPH pay key role in proliferation of gastric cancer cells [107], but this gene might be involved in proliferation of pituitary prolactinoma. NUF2 activates tumor growth and inhibits cell apoptosis [108], but this gene might be liable for pathogenesis of pituitary prolactinoma. CENPM, CENPI, CDCA5 and CENPU are novel biomarkers for pathogenesis of pituitary prolactinoma. Role of Ran in mitotic spindle is the most significant MSigDB C2 BIOCARTA pathway for down-regulated genes. RCC1 and KIF15 are novel biomarkers for pathogenesis of pituitary prolactinoma. Inflammation mediated by chemokine and cytokine signaling pathway is the most significant PantherDB pathway for down-regulated genes. PF4-active platelet accumulation in cancer is crucial because platelets can modulate cancer cells and the cancer microenvironment to stimulate lung cancer outgrowth [109], but this gene might be answerable for development of pituitary prolactinoma. VWF, CCL3L3, CASK and PLCD4 are novel biomarkers for pathogenesis of pituitary prolactinoma. O-glycans biosynthetic is the most significant Pathway Ontology for down-regulated genes. B4GALT6 is novel biomarker for pathogenesis of pituitary prolactinoma. Eptifibatide pathway is the most significant SMPDB pathway for down-regulated genes. RYR2 is novel biomarker for pathogenesis of pituitary prolactinoma.

In present study, sensory organ morphogenesis is the most significant GO BP term for up-regulated genes. Epigenetic inactivation of tumor suppressor genes EYA4 and GAS1 are associated with progression of various cancer types, but silencing of these genes might associate with progression of pituitary prolactinoma [109]–[111]. Expression of SOX9 enhances the invasion and migration of colorectal cancer cells [112], but this gene might be linked with invasion and migration of pituitary prolactinoma cellc. ROR2 play a key role as an important mediator of the Wnt signaling pathway in colorectal cancer [113], but this gene might be involves in pathogenesis of pituitary prolactinoma through activation of Wnt signaling pathway. CLIC5, TMIE, CLRN1, PRRX2, SDK2, CELSR1, STRA6, CALB1 and VANGL2 are novel biomarkers for pathogenesis of pituitary prolactinoma. Extracellular matrix is the most significant GO CC term for up-regulated genes. FLRT2, CPXM2, LAD1 and TGFBR3 are novel biomarkers for pathogenesis of pituitary prolactinoma. Hormone activity is the most significant GO MF term for up-regulated genes. CARTPT, GPHA2 and GAL are novel biomarkers for pathogenesis of pituitary prolactinoma. Nuclear division is the most significant GO BP term for down-regulated genes. NUSAP1 was linked with proliferation and invasion of prostate cancer cells [114], but this gene might be responsible for proliferation and invasion of pituitary prolactinoma cells. Cell cycle regulatory protein CKS2 is culpable for advancement of gastric cancer [115], but this gene might be linked with pathogenesis of pituitary prolactinoma. PBK is likely to play a crucial role in cell division and cytokinesis in breast cancer [116], but this gene might be liable for cell division and cytokinesis in pituitary prolactinoma. Inappropriate expression of NEK2 interfere with mitotic processes results in breast cancer development [117], but this gene might be play key role in pathogenesis of pituitary prolactinoma. Oncogene KIF14 play crucial role in cancer development [118], but this gene might be associates with progression of pituitary prolactinoma. Wong et al. [119] and Loveday et al. [120] demonstrated that modification of normal DNA repair function of RAD51 and RAD51D might lead to genomic instabilities that eventually contribute to development of various cancer types, but gene might be identify with progression of pituitary prolactinoma. CCNG2 deeply involved in progression of pancreatic cancer via cell proliferation, invasion, chemoresistance, and differentiation [121], but this gene might be responsible for cell proliferation, invasion, chemoresistance, and differentiation in pituitary prolactinoma. FANCA, FANCD2, CLTC, MTBP, HAUS6, DLGAP5, RAD21L1, PTTG3P, KIF11, PRC1, EDN1 and CCNF are novel biomarkers for pathogenesis of pituitary prolactinoma. Condensed chromosome is the most significant GO CC term for down-regulated genes. HJURP is novel biomarker for pathogenesis of pituitary prolactinoma. Microtubule binding is the most significant GO MF term for down-regulated genes. KIF21B and KIF26A are novel biomarkers for pathogenesis of pituitary prolactinoma.

In present study, SOX2, KRT40, SMAD9, AMOT and DPPA4 were identified as hub proteins (up regulated DEGs) in the PPI network. AMOT control of the Hippo/LATS pathway in the processes of cell proliferation, motility, and differentiation in cancer [122], but this might be linked with cell proliferation, motility, and differentiation in pituitary prolactinoma. SOX2, KRT40, SMAD9, AMOT and TRIM29 are the hub proteins (up regulated DEGs) with highest betweenness centrality in the PPI network. TRIM29 is important in differentiation, proliferation, and development of gastric cancer [123], but this gene might be responsible for differentiation and proliferation in pituitary prolactinoma. SOX2, KRT40, AMOT, C6orf141 and TRIM29 are the hub proteins (up regulated DEGs) with highest stress centrality in the PPI network. C6orf141 is novel biomarkers for pathogenesis of pituitary prolactinoma. SOX2, KRT40, SMAD9, TRIM29 and FGFR2 are the hub proteins (up regulated DEGs) with highest closeness centrality in the PPI network. PRSS45, CARTPT, TAC4, CT45A1 and PON3 are the hub proteins (up regulated DEGs) with lowest clustering coefficient in the PPI network. Shang et al. [124] reported that proto-oncogene CT45A1 play key role in invasion of cancer cell, but this gene might be linked with invasion of pituitary prolactinoma cells. PRSS45, TAC4 and PON3 are novel biomarkers for pathogenesis of pituitary prolactinoma. CLTC, PLK1, DHX15, GTSE1 and DISC1 are identified as hub proteins (down regulated DEGs) in the PPI network. DHX15 play key role in cancer progression through activating AR activity through Siah2-mediated ubiquitination independent of its ATPase activity [125], but this gene might be liable for development of pituitary prolactinoma. Subhash et al. [126] shown that GTSE1 is affecting apoptosis in gastric cancer, but this gene might be answerable for development of pituitary prolactinoma. DISC1 is novel biomarker for pathogenesis of pituitary prolactinoma. CLTC, PLK1, DHX15, KIF11 and ATP2B2 are the hub proteins with highest betweenness centrality in the PPI network for down regulated DEGs. ATP2B2 is novel biomarker for pathogenesis of pituitary prolactinoma. CLTC, DHX15, PLK1, ATP2B3 and KIF11 are the hub proteins with highest stress centrality in the PPI network for down regulated DEGs. ATP2B3 is novel biomarker for pathogenesis of pituitary prolactinoma. PLK1, CLTC, ATP2B4, ATP2B8 and DHX15 are the hub proteins with highest closeness centrality in the PPI network for down regulated DEGs. ATP2B4 and ATP2B8 are novel biomarkers for pathogenesis of pituitary prolactinoma. B4GALT6, ZNF160, HIGD1B, CCL3L3 and C20orf203 are the hub proteins with lowest clustering coefficient in the PPI network for down regulated DEGs. ZNF160, HIGD1B and C20orf203 are novel biomarkers for pathogenesis of pituitary prolactinoma.

Modules are extracted from the PPI network for up and down regulated DEGs. RUNX1, SOX2, FGFR2, FGF1, FGFR3, S100B, S100A1, SMAD9 and EVC2 are the hub genes (up regulated DEGs with high degree) in all four modules in the PPI network. EVC2 is novel biomarker for pathogenesis of pituitary prolactinoma. CLTC, GTSE1, NUF2, BUB1, SPC24, SPC25, CKS2, CCNB2, CCNA1, KIF18A, FOXM1 and PRC1 are the hub genes (down regulated DEGs with high degree) in all four modules in the PPI network. FOXM1 play roles in cancer related processes, such as invasion and metastasis [127], but this might be linked with invasion and metastasis of pituitary prolactinoma.

LINC00598, CNKSR3, PMAIP1, TRIM71 and FAM83F are identified as up regulated target genes with high degree of connectivity in target gene-miRNA-regulatory network. LINC00598, CNKSR3, PMAIP1 and TRIM71 are novel biomarkers for pathogenesis of pituitary prolactinoma. SOX4, ZMAT3, PTP4A1, RAD51 and DAZAP2 are identified as down regulated target genes with high degree of connectivity in target gene-miRNA regulatory network. Oncogene SOX4 plays an essential role in prostate cancer progression [128], but this gene might be associated with progression of pituitary prolactinoma. PTP4A1 play key role in cancer cell growth and invasion of breast cancer cells [129], but this gene might be responsible for improvement of pituitary prolactinoma. ZMAT3 and DAZAP2 are novel biomarkers for invasion of pituitary prolactinoma.

IRX1, CACNA2D3, VSNL1, BMP7 and DACT2 are identified as up regulated target gene with high degree of connectivity in TFs-target gene regulatory network. CACNA2D3 and DACT2 are tumor suppressor genes and epigenetic inactivation of these genes are linked with progression of various cancer types [130],[131], but suppression of these genes might be identified with progression of pituitary prolactinoma. IRX1 and VSNL1 are novel biomarkers for pathogenesis of pituitary prolactinoma. UNC13A, CCNF, BDNF, POU3F4 and AKAP3 are identified as down regulated target genes with high degree of connectivity in TFs-target gene regulatory network. UNC13A, POU3F4 and AKAP3 are novel biomarkers for pathogenesis of pituitary prolactinoma.

## 5. Conclusions

We use bioinformatics analysis of pituitary prolactinoma to investigate the biological and clinical value genes. Finally, using a series of particular conditions we screened crucial genes from DEGs. These findings may improve our understanding of the etiology, pathology, and the potential molecular mechanisms and gene targets of pituitary prolactinoma, which may be beneficial for the identification of diagnostic biomarkers and treatment methods for pituitary prolactinoma. Nevertheless, lacking of experimental verification is a limitation of this study. Further molecular biological experiments *in vivo* and *in vitro* are required to confirm the function of the identified genes in pituitary prolactinoma.
