# Supplementary material for: Identification of potential and novel target genes in pituitary prolactinoma by bioinformatics analysis
Source: AIMS Neurosci. 2021 Feb 7;8(2):254–83. doi: 10.3934/Neuroscience.2021014 (PMC7940115; doi:10.3934/Neuroscience.2021014)
Supplement: Supplementary file 1 [file neurosci-08-02-014-s001.pdf]

AIMS Neuroscience

AIMS Neuroscience, 8(2): 254–283.
DOI: 10.3934/Neuroscience.2021014
Received: 05 November 2020
Accepted: 29 January 2021
Published: 07 February 2021

http://www.aimspress.com/journal/aimsph

Research article

Identification of potential and novel target genes in pituitary prolactinoma by bioinformatics analysis

Vikrant Ghatnatti[1], Basavaraj Vastrad[2], Swetha Patil[3], Chanabasayya Vastrad[4,*] and Iranna Kottursheti[5]

[1] Department of Endocrinology/Medicine, J N Medical College, Belagavi and KLE Academy of Higher Education & Research 590010, Karnataka, India
[2] Department of Biochemistry, Basaveshwar College of Pharmacy, Gadag, Karnataka 582103, India
[3] Department of Obstetrics and Gynaecology, J N Medical College, Belagavi and KLE Academy of Higher Education & Research 590010, Karnataka, India
[4] Biostatistics and Bioinformatics, Chanabasava Nilaya, Bharthinagar, Dharwad 580001, Karanataka, India
[5] Department of Ayurveda, Rajiv Gandhi Education Society's Ayurvedic Medical College, Ron 562209, Karanataka, India

* **Correspondence:** Email: channu.vastrad@gmail.com; Tel: 919480073398.

Supplementary

Table 1. The statistical metrics for key differentially expressed genes (DEGs).

Agilent Id	Gene Symbol	Log FC	P Value	FDR	T value	Up/Do-wn	Gene Name
A_23_P328074	SALL1	1.833089	2.06E-12	8.77E-08	60.26576	Up	spalt like transcription factor 1
A_23_P17522	NKX2-2	1.814078	3.68E-11	5.54E-07	42.7718	Up	NK2 homeobox 2
A_32_P149435	PTCHD1	1.891448	3.91E-11	5.54E-07	42.46841	Up	patched domain containing 1

A_23_P68487	BMP7	1.638544	7.17E-11	7.63E-07	39.50907	Up	bone morphogenetic protein 7
A_23_P361584	TMEM154	1.53654	5.79E-10	4.13E-06	30.7916	Up	transmembrane protein 154
A_24_P366607	SERTM1	1.817216	5.83E-10	4.13E-06	30.77075	Up	serine rich and transmembrane domain containing 1
A_24_P497464	SOX9-AS1	1.905462	9.38E-10	5.7E-06	29.06852	Up	SOX9 antisense RNA 1
A_33_P3240747	FAM83F	1.788916	1.62E-09	8.1E-06	27.23397	Up	family with sequence similarity 83 member F
A_32_P4595	SGCD	2.094348	1.81E-09	8.1E-06	26.87211	Up	sarcoglycan delta
A_32_P182299	C1orf168	1.588213	1.93E-09	8.1E-06	26.65684	Up	chromosome 1 open reading frame 168
A_24_P35228	GRHL2	1.574365	2.1E-09	8.1E-06	26.40034	Up	grainyhead like transcription factor 2
A_23_P80974	TDO2	1.745047	2.66E-09	9.04E-06	25.6513	Up	tryptophan 2,3-dioxygenase
A_23_P29965	SMR3B	1.759372	2.76E-09	9.04E-06	25.54097	Up	submaxillary gland androgen regulated protein 3B
A_23_P167159	SCRG1	2.129388	3.06E-09	9.29E-06	25.23178	Up	stimulator of chondrogenesis 1
A_24_P331830	KAZN	1.227017	3.59E-09	1.02E-05	24.75271	Up	kazrin, periplakin interacting protein
A_23_P145514	IL20RA	1.535447	3.86E-09	1.03E-05	24.53233	Up	interleukin 20 receptor subunit alpha
A_23_P328709	CLRN1	1.340457	5.2E-09	1.27E-05	23.67443	Up	clarin 1
A_23_P216756	FCN2	2.077085	5.38E-09	1.27E-05	23.57765	Up	ficolin 2
A_24_P709476	LINC01102	1.480536	6.97E-09	1.56E-05	22.85332	Up	long intergenic non-protein coding RNA 1102
A_23_P380526	DPPA4	1.231647	8.6E-09	1.78E-05	22.28297	Up	developmental pluripotency associated 4
A_24_P49260	SPTLC3	1.719134	8.8E-09	1.78E-05	22.2211	Up	serine palmitoyltransferase long chain base subunit 3
A_23_P430728	ATP4A	1.108847	1.14E-08	2.2E-05	21.5462	Up	ATPase H+/K+ transporting alpha subunit
A_33_P3250178	IQCJ	1.326735	1.73E-08	3.2E-05	20.48275	Up	IQ motif containing J
A_19_P00331971	MIR503HG	1.447267	2.34E-08	4.14E-05	19.75227	Up	MIR503 host gene
A_33_P3236833	GPR12	1.701415	2.66E-08	4.53E-05	19.44286	Up	G protein-coupled receptor 12
A_32_P194312	SDK2	1.254756	4.19E-08	6.6E-05	18.40361	Up	sidekick cell adhesion molecule 2
A_33_P3234060	SEPT14	1.389704	4.95E-08	7.45E-05	18.03658	Up	septin 14

A_33_P3608210	MIR31HG	1.48494	5.52E-08	7.45E-05	17.79865	Up	MIR31 host gene
A_23_P111402	RSPO3	1.785412	5.6E-08	7.45E-05	17.76502	Up	R-spondin 3
A_23_P66739	SLC13A5	1.374669	7.12E-08	9.03E-05	17.25669	Up	solute carrier family 13 member 5
A_23_P117464	TPPP2	1.134536	7.49E-08	9.03E-05	17.15035	Up	tubulin polymerization promoting protein family member 2
A_24_P206624	FGFR2	1.045116	7.8E-08	9.03E-05	17.06439	Up	fibroblast growth factor receptor 2
A_33_P3292432	IQCA1	1.083925	7.85E-08	9.03E-05	17.05136	Up	IQ motif containing with AAA domain 1
A_23_P36658	MGST1	2.006529	8.59E-08	9.49E-05	16.86567	Up	microsomal glutathione S-transferase 1
A_33_P3372954	LOC387720	1.229633	8.7E-08	9.49E-05	16.83959	Up	collagen alpha-1(X) chain
A_23_P134139	FABP7	1.539766	1E-07	0.000105	16.54886	Up	fatty acid binding protein 7
A_23_P215549	PON3	1.382761	1.02E-07	0.000105	16.52506	Up	paraoxonase 3
A_23_P374844	GAL	1.402474	1.07E-07	0.000109	16.41339	Up	galanin and GMAP prepropeptide
A_33_P3330149	PAX6	2.074082	1.27E-07	0.000123	16.08292	Up	paired box 6
A_33_P3385376	SYPL2	1.302737	1.28E-07	0.000123	16.06335	Up	synaptophysin like 2
A_33_P3345344	DRGX	1.08197	1.33E-07	0.000123	15.9954	Up	dorsal root ganglia homeobox
A_23_P127267	LGI1	1.756456	1.33E-07	0.000123	15.9945	Up	leucine rich glioma inactivated 1
A_33_P3402329	MIR503HG	1.05068	1.36E-07	0.000123	15.94943	Up	MIR503 host gene
A_33_P3373273	SLC15A1	1.538301	1.64E-07	0.000142	15.58775	Up	solute carrier family 15 member 1
A_23_P80162	TMPRSS3	1.596153	1.77E-07	0.000148	15.44677	Up	transmembrane protease, serine 3
A_19_P00318205	#N/A	1.577183	1.78E-07	0.000148	15.43499	Up	NA
A_23_P158318	ROR2	1.473452	1.95E-07	0.000157	15.26237	Up	receptor tyrosine kinase like orphan receptor 2
A_24_P289260	DACT2	1.389093	1.95E-07	0.000157	15.25987	Up	dishevelled binding antagonist of beta catenin 2
A_32_P111336	AL591242.1	1.506199	2.32E-07	0.000183	14.93773	Up	NA
A_24_P555066	SMTNL2	1.13931	2.61E-07	0.000202	14.72185	Up	smoothelin like 2
A_33_P3310894	KRT40	1.57768	2.81E-07	0.000208	14.59126	Up	keratin 40

A_23_P214026	FBN2	1.687663	2.83E-07	0.000208	14.57834	Up	fibrillin 2
A_23_P4899	NTF4	1.441871	2.84E-07	0.000208	14.57475	Up	neurotrophin 4
A_23_P18672	GBA3	0.964336	3.42E-07	0.000232	14.24096	Up	glucosylceramidase beta 3 (gene/pseudogene)
A_19_P00325499	#N/A	0.933759	3.44E-07	0.000232	14.23364	Up	NA
A_33_P3346073	SRL	1.279597	3.63E-07	0.000238	14.13739	Up	sarcalumenin
A_19_P00321712	AC112198.2	1.208537	4.08E-07	0.000259	13.93544	Up	NA
A_33_P3411021	SLIT1	1.437893	4.23E-07	0.000265	13.87466	Up	slit guidance ligand 1
A_23_P16630	OR7A5	1.409749	5.03E-07	0.00031	13.58235	Up	olfactory receptor family 7 subfamily A member 5
A_33_P3249768	DGKK	1.719184	5.8E-07	0.000347	13.34618	Up	diacylglycerol kinase kappa
A_33_P3342797	LOC401442	1.555765	6.8E-07	0.000396	13.08512	Up	uncharacterized LOC401442
A_23_P127495	BBOX1	1.969105	7.18E-07	0.00041	12.99661	Up	gamma-butyrobetaine hydroxylase 1
A_33_P3723448	ART3	1.310074	7.36E-07	0.000412	12.95714	Up	ADP-ribosyltransferase 3
A_33_P3275220	NRK	1.438252	7.49E-07	0.000414	12.93021	Up	Nik related kinase
A_33_P3322400	LOC654780	1.529021	8.77E-07	0.000472	12.67915	Up	splicing factor proline/glutamine-rich
A_33_P3363071	ITIH6	0.970677	9.01E-07	0.000478	12.63721	Up	inter-alpha-trypsin inhibitor heavy chain family member 6
A_19_P00316371	AC002480.2	1.144117	1.1E-06	0.000549	12.32129	Up	NA
A_33_P3229370	ID4	1.414675	1.11E-06	0.000549	12.31878	Up	inhibitor of DNA binding 4, HLH protein
A_33_P3282474	#N/A	1.105066	1.11E-06	0.000549	12.31117	Up	NA
A_33_P3246026	DEFB131	1.290266	1.31E-06	0.000615	12.05627	Up	defensin beta 131
A_33_P3400763	PLIN4	1.033075	1.33E-06	0.000616	12.03503	Up	perilipin 4
A_33_P3504002	LINC01299	1.129723	1.57E-06	0.000697	11.7883	Up	long intergenic non-protein coding RNA 1299
A_32_P324933	LINC00282	0.971613	1.72E-06	0.000748	11.65582	Up	long intergenic non-protein coding RNA 282
A_33_P3248799	TRDN	2.047985	1.75E-06	0.000749	11.63034	Up	triadin
A_23_P311895	CLIC5	1.76	1.86E-06	0.000777	11.54202	Up	chloride intracellular channel 5

A_33_P3391915	BTBD17	1.379368	1.97E-06	0.000812	11.45968	Up	BTB domain containing 17
A_33_P3315134	DIRC3	1.187589	2.04E-06	0.000812	11.41367	Up	disrupted in renal carcinoma 3
A_33_P3336257	IRX1	1.782642	2.19E-06	0.000854	11.31183	Up	iroquoishomeobox 1
A_33_P3316953	RBFOX3	2.121609	2.25E-06	0.000869	11.27381	Up	RNA binding protein, fox-1 homolog 3
A_23_P103765	FCER1A	1.675704	2.5E-06	0.000926	11.12106	Up	Fc fragment of IgE receptor Ia
A_33_P3240094	NGEF	1.065886	2.66E-06	0.000969	11.03844	Up	neuronal guanine nucleotide exchange factor
A_23_P162010	CCKBR	1.368159	2.67E-06	0.000969	11.03391	Up	cholecystokinin B receptor
A_23_P95790	ITLN1	1.534417	2.72E-06	0.00098	11.00679	Up	intelectin 1
A_23_P383227	S100A1	1.861029	2.85E-06	0.001006	10.94318	Up	S100 calcium binding protein A1
A_23_P42909	TMEM139	1.402909	2.87E-06	0.001006	10.93278	Up	transmembrane protein 139
A_23_P15889	CBLN2	1.141876	2.89E-06	0.001006	10.92424	Up	cerebellin 2 precursor
A_33_P3619171	PMAIP1	1.187979	3.01E-06	0.001041	10.86654	Up	phorbol-12-myristate-13-acetate-induced protein 1
A_23_P8497	GHRHR	1.170394	3.07E-06	0.001054	10.83768	Up	growth hormone releasing hormone receptor
A_33_P3270863	XDH	1.809518	3.15E-06	0.001071	10.80343	Up	xanthine dehydrogenase
A_24_P595717	TOGARAM2	1.604949	3.4E-06	0.001139	10.69969	Up	TOG array regulator of axonemal microtubules 2
A_33_P3350164	CCDC178	1.42881	3.44E-06	0.001144	10.68331	Up	coiled-coil domain containing 178
A_23_P203267	TRIM29	1.443956	3.54E-06	0.001159	10.64492	Up	tripartite motif containing 29
A_33_P3389237	UGT2A2	1.479469	3.6E-06	0.00117	10.62203	Up	UDP glucuronosyltransferase family 2 member A2
A_33_P3412262	JAKMIP2-AS1	1.32862	3.67E-06	0.001183	10.59731	Up	JAKMIP2 antisense RNA 1
A_24_P27234	SOX5	1.659349	3.82E-06	0.001212	10.54464	Up	SRY-box 5
A_33_P3409086	S100A1	1.494024	4.14E-06	0.001285	10.43768	Up	S100 calcium binding protein A1
A_32_P3476	RPRML	1.611243	4.32E-06	0.001322	10.38091	Up	reprimo like
A_33_P3773374	AC120036.5	1.753756	4.49E-06	0.001353	10.33104	Up	NA
A_23_P153155	GALR1	1.619947	4.67E-06	0.001358	10.27926	Up	galanin receptor 1

A_23_P83134	GAS1	1.058861	4.68E-06	0.001358	10.27589	Up	growth arrest specific 1
A_33_P3308964	PPP1R17	1.085168	4.71E-06	0.001358	10.26698	Up	protein phosphatase 1 regulatory subunit 17
A_23_P302060	IFNE	0.935355	4.82E-06	0.001371	10.23827	Up	interferon epsilon
A_23_P132378	CELSR1	1.253634	4.83E-06	0.001371	10.23304	Up	cadherin EGF LAG seven-pass G-type receptor 1
A_23_P29975	C4orf19	1.051466	5.25E-06	0.001459	10.12693	Up	chromosome 4 open reading frame 19
A_33_P3345036	POMC	1.014551	5.73E-06	0.001563	10.01362	Up	proopiomelanocortin
A_19_P00315473	#N/A	1.012926	6.47E-06	0.00171	9.859854	Up	NA
A_24_P365721	SLC6A14	1.636424	6.82E-06	0.00179	9.794187	Up	solute carrier family 6 member 14
A_32_P113066	LRAT	1.318608	6.87E-06	0.001793	9.784901	Up	lecithin retinol acyltransferase (phosphatidylcholine--retinol O-acyltransferase)
A_33_P3415395	SPTLC3	1.309174	7.32E-06	0.00185	9.704786	Up	serine palmitoyltransferase long chain base subunit 3
A_23_P101505	KLK11	1.847661	7.34E-06	0.00185	9.702092	Up	kallikrein related peptidase 11
A_32_P41604	F5	1.535516	7.35E-06	0.00185	9.700312	Up	coagulation factor V
A_33_P3367196	CNTNAP2	1.605568	7.42E-06	0.001856	9.688935	Up	contactin associated protein-like 2
A_33_P3215640	PI16	1.290311	7.68E-06	0.001911	9.645508	Up	peptidase inhibitor 16
A_24_P397255	ENPP6	1.594549	8.75E-06	0.002116	9.485334	Up	ectonucleotidepyrophosphatase/phosphodiesterase 6
A_33_P3217437	GBP6	1.036074	9.63E-06	0.002289	9.369347	Up	guanylate binding protein family member 6
A_33_P3351264	OVOL3	1.050788	9.78E-06	0.002311	9.351444	Up	ovo like zinc finger 3
A_23_P112327	OBP2A	1.350445	1E-05	0.002352	9.323343	Up	odorant binding protein 2A
A_23_P75283	RBP4	1.726183	1.04E-05	0.00242	9.282412	Up	retinol binding protein 4
A_33_P3252048	PPP1R27	1.719928	1.05E-05	0.002445	9.263686	Up	protein phosphatase 1 regulatory subunit 27
A_23_P202053	ITIH2	1.137137	1.1E-05	0.002526	9.21207	Up	inter-alpha-trypsin inhibitor heavy chain 2
A_23_P328740	NEURL3	1.378209	1.16E-05	0.002615	9.144984	Up	neuralized E3 ubiquitin protein ligase 3
A_23_P416774	CLIC5	1.122275	1.19E-05	0.002615	9.114478	Up	chloride intracellular channel 5
A_24_P13024	SLC16A12	1.528046	1.2E-05	0.002615	9.110565	Up	solute carrier family 16 member 12

A_33_P3287472	PPP1R27	1.441994	1.23E-05	0.002663	9.081115	Up	protein phosphatase 1 regulatory subunit 27
A_23_P69738	RASL11B	1.2602	1.25E-05	0.002678	9.056703	Up	RAS like family 11 member B
A_24_P339429	KCNJ12	1.592216	1.33E-05	0.002794	8.983141	Up	potassium voltage-gated channel subfamily J member 12
A_33_P3420757	AQP4	1.278225	1.34E-05	0.002794	8.978342	Up	aquaporin 4
A_23_P159335	CD8B	0.96392	1.37E-05	0.002835	8.949807	Up	CD8b molecule
A_23_P24903	P2RY2	0.995063	1.44E-05	0.002894	8.898402	Up	purinergic receptor P2Y2
A_23_P62607	IL22RA1	1.500212	1.48E-05	0.002928	8.862964	Up	interleukin 22 receptor subunit alpha 1
A_33_P3304533	RNF207	1.223159	1.67E-05	0.003222	8.722137	Up	ring finger protein 207
A_32_P34844	ADAMTS18	1.549798	1.79E-05	0.003372	8.645032	Up	ADAM metallopeptidase with thrombospondin type 1 motif 18
A_23_P389897	NGFR	1.465734	1.8E-05	0.003372	8.640153	Up	nerve growth factor receptor
A_23_P501193	KCNJ16	1.649162	1.81E-05	0.003381	8.632189	Up	potassium voltage-gated channel subfamily J member 16
A_23_P501007	EFEMP1	0.963358	1.86E-05	0.003434	8.604817	Up	EGF containing fibulin like extracellular matrix protein 1
A_33_P3353027	CHN2	0.959558	1.92E-05	0.003523	8.566558	Up	chimerin 2
A_23_P47181	GPHA2	1.258662	1.95E-05	0.003562	8.549199	Up	glycoprotein hormone alpha 2
A_33_P3232655	GHRHR	1.32053	2.15E-05	0.003813	8.440624	Up	growth hormone releasing hormone receptor
A_24_P280333	KRT40	1.503725	2.2E-05	0.003863	8.416969	Up	keratin 40
A_32_P218355	C6orf132	1.411295	2.3E-05	0.003978	8.367301	Up	chromosome 6 open reading frame 132
A_23_P402765	NRAP	1.811676	2.31E-05	0.003978	8.362425	Up	nebulin related anchoring protein
A_33_P3250173	IQCJ	1.201705	2.32E-05	0.003978	8.357898	Up	IQ motif containing J
A_24_P726336	PHACTR2	0.937904	2.47E-05	0.0042	8.289587	Up	phosphatase and actin regulator 2
A_33_P3319880	EVC2	1.047398	2.51E-05	0.004259	8.269911	Up	EvCciliary complex subunit 2
A_33_P3355508	FOXL2	1.219617	2.54E-05	0.004275	8.257205	Up	forkhead box L2
A_24_P385732	SLC51A	1.228643	2.6E-05	0.004347	8.234886	Up	solute carrier family 51 alpha subunit
A_19_P00320141	NR2F1-AS1	1.049334	2.63E-05	0.004388	8.220529	Up	NR2F1 antisense RNA 1

A_23_P166269	FAM3B	1.658734	2.81E-05	0.004546	8.14888	Up	family with sequence similarity 3 member B
A_24_P236251	DLK1	1.084201	2.9E-05	0.004667	8.116486	Up	delta like non-canonical Notch ligand 1
A_24_P50368	BLID	0.934055	3.01E-05	0.004764	8.075302	Up	BH3-like motif containing, cell death inducer
A_23_P43197	CALB1	1.816152	3.01E-05	0.004764	8.074442	Up	calbindin 1
A_33_P3346766	UGT2A1	1.919609	3.08E-05	0.004817	8.050798	Up	UDP glucuronosyltransferase family 2 member A1 complex locus
A_19_P00322810	#N/A	0.976388	3.19E-05	0.004888	8.012101	Up	NA
A_33_P3279590	OGN	1.376373	3.24E-05	0.004921	7.998292	Up	osteoglycin
A_23_P212508	TF	1.475208	3.38E-05	0.005067	7.952258	Up	transferrin
A_24_P655849	SMAD9	1.578849	3.42E-05	0.00508	7.939515	Up	SMAD family member 9
A_19_P00801466	LINC00598	1.475444	3.45E-05	0.00508	7.930302	Up	long intergenic non-protein coding RNA 598
A_23_P401055	SOX2	1.69957	3.47E-05	0.005086	7.925451	Up	SRY-box 2
A_23_P500010	KLK12	2.06498	3.51E-05	0.005129	7.913013	Up	kallikrein related peptidase 12
A_24_P882732	KRT17P1	1.106234	3.67E-05	0.005299	7.864481	Up	keratin 17 pseudogene 1
A_23_P170649	SBSPON	1.314417	3.84E-05	0.005496	7.817453	Up	somatomedin B and thrombospondin type 1 domain containing
A_33_P3345534	KRT14	1.187608	3.91E-05	0.00555	7.798667	Up	keratin 14
A_32_P930375	DLGAP2	1.302276	3.98E-05	0.005631	7.780067	Up	DLG associated protein 2
A_23_P110319	CWH43	1.018626	4.1E-05	0.005709	7.750289	Up	cell wall biogenesis 43 C-terminal homolog
A_23_P334798	LRRC2	0.953096	4.18E-05	0.005713	7.731307	Up	leucine rich repeat containing 2
A_33_P3347397	AMOT	1.359913	4.28E-05	0.005822	7.705274	Up	angiomotin
A_19_P00318158	#N/A	1.004498	4.39E-05	0.005857	7.67955	Up	NA
A_33_P3340981	LINC00598	1.256571	4.45E-05	0.005905	7.666631	Up	long intergenic non-protein coding RNA 598
A_33_P3340580	ZNF385C	0.953391	4.66E-05	0.006119	7.618782	Up	zinc finger protein 385C
A_23_P425681	CCK	1.622521	4.79E-05	0.006272	7.590454	Up	cholecystokinin
A_23_P63209	HSD11B1	1.715094	4.83E-05	0.006272	7.583273	Up	hydroxysteroid 11-beta dehydrogenase 1

A_23_P9711	HS3ST4	1.269685	4.84E-05	0.006272	7.581112	Up	heparansulfate-glucosamine 3-sulfotransferase 4
A_24_P691826	WFDC21P	0.992875	5.11E-05	0.00653	7.524302	Up	WAP four-disulfide core domain 21, pseudogene
A_19_P00807452	#N/A	1.104671	5.13E-05	0.00653	7.520302	Up	NA
A_33_P3267375	SGCD	1.257873	5.28E-05	0.006685	7.492188	Up	sarcoglycan delta
A_33_P3329088	PRSS8	1.069842	5.57E-05	0.00695	7.439292	Up	protease, serine 8
A_33_P3249229	OR3A4P	1.184305	5.63E-05	0.006981	7.427986	Up	olfactory receptor family 3 subfamily A member 4 pseudogene
A_32_P85106	C6orf118	1.123262	5.67E-05	0.007016	7.420158	Up	chromosome 6 open reading frame 118
A_19_P00804546	MIR503HG	2.046985	6.03E-05	0.007246	7.359333	Up	MIR503 host gene
A_33_P3303810	LAD1	1.154517	6.1E-05	0.007305	7.348491	Up	ladinin 1
A_33_P3369098	MYL10	1.181726	6.32E-05	0.007474	7.311978	Up	myosin light chain 10
A_32_P173662	CRISP2	1.011029	6.4E-05	0.007486	7.299977	Up	cysteine rich secretory protein 2
A_33_P3338698	IHH	1.292631	6.61E-05	0.007644	7.268026	Up	indian hedgehog
A_24_P395814	CGB3	1.492903	6.68E-05	0.007644	7.257466	Up	chorionic gonadotropin beta subunit 3
A_33_P3307475	AC129507.4	0.949086	7.03E-05	0.007924	7.207806	Up	NA
A_23_P23639	MCOLN2	0.940979	7.22E-05	0.008078	7.182496	Up	mucolipin 2
A_32_P206839	LOC100288911	1.265982	7.31E-05	0.008125	7.169161	Up	uncharacterized LOC100288911
A_32_P232214	TRABD2B	1.388547	7.41E-05	0.008172	7.156892	Up	TraB domain containing 2B
A_23_P24149	SLC39A12	1.443756	7.43E-05	0.008172	7.153512	Up	solute carrier family 39 member 12
A_23_P56578	VIT	1.322911	7.57E-05	0.0083	7.135859	Up	vitrin
A_23_P83298	PRRX2	1.098103	7.63E-05	0.008328	7.128004	Up	paired related homeobox 2
A_23_P145724	PPP1R17	1.564573	7.66E-05	0.008336	7.124207	Up	protein phosphatase 1 regulatory subunit 17
A_23_P12128	TSHB	1.120155	7.89E-05	0.0085	7.095106	Up	thyroid stimulating hormone beta
A_23_P53763	RUBCNL	1.059222	8.04E-05	0.008513	7.077398	Up	RUN and cysteine rich domain containing beclin 1 interacting protein like
A_23_P31407	AGR2	1.36206	8.09E-05	0.008524	7.071021	Up	anterior gradient 2, protein disulphide isomerase family member

A_32_P88240	KBTBD12	1.043668	8.24E-05	0.008575	7.053992	Up	kelch repeat and BTB domain containing 12
A_33_P3294317	LINC02182	1.330276	8.24E-05	0.008575	7.053473	Up	long intergenic non-protein coding RNA 2182
A_24_P120934	GADD45G	1.025983	8.38E-05	0.008626	7.037193	Up	growth arrest and DNA damage inducible gamma
A_33_P3228460	FXYD3	0.948671	8.46E-05	0.008651	7.028524	Up	FXYD domain containing ion transport regulator 3
A_32_P116606	LIX1	1.396555	8.51E-05	0.008656	7.022811	Up	limb and CNS expressed 1
A_33_P3223898	PRSS45	1.185061	8.8E-05	0.008806	6.991076	Up	protease, serine 45
A_23_P164057	MFAP4	1.358535	8.97E-05	0.008914	6.972708	Up	microfibrillar associated protein 4
A_23_P36531	TSPAN8	1.284499	9.06E-05	0.008981	6.963243	Up	tetraspanin 8
A_24_P170983	ESPNL	0.967629	9.17E-05	0.009027	6.951781	Up	espin-like
A_33_P3330791	LOC401220	1.563068	9.25E-05	0.009087	6.94327	Up	uncharacterized LOC401220
A_32_P133072	SPON1	1.725709	9.31E-05	0.009105	6.936563	Up	spondin 1
A_33_P3884230	NFIX	0.941992	9.37E-05	0.009105	6.930503	Up	nuclear factor I X
A_33_P3275315	CELF2-AS1	0.940083	9.96E-05	0.009568	6.87276	Up	CELF2 antisense RNA 1
A_23_P320225	EVC2	0.975087	0.000102	0.009726	6.850414	Up	EvCciliary complex subunit 2
A_24_P365975	COL8A2	1.028966	0.000103	0.009729	6.840562	Up	collagen type VIII alpha 2 chain
A_33_P3419006	GNRHR	1.703721	0.000105	0.009848	6.820505	Up	gonadotropin releasing hormone receptor
A_23_P51002	SULT1C2	1.23956	0.000106	0.009854	6.817742	Up	sulfotransferase family 1C member 2
A_19_P00321190	LINC01606	1.082379	0.000109	0.010008	6.788943	Up	long intergenic non-protein coding RNA 1606
A_23_P83838	CA8	1.224183	0.00011	0.010051	6.778619	Up	carbonic anhydrase 8
A_24_P168925	CHRDL1	1.561708	0.000111	0.010065	6.77067	Up	chordin like 1
A_24_P926960	MEGF6	1.23387	0.000116	0.010291	6.733096	Up	multiple EGF like domains 6
A_23_P5370	RPRM	1.644262	0.000118	0.010371	6.717833	Up	reprimo, TP53 dependent G2 arrest mediator homolog
A_23_P129133	OCA2	1.181096	0.000118	0.010371	6.714341	Up	OCA2 melanosomaltransmembrane protein
A_23_P146855	MPPED1	1.224199	0.000119	0.010437	6.706512	Up	metallophosphoesterase domain containing 1

A_23_P167493	CARTPT	1.348534	0.000123	0.010605	6.676755	Up	CART prepropeptide
A_23_P381351	CSH1	1.395394	0.000123	0.010605	6.676545	Up	chorionic somatomammotropin hormone 1
A_23_P252462	GALNT9	0.972962	0.000126	0.01073	6.651804	Up	polypeptide N-acetylgalactosaminyltransferase 9
A_23_P82990	OGN	1.458835	0.000129	0.010881	6.628807	Up	osteoglycin
A_32_P84369	FAM153B	1.100172	0.000131	0.010897	6.618167	Up	family with sequence similarity 153 member B
A_33_P3252394	GADD45G	1.041451	0.000132	0.010919	6.612967	Up	growth arrest and DNA damage inducible gamma
A_23_P209978	VSNL1	1.042135	0.000134	0.011037	6.599626	Up	visinin like 1
A_33_P3231357	KISS1R	1.447717	0.000134	0.01105	6.596694	Up	KISS1 receptor
A_23_P212830	FGFR3	1.121697	0.000135	0.011086	6.586685	Up	fibroblast growth factor receptor 3
A_33_P3680789	PROSER2-AS1	1.087472	0.000137	0.011165	6.575783	Up	PROSER2 antisense RNA 1
A_33_P3341499	WNT5A	1.252562	0.000137	0.011165	6.574844	Up	Wnt family member 5A
A_23_P357881	CD8B	1.210532	0.000138	0.011213	6.569217	Up	CD8b molecule
A_23_P81103	SFRP2	1.416148	0.000152	0.011906	6.483851	Up	secreted frizzled related protein 2
A_23_P92730	HSPB3	1.413693	0.000162	0.012387	6.426669	Up	heat shock protein family B (small) member 3
A_33_P3306267	PAX6	1.112687	0.000163	0.012452	6.416123	Up	paired box 6
A_24_P345451	CYBRD1	1.038518	0.000166	0.01256	6.402632	Up	cytochrome b reductase 1
A_33_P3228450	FXYD3	1.266019	0.000169	0.012624	6.387741	Up	FXYD domain containing ion transport regulator 3
A_33_P3480395	FLJ30901	1.022724	0.000172	0.012732	6.371786	Up	uncharacterized protein FLJ30901
A_23_P138938	PGR	1.816923	0.000175	0.012856	6.35462	Up	progesterone receptor
A_19_P00803588	#N/A	1.00275	0.000176	0.012892	6.349834	Up	NA
A_23_P118065	HSD17B2	1.040626	0.000183	0.013153	6.316724	Up	hydroxysteroid 17-beta dehydrogenase 2
A_32_P160563	OPCML	1.365608	0.000185	0.01326	6.308043	Up	opioid binding protein/cell adhesion molecule like
A_23_P317760	FAM217A	0.982831	0.000189	0.013446	6.284687	Up	family with sequence similarity 217 member A
A_23_P76332	RFX4	1.06413	0.000193	0.013638	6.266747	Up	regulatory factor X4

A_23_P42811	AGR3	1.186616	0.000196	0.013804	6.253129	Up	anterior gradient 3, protein disulphide isomerase family member
A_24_P940644	SLIT1	1.673787	0.000201	0.013821	6.233263	Up	slit guidance ligand 1
A_23_P26847	SOX9	1.113591	0.000204	0.01395	6.220577	Up	SRY-box 9
A_33_P3351681	DEUP1	1.483472	0.00021	0.014139	6.195544	Up	deuterosome assembly protein 1
A_23_P344408	CSHL1	1.2167	0.000215	0.014376	6.173456	Up	chorionic somatomammotropin hormone like 1
A_32_P111639	CHST9	1.181236	0.000222	0.014537	6.146767	Up	carbohydrate sulfotransferase 9
A_33_P3248405	NRK	1.148294	0.000223	0.014561	6.141927	Up	Nik related kinase
A_33_P3245238	SULT1C2	1.01799	0.000226	0.014718	6.130746	Up	sulfotransferase family 1C member 2
A_23_P75063	DYDC2	1.077759	0.00023	0.014886	6.113345	Up	DPY30 domain containing 2
A_33_P3410121	CFAP74	1.034589	0.000234	0.015035	6.098098	Up	cilia and flagella associated protein 74
A_23_P39605	SULT1C4	1.072548	0.000236	0.015056	6.090655	Up	sulfotransferase family 1C member 4
A_23_P251132	SNTG2	1.884616	0.000237	0.015081	6.086334	Up	syntrophin gamma 2
A_33_P3423420	ZNF750	1.064614	0.000239	0.015087	6.082126	Up	zinc finger protein 750
A_33_P3258046	TAC4	1.141906	0.000242	0.015181	6.071276	Up	tachykinin 4 (hemokinin)
A_23_P136116	AGMO	1.074353	0.000242	0.015181	6.070326	Up	alkylglycerolmonooxygenase
A_33_P3317253	PTER	1.057567	0.000247	0.015377	6.050692	Up	phosphotriesterase related
A_23_P431139	CTRB1	1.081155	0.000249	0.015428	6.043694	Up	chymotrypsinogen B1
A_23_P18282	DLEC1	1.106803	0.000255	0.015611	6.024776	Up	deleted in lung and esophageal cancer 1
A_23_P31124	COL21A1	1.493919	0.000258	0.015701	6.015768	Up	collagen type XXI alpha 1 chain
A_19_P00320685	LINC01852	0.977693	0.000258	0.015701	6.013804	Up	long intergenic non-protein coding RNA 1852
A_19_P00805833	ATF7IP2	1.079212	0.000259	0.015703	6.010456	Up	activating transcription factor 7 interacting protein 2
A_33_P3249534	NEFM	1.748709	0.000266	0.015898	5.989561	Up	neurofilament medium
A_19_P00316370	AC002480.2	1.245626	0.000277	0.016233	5.953808	Up	NA
A_23_P124619	S100A14	1.025455	0.000278	0.016296	5.94929	Up	S100 calcium binding protein A14

A_33_P3354414	AOX1	1.169692	0.000283	0.016493	5.934365	Up	aldehyde oxidase 1
A_32_P158966	KLRF1	0.936194	0.000285	0.016572	5.92911	Up	killer cell lectin like receptor F1
A_33_P3352906	KCNIP4-IT1	1.113185	0.000289	0.016663	5.916334	Up	KCNIP4 intronic transcript 1
A_33_P3420224	ENTPD8	0.994821	0.000296	0.016851	5.896821	Up	ectonucleoside triphosphate diphosphohydrolase 8
A_23_P363255	CCDC68	1.63892	0.000298	0.016851	5.892889	Up	coiled-coil domain containing 68
A_33_P3297562	IRX2	0.941375	0.000298	0.016851	5.89031	Up	iroquoishomeobox 2
A_33_P3368646	CNKSR3	1.102873	0.000303	0.016944	5.878301	Up	CNKSR family member 3
A_23_P77731	CRYM	0.956236	0.000309	0.017112	5.860011	Up	crystallin mu
A_33_P3298810	FFAR3	1.175134	0.000311	0.017186	5.855259	Up	free fatty acid receptor 3
A_33_P3423874	MYZAP	1.382209	0.000315	0.017267	5.845554	Up	myocardial zonulaadherens protein
A_23_P81590	PDE6A	1.325565	0.000317	0.017267	5.840506	Up	phosphodiesterase 6A
A_23_P118615	ABCA8	1.154317	0.000335	0.017778	5.793871	Up	ATP binding cassette subfamily A member 8
A_19_P00317324	#N/A	1.111694	0.000336	0.017778	5.791414	Up	NA
A_23_P122924	INHBA	0.971104	0.000336	0.017778	5.790115	Up	inhibin beta A subunit
A_33_P3260125	LDLRAD2	1.408377	0.000337	0.017778	5.78717	Up	low density lipoprotein receptor class A domain containing 2
A_33_P3281667	CNTNAP3	0.947904	0.000339	0.017833	5.781984	Up	contactin associated protein-like 3
A_32_P84373	FAM153C	1.604174	0.000353	0.018266	5.74907	Up	family with sequence similarity 153, member C, pseudogene
A_33_P3847514	C6orf141	1.000679	0.000353	0.018268	5.74794	Up	chromosome 6 open reading frame 141
A_24_P34155	RUNX1	1.245442	0.00036	0.01843	5.732384	Up	runt related transcription factor 1
A_33_P3354429	TRIM71	0.994075	0.000367	0.018648	5.715808	Up	tripartite motif containing 71
A_23_P100642	PNMT	1.853774	0.000367	0.018648	5.715693	Up	phenylethanolamine N-methyltransferase
A_23_P10172	PRSS50	1.758832	0.00037	0.01872	5.708555	Up	protease, serine 50
A_24_P142503	SLC47A1	1.008499	0.000377	0.01889	5.69512	Up	solute carrier family 47 member 1
A_33_P3289371	PHACTR3	1.093945	0.000378	0.018958	5.690962	Up	phosphatase and actin regulator 3

A_33_P3421118	IL20RA	1.257448	0.000386	0.019195	5.675376	Up	interleukin 20 receptor subunit alpha
A_23_P137573	LEFTY2	1.262989	0.000389	0.019237	5.667495	Up	left-right determination factor 2
A_23_P113777	ITGBL1	1.325051	0.000395	0.019372	5.654497	Up	integrin subunit beta like 1
A_33_P3274084	RHBDL3	1.138528	0.000408	0.019769	5.628682	Up	rhomboid like 3
A_23_P207194	GH1	1.18446	0.000413	0.019911	5.618101	Up	growth hormone 1
A_24_P943781	CPED1	1.074957	0.000414	0.019933	5.616267	Up	cadherin like and PC-esterase domain containing 1
A_33_P3307500	STRA6	1.085733	0.000416	0.020011	5.612103	Up	stimulated by retinoic acid 6
A_33_P3380642	FRAS1	1.087367	0.000418	0.020041	5.608323	Up	Fraser extracellular matrix complex subunit 1
A_33_P3394833	FOXL2NB	1.814458	0.000418	0.020041	5.608183	Up	FOXL2 neighbor
A_32_P780817	CT45A1	1.206224	0.000429	0.020315	5.586514	Up	cancer/testis antigen family 45 member A1
A_24_P51375	#N/A	1.182894	0.000432	0.020315	5.58114	Up	NA
A_33_P3289121	C2orf40	1.024588	0.000433	0.020315	5.579578	Up	chromosome 2 open reading frame 40
A_23_P500501	FGFR3	1.024692	0.000434	0.020315	5.578659	Up	fibroblast growth factor receptor 3
A_33_P3252281	EYA4	1.461336	0.00044	0.02041	5.566737	Up	EYA transcriptional coactivator and phosphatase 4
A_33_P3415247	TNFRSF14-AS1	1.033693	0.000443	0.020438	5.560127	Up	TNFRSF14 antisense RNA 1
A_33_P3268783	AMZ1	1.291381	0.000444	0.020438	5.558504	Up	archaelysin family metallopeptidase 1
A_19_P00316453	AL033384.1	1.046682	0.000446	0.020479	5.556002	Up	NA
A_33_P3275846	CACNA1A	1.199152	0.000464	0.020678	5.524021	Up	calcium voltage-gated channel subunit alpha1 A
A_32_P202013	FAM196A	1.104724	0.000465	0.020688	5.521658	Up	family with sequence similarity 196 member A
A_24_P330518	CA12	1.139863	0.000469	0.020745	5.51508	Up	carbonic anhydrase 12
A_24_P82466	GAS7	1.085699	0.00047	0.020745	5.513526	Up	growth arrest specific 7
A_24_P71904	HPGD	1.396742	0.000472	0.020815	5.509054	Up	hydroxyprostaglandin dehydrogenase 15-(NAD)
A_19_P00316914	AL160408.4	1.192461	0.00048	0.020964	5.494857	Up	NA
A_23_P233	FMO5	1.160289	0.000489	0.02119	5.480019	Up	flavin containing monooxygenase 5

A_23_P150555	SCGB1D2	1.294842	0.000493	0.021282	5.473241	Up	secretoglobin family 1D member 2
A_23_P39251	PLIN5	1.34129	0.000494	0.021282	5.472734	Up	perilipin 5
A_24_P498652	KCNA1	1.463762	0.000499	0.021368	5.463722	Up	potassium voltage-gated channel subfamily A member 1
A_32_P169179	MSX2P1	1.197032	0.000514	0.021729	5.440466	Up	mshhomeobox 2 pseudogene 1
A_23_P415974	ALDOB	1.370957	0.000517	0.021761	5.436053	Up	aldolase, fructose-bisphosphate B
A_23_P1523	RHOD	1.601066	0.000523	0.021814	5.426643	Up	ras homolog family member D
A_33_P3273777	GJB7	1.405572	0.000524	0.021814	5.42471	Up	gap junction protein beta 7
A_23_P118894	PRR15L	0.938926	0.000529	0.021921	5.417484	Up	proline rich 15 like
A_23_P156025	IRX2	1.76183	0.000537	0.022073	5.404917	Up	iroquoishomeobox 2
A_23_P205959	ALDH1A3	1.435514	0.000545	0.022276	5.393236	Up	aldehyde dehydrogenase 1 family member A3
A_23_P319859	EYA2	1.179968	0.000547	0.022323	5.389659	Up	EYA transcriptional coactivator and phosphatase 2
A_23_P66798	KRT19	0.946886	0.000555	0.022454	5.378419	Up	keratin 19
A_19_P00800509	#N/A	1.027445	0.000556	0.022454	5.37672	Up	NA
A_23_P134914	LY6H	1.829315	0.000559	0.022496	5.373259	Up	lymphocyte antigen 6 family member H
A_33_P3358750	MYBPHL	1.079067	0.000559	0.022496	5.372754	Up	myosin binding protein H like
A_23_P124642	RASGRP1	0.942131	0.000563	0.022525	5.367106	Up	RAS guanyl releasing protein 1
A_24_P216014	GH2	2.09163	0.000572	0.022752	5.353925	Up	growth hormone 2
A_23_P304489	FAM19A5	1.339382	0.000575	0.022762	5.350333	Up	family with sequence similarity 19 member A5, C-C motif chemokine like
A_23_P131935	FERMT1	1.051911	0.000587	0.023011	5.333832	Up	fermitin family member 1
A_33_P3339138	RPRML	0.963599	0.000597	0.023299	5.319449	Up	reprimo like
A_33_P3533325	ASXL3	1.030682	0.000602	0.023401	5.313045	Up	additional sex combs like 3, transcriptional regulator
A_19_P00801490	LINC02562	1.317451	0.000608	0.023525	5.305686	Up	long intergenic non-protein coding RNA 2562
A_33_P3415888	TEPP	1.140263	0.000613	0.023662	5.298433	Up	testis, prostate and placenta expressed
A_23_P21618	PDZRN3	1.396019	0.000615	0.023699	5.296458	Up	PDZ domain containing ring finger 3

A_33_P3340199	AC096577.1	0.958785	0.000623	0.023872	5.286379	Up	NA
A_24_P46911	KCNS2	1.033156	0.000632	0.024042	5.275064	Up	potassium voltage-gated channel modifier subfamily S member 2
A_24_P561341	ALKAL2	1.48007	0.000637	0.02411	5.268559	Up	ALK and LTK ligand 2
A_33_P3282005	VANGL2	1.487347	0.000645	0.024339	5.25835	Up	VANGL planar cell polarity protein 2
A_33_P3277890	WASIR1	0.992418	0.000648	0.024395	5.255051	Up	WASH and IL9R antisense RNA 1
A_33_P3353471	GRIK1-AS1	0.971052	0.000654	0.02451	5.247831	Up	GRIK1 antisense RNA 1
A_32_P225816	PRDM16	1.315384	0.000657	0.024583	5.244556	Up	PR/SET domain 16
A_23_P148609	PLAC1	0.962204	0.000658	0.024583	5.242831	Up	placenta specific 1
A_33_P3271121	SH3BP4	1.281724	0.00066	0.024583	5.240632	Up	SH3 domain binding protein 4
A_23_P217379	COL4A6	1.479885	0.00066	0.024583	5.239941	Up	collagen type IV alpha 6 chain
A_33_P3285965	NEUROD1	1.700584	0.000662	0.024605	5.238558	Up	neuronal differentiation 1
A_33_P3288844	IL6R	1.168827	0.000663	0.02462	5.236673	Up	interleukin 6 receptor
A_23_P312271	TEX26	1.26024	0.000663	0.02462	5.236465	Up	testis expressed 26
A_33_P3298326	FSHB	2.112742	0.000664	0.02462	5.235787	Up	follicle stimulating hormone beta subunit
A_23_P207154	CSH2	1.34608	0.000664	0.02462	5.235296	Up	chorionic somatomammotropin hormone 2
A_33_P3321382	CNTNAP3B	1.057166	0.000677	0.024843	5.220864	Up	contactin associated protein like 3B
A_23_P159893	CHRDL1	1.077118	0.000677	0.024843	5.220658	Up	chordin like 1
A_33_P3519250	NWD1	1.146885	0.000687	0.025088	5.208883	Up	NACHT and WD repeat domain containing 1
A_33_P3333158	CPXM2	1.235098	0.000698	0.025302	5.196075	Up	carboxypeptidase X, M14 family member 2
A_33_P3283122	WWC2	0.99086	0.000716	0.025673	5.176277	Up	WW and C2 domain containing 2
A_24_P254506	PAGE4	1.146619	0.000716	0.025673	5.176022	Up	PAGE family member 4
A_23_P136777	APOD	1.229853	0.000717	0.025673	5.174762	Up	apolipoprotein D
A_23_P30983	GJB7	1.662038	0.000739	0.026039	5.151262	Up	gap junction protein beta 7
A_33_P3273490	TRBV20OR9-2	0.942031	0.000755	0.026226	5.134727	Up	T-cell receptor beta variable 20/OR9-2 (non-functional)

A_23_P23292	RXRG	1.363857	0.000764	0.026479	5.125969	Up	retinoid X receptor gamma
A_23_P340263	RNF175	1.370623	0.000772	0.026711	5.117906	Up	ring finger protein 175
A_23_P398189	IGSF11	1.162188	0.000776	0.026797	5.113508	Up	immunoglobulin superfamily member 11
A_32_P187571	SCN2B	1.633253	0.000786	0.026893	5.103165	Up	sodium voltage-gated channel beta subunit 2
A_23_P125680	TMEM255A	1.375195	0.000791	0.026979	5.098126	Up	transmembrane protein 255A
A_23_P143348	OVOL2	1.504252	0.000792	0.026979	5.097858	Up	ovo like zinc finger 2
A_32_P60716	CCDC158	1.039562	0.000793	0.026979	5.096324	Up	coiled-coil domain containing 158
A_32_P141418	ARMC4	1.39728	0.0008	0.027047	5.090047	Up	armadillo repeat containing 4
A_33_P3236676	C9orf152	1.121102	0.000831	0.02766	5.060268	Up	chromosome 9 open reading frame 152
A_23_P381746	ASXL3	1.378008	0.00086	0.028195	5.033829	Up	additional sex combs like 3, transcriptional regulator
A_23_P114713	CYP4B1	1.134734	0.000878	0.02848	5.018238	Up	cytochrome P450 family 4 subfamily B member 1
A_23_P392317	DLGAP2	1.280103	0.000879	0.028513	5.016681	Up	DLG associated protein 2
A_33_P3320197	ALKAL2	1.220161	0.000889	0.028637	5.008349	Up	ALK and LTK ligand 2
A_23_P110462	PCDHB1	0.960895	0.000913	0.029158	4.987739	Up	protocadherin beta 1
A_23_P150595	TPH1	0.948973	0.000924	0.029301	4.978952	Up	tryptophan hydroxylase 1
A_23_P154037	AOX1	1.062065	0.000944	0.029642	4.96208	Up	aldehyde oxidase 1
A_23_P86470	CH25H	0.971876	0.000966	0.030028	4.944873	Up	cholesterol 25-hydroxylase
A_33_P3283824	SLC39A8	1.133024	0.00098	0.03038	4.93375	Up	solute carrier family 39 member 8
A_23_P143526	S100B	1.144173	0.000999	0.030653	4.91915	Up	S100 calcium binding protein B
A_33_P3292646	MALRD1	1.208959	0.001004	0.0307	4.915254	Up	MAM and LDL receptor class A domain containing 1
A_32_P34826	B3GALT5-AS1	1.541162	0.001013	0.030874	4.908374	Up	B3GALT5 antisense RNA 1
A_33_P3319022	CLEC9A	0.985478	0.001014	0.030874	4.907676	Up	C-type lectin domain containing 9A
A_24_P190472	SLPI	1.126312	0.001023	0.031057	4.900711	Up	secretory leukocyte peptidase inhibitor
A_32_P49199	PGR	1.356832	0.001051	0.031385	4.880628	Up	progesterone receptor

A_33_P3352467	SSTR2	1.022938	0.001051	0.031385	4.880106	Up	somatostatin receptor 2
A_19_P00321962	AP000473.1	1.1851	0.001063	0.031601	4.871734	Up	NA
A_24_P236235	FLRT2	0.96055	0.001102	0.032397	4.844468	Up	fibronectinleucine rich transmembrane protein 2
A_33_P3232498	C2orf71	0.973752	0.001115	0.032517	4.835307	Up	chromosome 2 open reading frame 71
A_23_P1331	COL13A1	1.167848	0.001139	0.032752	4.819329	Up	collagen type XIII alpha 1 chain
A_33_P3373259	CACNA2D3	1.461394	0.001151	0.03292	4.811744	Up	calcium voltage-gated channel auxiliary subunit alpha2delta 3
A_23_P73632	NR0B1	1.455678	0.001157	0.03292	4.807777	Up	nuclear receptor subfamily 0 group B member 1
A_23_P21092	CALB2	1.452992	0.001175	0.033199	4.796121	Up	calbindin 2
A_23_P146274	STMN2	1.55252	0.001192	0.033466	4.785019	Up	stathmin 2
A_23_P127781	SCGB1D1	1.242168	0.001193	0.033466	4.784787	Up	secretoglobin family 1D member 1
A_23_P213336	FGF1	0.986971	0.001205	0.033585	4.777304	Up	fibroblast growth factor 1
A_23_P48229	KCNA1	1.237757	0.001218	0.03383	4.769327	Up	potassium voltage-gated channel subfamily A member 1
A_23_P110052	FOXL2	1.411075	0.001265	0.0345	4.740819	Up	forkhead box L2
A_23_P308483	MAP3K15	1.119098	0.001276	0.034639	4.734086	Up	mitogen-activated protein kinase kinasekinase 15
A_33_P3304082	AC100872.1	1.03319	0.001277	0.034639	4.733848	Up	NA
A_33_P3227269	GATA3-AS1	1.53053	0.001296	0.034887	4.722827	Up	GATA3 antisense RNA 1
A_24_P326491	MKX	1.009479	0.001298	0.034889	4.721501	Up	mohawkhomeobox
A_23_P434398	TXLNB	1.116641	0.001346	0.035471	4.694487	Up	taxilin beta
A_33_P3709150	T	0.957229	0.001354	0.035473	4.690142	Up	T brachyury transcription factor
A_33_P3527317	LOC148638	1.189409	0.001354	0.035473	4.689917	Up	uncharacterized LOC148638
A_32_P183765	ERBB4	1.80022	0.001409	0.036262	4.66067	Up	erb-b2 receptor tyrosine kinase 4
A_19_P00811193	#N/A	0.967772	0.00141	0.036262	4.660162	Up	NA
A_23_P129835	PPP1R1B	1.133206	0.001419	0.036362	4.655157	Up	protein phosphatase 1 regulatory inhibitor subunit 1B
A_23_P79968	PCSK2	1.403328	0.001437	0.036661	4.645726	Up	proproteinconvertasesubtilisin/kexin type 2

A_33_P3405459	FNDC11	1.20722	0.001446	0.036818	4.641233	Up	fibronectin type III domain containing 11
A_23_P320261	DMKN	0.95874	0.001475	0.037025	4.626565	Up	dermokine
A_23_P200780	TGFBR3	1.016642	0.00148	0.037043	4.623865	Up	transforming growth factor beta receptor 3
A_23_P6381	MN1	0.940638	0.001497	0.037402	4.615481	Up	MN1 proto-oncogene, transcriptional regulator
A_33_P3411035	SLIT1	1.425281	0.001504	0.037429	4.612055	Up	slit guidance ligand 1
A_23_P210581	KCNG1	0.947554	0.001562	0.038407	4.584504	Up	potassium voltage-gated channel modifier subfamily G member 1
A_23_P54340	MEGF11	1.058389	0.001582	0.038509	4.575181	Up	multiple EGF like domains 11
A_23_P73571	MUM1L1	1.588137	0.001589	0.038563	4.571829	Up	MUM1 like 1
A_33_P3846653	KRT19P2	1.050094	0.001666	0.039613	4.537271	Up	keratin 19 pseudogene 2
A_23_P11685	PLA2G4A	0.933455	0.001677	0.039754	4.532537	Up	phospholipase A2 group IVA
A_23_P139687	ERP27	0.966953	0.001688	0.039785	4.52741	Up	endoplasmic reticulum protein 27
A_33_P3351279	GPR37L1	0.966182	0.001697	0.039868	4.523641	Up	G protein-coupled receptor 37 like 1
A_33_P3311503	SMR3B	0.956584	0.001702	0.039925	4.521576	Up	submaxillary gland androgen regulated protein 3B
A_33_P3336686	CLIC3	0.9489	0.001714	0.04004	4.51625	Up	chloride intracellular channel 3
A_23_P421306	SYT12	1.356431	0.001721	0.040069	4.513554	Up	synaptotagmin 12
A_33_P3219939	CUBN	0.988697	0.00175	0.040474	4.501436	Up	cubilin
A_32_P34046	HFM1	1.161494	0.001751	0.040474	4.500666	Up	HFM1, ATP dependent DNA helicase homolog
A_33_P3459365	LINC00880	1.060831	0.001756	0.040509	4.498854	Up	long intergenic non-protein coding RNA 880
A_23_P377094	PNMA3	1.024371	0.001846	0.041758	4.462341	Up	paraneoplastic Ma antigen 3
A_33_P3343182	IQCA1	1.174187	0.00185	0.041796	4.461004	Up	IQ motif containing with AAA domain 1
A_33_P3729375	#N/A	1.126453	0.001854	0.041826	4.459231	Up	NA
A_33_P3367126	ARHGEF28	0.948291	0.001857	0.041848	4.458083	Up	Rho guanine nucleotide exchange factor 28
A_33_P3387766	LINC00908	1.113277	0.00189	0.042112	4.445516	Up	long intergenic non-protein coding RNA 908
A_24_P187799	CPED1	0.969089	0.001898	0.042255	4.442173	Up	cadherin like and PC-esterase domain containing 1

A_33_P3392829	ESPNL	1.098479	0.001938	0.042586	4.4272	Up	espin-like
A_23_P69154	FAM198A	1.114636	0.001939	0.042586	4.426766	Up	family with sequence similarity 198 member A
A_33_P3214988	EFCAB1	1.067562	0.00196	0.042762	4.419076	Up	EF-hand calcium binding domain 1
A_23_P344568	FAM124A	1.051178	0.002042	0.043747	4.389351	Up	family with sequence similarity 124 member A
A_23_P117851	CPLX3	1.042413	0.002111	0.044804	4.365519	Up	complexin 3
A_33_P3772150	ADARB2	1.299361	0.00212	0.044804	4.362717	Up	adenosine deaminase, RNA specific B2 (inactive)
A_33_P3246883	DMKN	1.021837	0.002122	0.044804	4.361736	Up	dermokine
A_33_P3282213	#N/A	1.051166	0.002124	0.044805	4.361056	Up	NA
A_19_P00810522	#N/A	1.16815	0.002131	0.044805	4.358693	Up	NA
A_23_P10232	BANK1	1.035706	0.002181	0.045387	4.342172	Up	B-cell scaffold protein with ankyrin repeats 1
A_23_P338534	HIF3A	1.003302	0.002203	0.045626	4.335127	Up	hypoxia inducible factor 3 alpha subunit
A_23_P146294	EFCAB1	1.496561	0.00224	0.045987	4.323259	Up	EF-hand calcium binding domain 1
A_24_P929322	FLJ42393	1.329889	0.00225	0.046061	4.319918	Up	uncharacterized LOC401105
A_24_P418203	CNTNAP3	1.053068	0.002253	0.046061	4.319016	Up	contactin associated protein-like 3
A_23_P7684	CCNJL	1.325275	0.002253	0.046061	4.319	Up	cyclin J like
A_33_P3412428	ADAM32	0.990478	0.002281	0.046295	4.310264	Up	ADAM metallopeptidase domain 32
A_23_P307844	PHYHD1	1.331487	0.002319	0.046753	4.298482	Up	phytanoyl-CoA dioxygenase domain containing 1
A_33_P3220207	ARMC3	1.169263	0.002349	0.047043	4.289263	Up	armadillo repeat containing 3
A_32_P313405	LAMA1	1.220699	0.002363	0.047061	4.284963	Up	laminin subunit alpha 1
A_33_P3261408	TMIE	0.93458	0.002363	0.047061	4.284906	Up	transmembrane inner ear
A_33_P3258542	SPINK8	0.974261	0.002386	0.047221	4.278059	Up	serine peptidase inhibitor, Kazal type 8 (putative)
A_33_P3285629	DNAI2	1.079141	0.002387	0.047221	4.277946	Up	dynein axonemal intermediate chain 2
A_33_P3215729	RPS3AP14	1.011099	0.002429	0.047738	4.265334	Up	ribosomal protein S3a pseudogene 14
A_23_P64121	KIAA1549L	1.381953	0.00253	0.048635	4.236572	Up	KIAA1549 like

A_23_P80242	SEZ6L	1.445032	0.002533	0.048676	4.235657	Up	seizure related 6 homolog like
A_33_P3302453	LINC00242	1.002045	0.002592	0.049393	4.219266	Up	long intergenic non-protein coding RNA 242
A_24_P33446	KRTAP5-AS1	−1.18091	7.62E-08	9.03E-05	−17.1132	Down	KRTAP5-1/KRTAP5-2 antisense RNA 1
A_33_P3259028	TRIM24	−0.41581	1.15E-06	0.000557	−12.2547	Down	tripartite motif containing 24
A_33_P3212684	#N/A	−0.76271	1.64E-06	0.000718	−11.7307	Down	NA
A_23_P139099	OR4A15	−0.42416	2.37E-06	0.000899	−11.2005	Down	olfactory receptor family 4 subfamily A member 15
A_23_P159974	KLHL13	−0.37716	3.9E-06	0.00122	−10.5164	Down	kelch like family member 13
A_33_P3269503	KRT87P	−0.94844	4.21E-06	0.001298	−10.4139	Down	keratin 87 pseudogene
A_19_P00802970	ZRANB2-AS1	−0.56876	4.36E-06	0.001326	−10.3675	Down	ZRANB2 antisense RNA 1
A_19_P00323612	#N/A	−0.41168	4.64E-06	0.001358	−10.2869	Down	NA
A_19_P00326199	SINHCAF	−0.52736	4.72E-06	0.001358	−10.2635	Down	SIN3-HDAC complex associated factor
A_23_P254831	MAGEB2	−0.36511	5.16E-06	0.001443	−10.1497	Down	MAGE family member B2
A_19_P00327423	#N/A	−0.74048	5.89E-06	0.001597	−9.97785	Down	NA
A_19_P00800667	LINC00398	−0.31923	6.4E-06	0.00171	−9.87313	Down	long intergenic non-protein coding RNA 398
A_19_P00811704	#N/A	−0.49672	6.45E-06	0.00171	−9.86335	Down	NA
A_33_P3257910	C3orf80	−1.01528	9.53E-06	0.002277	−9.38237	Down	chromosome 3 open reading frame 80
A_19_P00315872	#N/A	−0.56281	1.14E-05	0.0026	−9.16497	Down	NA
A_23_P121596	PPBP	−1.54696	1.17E-05	0.002615	−9.13962	Down	pro-platelet basic protein
A_23_P259586	TTK	−1.24521	1.18E-05	0.002615	−9.12483	Down	TTK protein kinase
A_23_P328145	FAM71D	−0.93751	1.24E-05	0.002678	−9.06826	Down	family with sequence similarity 71 member D
A_19_P00326831	#N/A	−0.74662	1.25E-05	0.002678	−9.06195	Down	NA
A_23_P382682	NOXRED1	−0.73831	1.4E-05	0.002852	−8.92458	Down	NADP dependent oxidoreductase domain containing 1
A_33_P3235048	TRIM77	−0.40971	1.46E-05	0.002909	−8.88124	Down	tripartite motif containing 77
A_23_P118834	TOP2A	−1.2832	1.69E-05	0.003223	−8.71249	Down	topoisomerase (DNA) II alpha

A_33_P3242649	KIF18A	−1.15029	1.7E-05	0.003236	−8.70182	Down	kinesin family member 18A
A_23_P68610	TPX2	−0.6427	1.98E-05	0.003585	−8.53261	Down	TPX2, microtubule nucleation factor
A_19_P00323829	#N/A	−0.90037	2.08E-05	0.003738	−8.47651	Down	NA
A_19_P00330864	#N/A	−1.06535	2.11E-05	0.003766	−8.46369	Down	NA
A_19_P00801104	#N/A	−0.54282	2.66E-05	0.004416	−8.20936	Down	NA
A_23_P212284	POC1A	−0.3445	2.71E-05	0.004457	−8.18814	Down	POC1 centriolar protein A
A_19_P00813027	#N/A	−0.38569	2.75E-05	0.004497	−8.17297	Down	NA
A_33_P3325502	ARHGAP29	−0.93195	2.78E-05	0.004538	−8.15889	Down	Rho GTPase activating protein 29
A_23_P315815	NRG1	−0.34435	2.93E-05	0.004679	−8.10578	Down	neuregulin 1
A_33_P3335825	PLCD4	−0.74316	3.14E-05	0.004861	−8.02939	Down	phospholipase C delta 4
A_33_P3354001	CADPS2	−0.29264	3.19E-05	0.004888	−8.01287	Down	calcium dependent secretion activator 2
A_33_P3788618	ACER2	−0.29305	3.24E-05	0.004921	−7.99726	Down	alkaline ceramidase 2
A_24_P193592	CCNF	−0.96239	3.27E-05	0.004954	−7.98653	Down	cyclin F
A_33_P3576853	NIFK-AS1	−0.43661	3.3E-05	0.004986	−7.97595	Down	NIFK antisense RNA 1
A_33_P3250015	#N/A	−0.54203	3.63E-05	0.005271	−7.87712	Down	NA
A_19_P00808523	#N/A	−0.31475	3.87E-05	0.005513	−7.80907	Down	NA
A_23_P57588	GTSE1	−0.80101	4.28E-05	0.005822	−7.70706	Down	G2 and S-phase expressed 1
A_23_P366983	TRHDE	−0.85404	4.36E-05	0.005857	−7.6877	Down	thyrotropin releasing hormone degrading enzyme
A_33_P3405680	ARHGEF3-AS1	−0.50887	4.6E-05	0.006063	−7.63133	Down	ARHGEF3 antisense RNA 1
A_24_P911676	SOX4	−0.48583	4.82E-05	0.006272	−7.58518	Down	SRY-box 4
A_19_P00807368	#N/A	−0.29663	5.04E-05	0.006496	−7.53945	Down	NA
A_19_P00320026	DLEU1	−0.42962	5.06E-05	0.006498	−7.53609	Down	deleted in lymphocytic leukemia 1 (non-protein coding)
A_19_P00325235	#N/A	−0.6208	5.14E-05	0.00653	−7.51885	Down	NA
A_19_P00321005	#N/A	−0.9916	5.6E-05	0.006971	−7.43242	Down	NA

A_23_P118174	PLK1	−0.46167	5.73E-05	0.007069	−7.40971	Down	polo like kinase 1
A_33_P3271945	HAUS6	−0.41613	5.81E-05	0.007101	−7.3965	Down	HAUS augmin like complex subunit 6
A_19_P00324653	GPALPP1	−0.33064	6.19E-05	0.007399	−7.33303	Down	GPALPP motifs containing 1
A_23_P124417	BUB1	−1.18787	6.27E-05	0.007429	−7.32071	Down	BUB1 mitotic checkpoint serine/threonine kinase
A_24_P145629	SERINC2	−0.77141	6.37E-05	0.007486	−7.30502	Down	serine incorporator 2
A_33_P3230548	KIF14	−1.33322	6.4E-05	0.007486	−7.29949	Down	kinesin family member 14
A_33_P3598466	HSP90AB6P	−0.61836	6.54E-05	0.007624	−7.27875	Down	heat shock protein 90 alpha family class B member 6, pseudogene
A_24_P122746	VWA1	−0.91594	6.68E-05	0.007644	−7.25822	Down	von Willebrand factor A domain containing 1
A_33_P3421973	FRMPD3	−0.97088	6.84E-05	0.007761	−7.23466	Down	FERM and PDZ domain containing 3
A_23_P18579	PTTG2	−0.3236	7.36E-05	0.008159	−7.16262	Down	pituitary tumor-transforming 2
A_19_P00329691	RANP3	−0.44835	7.63E-05	0.008328	−7.12757	Down	RAN, member RAS oncogene family pseudogene 3
A_33_P3377529	HOXA4	−0.54254	8.02E-05	0.008513	−7.08014	Down	homeobox A4
A_33_P3318424	#N/A	−0.32148	8.12E-05	0.008532	−7.06767	Down	NA
A_24_P219114	SEL1L	−0.34542	8.34E-05	0.008626	−7.04271	Down	SEL1L ERAD E3 ligase adaptor subunit
A_33_P3350488	NUSAP1	−0.45817	8.45E-05	0.008651	−7.02997	Down	nucleolar and spindle associated protein 1
A_33_P3343720	CELA3A	−0.49673	8.54E-05	0.008656	−7.01888	Down	chymotrypsin like elastase family member 3A
A_19_P00331977	#N/A	−0.4191	8.65E-05	0.008717	−7.00758	Down	NA
A_32_P160561	DOK6	−0.68558	9.11E-05	0.008995	−6.9574	Down	docking protein 6
A_33_P3418466	AC022296.1	−0.35285	9.32E-05	0.009105	−6.93602	Down	NA
A_19_P00331084	#N/A	−0.48869	9.37E-05	0.009105	−6.93101	Down	NA
A_19_P00322169	#N/A	−0.61999	0.000103	0.009726	−6.84557	Down	NA
A_33_P3263002	TMEM262	−0.63529	0.000104	0.009737	−6.83519	Down	transmembrane protein 262
A_33_P3284345	NRG1	−0.32711	0.000106	0.009862	−6.81496	Down	neuregulin 1
A_23_P81926	PSORS1C2	−0.36148	0.000107	0.009869	−6.80951	Down	psoriasis susceptibility 1 candidate 2

A_19_P00332459	DHX15	−1.24559	0.000108	0.009961	−6.79737	Down	DEAH-box helicase 15
A_23_P35219	NEK2	−1.05848	0.000109	0.010008	−6.78932	Down	NIMA related kinase 2
A_33_P3260511	CICP24	−0.35944	0.000109	0.010009	−6.78677	Down	capicua transcriptional repressor pseudogene 24
A_33_P3403429	LRRC69	−0.4054	0.000112	0.010141	−6.76056	Down	leucine rich repeat containing 69
A_23_P36624	TAS2R7	−0.56665	0.000113	0.010184	−6.75124	Down	taste 2 receptor member 7
A_24_P314571	SPC24	−1.03137	0.000117	0.010371	−6.72365	Down	SPC24, NDC80 kinetochore complex component
A_19_P00807332	#N/A	−0.86237	0.000118	0.010371	−6.71561	Down	NA
A_19_P00807314	#N/A	−0.30162	0.000118	0.010371	−6.7145	Down	NA
A_33_P3211014	#N/A	−0.6112	0.000121	0.010554	−6.68923	Down	NA
A_33_P3354067	AC092821.2	−0.31328	0.000124	0.010658	−6.66572	Down	NA
A_19_P00801050	#N/A	−0.48588	0.000125	0.010685	−6.65847	Down	NA
A_23_P70007	HMMR	−0.49422	0.00013	0.010887	−6.62632	Down	hyaluronan mediated motility receptor
A_33_P3770642	LINC00929	−0.84694	0.00013	0.010887	−6.62288	Down	long intergenic non-protein coding RNA 929
A_19_P00317247	PTP4A1	−0.3095	0.000135	0.011079	−6.58899	Down	protein tyrosine phosphatase type IVA, member 1
A_19_P00328255	#N/A	−0.29491	0.000136	0.011131	−6.58118	Down	NA
A_23_P19369	CARMIL1	−0.377	0.000143	0.011485	−6.53694	Down	capping protein regulator and myosin 1 linker 1
A_33_P3322125	DAZAP2	−0.33412	0.000145	0.011604	−6.52583	Down	DAZ associated protein 2
A_33_P3333177	#N/A	−0.62324	0.000146	0.011665	−6.51937	Down	NA
A_23_P104651	CDCA5	−0.36513	0.000146	0.011689	−6.51579	Down	cell division cycle associated 5
A_19_P00328658	#N/A	−0.32397	0.000148	0.011752	−6.5092	Down	NA
A_19_P00319194	#N/A	−0.87023	0.000152	0.01191	−6.48015	Down	NA
A_19_P00330750	#N/A	−0.48391	0.000153	0.01191	−6.47897	Down	NA
A_19_P00800106	#N/A	−0.51725	0.000155	0.012049	−6.46641	Down	NA
A_19_P00800448	#N/A	−0.46945	0.000156	0.012123	−6.45755	Down	NA

A_33_P3209703	#N/A	−0.38403	0.000159	0.012272	−6.44165	Down	NA
A_19_P00801014	ANTXR2	−0.33022	0.000166	0.01256	−6.40275	Down	anthrax toxin receptor 2
A_19_P00327255	#N/A	−0.33261	0.000168	0.012611	−6.39321	Down	NA
A_19_P00321594	FBXL5	−0.89313	0.00017	0.012633	−6.38353	Down	F-box and leucine rich repeat protein 5
A_33_P3420704	#N/A	−0.3589	0.00017	0.012666	−6.37957	Down	NA
A_33_P3316903	RBPMS-AS1	−0.40474	0.000175	0.012856	−6.35387	Down	RBPMS antisense RNA 1
A_23_P300100	PLA2G2D	−0.52315	0.000179	0.012985	−6.33706	Down	phospholipase A2 group IID
A_33_P3295077	AC092162.1	−0.32979	0.000182	0.013153	−6.31903	Down	NA
A_33_P3375334	DAB1	−1.09252	0.000186	0.013284	−6.30295	Down	DAB1, reelin adaptor protein
A_23_P11397	TTTY13	−0.69508	0.000186	0.013284	−6.3019	Down	testis-specific transcript, Y-linked 13 (non-protein coding)
A_23_P346884	RBPJL	−0.32051	0.000195	0.013736	−6.25896	Down	recombination signal binding protein for immunoglobulin kappa J region like
A_19_P00804053	#N/A	−0.35416	0.000197	0.013821	−6.24831	Down	NA
A_19_P00322074	#N/A	−0.36755	0.0002	0.013821	−6.23712	Down	NA
A_19_P00327072	#N/A	−0.34689	0.000201	0.013821	−6.23408	Down	NA
A_33_P3386344	FANCA	−0.32132	0.000204	0.01395	−6.22023	Down	Fanconianemia complementation group A
A_33_P3340468	CENPI	−0.73725	0.000205	0.014011	−6.21277	Down	centromere protein I
A_19_P00330152	#N/A	−0.44423	0.000206	0.014017	−6.21017	Down	NA
A_19_P00800639	#N/A	−0.48611	0.000207	0.014064	−6.20523	Down	NA
A_19_P00331152	#N/A	−0.61926	0.00021	0.014139	−6.19498	Down	NA
A_19_P00811393	#N/A	−0.30534	0.000215	0.014373	−6.17502	Down	NA
A_33_P3304268	LOC643327	−0.67616	0.000216	0.014405	−6.16802	Down	uncharacterized LOC643327
A_33_P3214690	NLGN1	−0.39399	0.000219	0.014495	−6.15644	Down	neuroligin 1
A_33_P3290729	POU3F2	−1.77448	0.000219	0.014495	−6.15601	Down	POU class 3 homeobox 2
A_23_P425332	PPP4R4	−0.37974	0.000219	0.014495	−6.15533	Down	protein phosphatase 4 regulatory subunit 4

A_33_P3345469	GPR6	−0.42562	0.000221	0.014537	−6.148	Down	G protein-coupled receptor 6
A_23_P414211	PAX4	−0.32483	0.000223	0.014561	−6.14213	Down	paired box 4
A_33_P3413224	AC092755.2	−0.39132	0.000226	0.014718	−6.12784	Down	NA
A_19_P00316773	#N/A	−0.95664	0.000227	0.014718	−6.12671	Down	NA
A_33_P3248610	ASXL2	−0.31774	0.000227	0.014718	−6.12588	Down	additional sex combs like 2, transcriptional regulator
A_19_P00320549	#N/A	−0.35255	0.000231	0.014898	−6.11129	Down	NA
A_33_P3243454	IGFL3	−1.0725	0.000233	0.014996	−6.10166	Down	IGF like family member 3
A_33_P3416414	PSAT1P1	−0.62563	0.000236	0.015056	−6.09036	Down	phosphoserine aminotransferase 1 pseudogene 1
A_33_P3307144	GRIK2	−0.39339	0.000237	0.015075	−6.08797	Down	glutamate ionotropic receptor kainate type subunit 2
A_23_P60016	PTTG3P	−0.61312	0.00024	0.015106	−6.07843	Down	pituitary tumor-transforming 3, pseudogene
A_33_P3230254	NCAPG	−0.80641	0.000243	0.015185	−6.06626	Down	non-SMC condensin I complex subunit G
A_33_P3265549	SLC19A1	−0.38232	0.000244	0.015241	−6.0618	Down	solute carrier family 19 member 1
A_33_P3629151	#N/A	−0.30303	0.000252	0.015491	−6.03643	Down	NA
A_32_P64598	LINC02156	−0.97878	0.000253	0.015555	−6.03164	Down	long intergenic non-protein coding RNA 2156
A_23_P166993	EPHB1	−0.48094	0.000255	0.015611	−6.02497	Down	EPH receptor B1
A_24_P141707	INHBE	−0.87769	0.000258	0.015701	−6.01625	Down	inhibin beta E subunit
A_19_P00324607	XIST_EXON1	−0.52726	0.000258	0.015701	−6.01367	Down	X-chromosome inactivation gene exon 1
A_23_P112187	FIBCD1	−0.32728	0.000259	0.015703	−6.01152	Down	fibrinogen C domain containing 1
A_19_P00330161	RGS5	−0.31195	0.000261	0.015752	−6.00355	Down	regulator of G protein signaling 5
A_19_P00810938	#N/A	−0.47038	0.000264	0.015866	−5.99368	Down	NA
A_33_P3671729	FLJ44715	−0.66365	0.000266	0.015901	−5.98817	Down	uncharacterized LOC386671
A_23_P63032	GUCA2B	−0.45895	0.000269	0.016011	−5.97986	Down	guanylatecyclase activator 2B
A_23_P65757	CCNB2	−0.68899	0.000272	0.016127	−5.97008	Down	cyclin B2
A_33_P3221761	KMT2B	−0.33926	0.000273	0.016143	−5.96538	Down	lysine methyltransferase 2B

A_23_P342138	ADAMTSL1	−0.30204	0.000274	0.016143	−5.96357	Down	ADAMTS like 1
A_19_P00329997	#N/A	−0.77956	0.000275	0.016166	−5.95971	Down	NA
ERCC-00126_533	#N/A	−0.61974	0.00028	0.016335	−5.94611	Down	NA
A_19_P00807001	#N/A	−0.59089	0.000281	0.016399	−5.9404	Down	NA
ERCC-00014_76	#N/A	−0.58213	0.000289	0.016663	−5.9164	Down	NA
A_19_P00330883	#N/A	−0.44021	0.000294	0.016851	−5.90168	Down	NA
A_19_P00806769	LINC01122	−0.33078	0.000297	0.016851	−5.8936	Down	long intergenic non-protein coding RNA 1122
A_33_P3300152	#N/A	−0.3139	0.000297	0.016851	−5.89333	Down	NA
A_33_P3255001	LINC00691	−0.67432	0.000299	0.016851	−5.88871	Down	long intergenic non-protein coding RNA 691
A_32_P204376	ANKRD20A2	−0.82794	0.0003	0.016851	−5.88524	Down	ankyrin repeat domain 20 family member A2
A_19_P00325303	#N/A	−0.29931	0.000302	0.016931	−5.88008	Down	NA
A_24_P43810	FAM83A	−1.14146	0.000307	0.017077	−5.86726	Down	family with sequence similarity 83 member A
A_33_P3296281	#N/A	−1.00736	0.000308	0.017112	−5.86396	Down	NA
A_33_P3395733	AL049795.1	−0.52782	0.000309	0.017112	−5.86154	Down	NA
A_33_P3229552	LRFN1	−0.3534	0.000309	0.017112	−5.86091	Down	leucine rich repeat and fibronectin type III domain containing 1
A_19_P00811105	#N/A	−0.35467	0.000309	0.017112	−5.86002	Down	NA
A_23_P201022	PKLR	−0.29705	0.000315	0.017267	−5.84461	Down	pyruvate kinase, liver and RBC
A_33_P3840512	SLC25A15	−0.30272	0.000317	0.017267	−5.83945	Down	solute carrier family 25 member 15
A_33_P3389758	#N/A	−0.38846	0.000317	0.017267	−5.83831	Down	NA
A_24_P367410	GABRQ	−0.68191	0.000319	0.017276	−5.83492	Down	gamma-aminobutyric acid type A receptor theta subunit
A_19_P00324404	#N/A	−0.32077	0.000322	0.017376	−5.82646	Down	NA
A_23_P206059	PRC1	−0.3017	0.000324	0.01742	−5.82114	Down	protein regulator of cytokinesis 1
A_23_P14083	AMIGO2	−0.44866	0.000326	0.017507	−5.81479	Down	adhesion molecule with Ig like domain 2
A_24_P587443	AC025278.1	−0.61913	0.000329	0.017606	−5.80688	Down	NA

A_33_P3248028	#N/A	−0.68935	0.000333	0.017746	−5.79811	Down	NA
A_33_P3274001	SUMO2P8	−0.31129	0.000336	0.017778	−5.79043	Down	SUMO2 pseudogene 8
A_23_P57379	CDC45	−0.42634	0.000337	0.017778	−5.78764	Down	cell division cycle 45
A_23_P254733	CENPU	−0.53283	0.00034	0.017833	−5.78005	Down	centromere protein U
A_24_P9833	PRDM11	−0.38799	0.000341	0.017833	−5.77904	Down	PR/SET domain 11
A_33_P3311543	#N/A	−0.42903	0.000344	0.017933	−5.7716	Down	NA
A_33_P3216272	LOC100133920	−0.29115	0.000348	0.01814	−5.75995	Down	methylenetetrahydrofolate dehydrogenase (NADP+ dependent) 1-like pseudogene
A_19_P00802008	#N/A	−0.36753	0.000352	0.018266	−5.75094	Down	NA
A_19_P00806281	#N/A	−0.45576	0.000355	0.018351	−5.74313	Down	NA
A_19_P00800105	#N/A	−0.48555	0.000358	0.018376	−5.73826	Down	NA
A_23_P36397	CYP27B1	−0.41362	0.00037	0.018715	−5.70973	Down	cytochrome P450 family 27 subfamily B member 1
A_24_P227091	KIF11	−0.32887	0.000374	0.018869	−5.70094	Down	kinesin family member 11
A_33_P3413518	#N/A	−0.44929	0.000375	0.018883	−5.69938	Down	NA
A_33_P3337124	LL22NC03-63E9.3	−0.34339	0.000376	0.01889	−5.69708	Down	uncharacterized LOC648691
A_19_P00811963	#N/A	−0.4231	0.000381	0.019036	−5.68577	Down	NA
A_19_P00801430	#N/A	−0.33418	0.000382	0.019068	−5.68342	Down	NA
A_24_P941759	G2E3	−0.2988	0.000386	0.019195	−5.6742	Down	G2/M-phase specific E3 ubiquitin protein ligase
A_33_P3237364	RPL36P20	−0.39151	0.000388	0.019229	−5.67001	Down	ribosomal protein L36 pseudogene 20
A_33_P3374030	LOC100132197	−0.31421	0.000393	0.019342	−5.65903	Down	uncharacterized LOC100132197
A_19_P00807621	LINC01355	−0.3223	0.000403	0.019654	−5.63861	Down	long intergenic non-protein coding RNA 1355
ERCC-00085_231	#N/A	−0.8721	0.000403	0.019654	−5.63845	Down	NA
A_33_P3346643	#N/A	−0.65369	0.000403	0.019654	−5.63821	Down	NA
A_33_P3342469	OR6T1	−0.39762	0.000405	0.019687	−5.63402	Down	olfactory receptor family 6 subfamily T member 1
A_23_P218827	POLQ	−0.61577	0.000406	0.019694	−5.63275	Down	DNA polymerase theta

A_33_P3268734	COL5A1-AS1	−0.30006	0.000411	0.019894	−5.62161	Down	COL5A1 antisense RNA 1
A_23_P250136	GRIK2	−0.50891	0.000412	0.019894	−5.61973	Down	glutamate ionotropic receptor kainate type subunit 2
A_33_P3398411	RASSF3	−0.78014	0.000419	0.020052	−5.60582	Down	Ras association domain family member 3
A_19_P00324378	#N/A	−1.09872	0.000425	0.020173	−5.59533	Down	NA
A_19_P00326161	#N/A	−0.31777	0.000432	0.020315	−5.58176	Down	NA
A_32_P62997	PBK	−1.44872	0.000433	0.020315	−5.58052	Down	PDZ binding kinase
A_33_P3304452	SLC12A5-AS1	−0.71833	0.000433	0.020315	−5.57988	Down	SLC12A5 and MMP9 antisense RNA 1
A_24_P413884	CENPA	−1.04472	0.000435	0.020351	−5.57587	Down	centromere protein A
A_33_P3335576	#N/A	−0.30201	0.000436	0.020351	−5.57466	Down	NA
A_32_P470728	GSX1	−0.37056	0.000443	0.020438	−5.56016	Down	GS homeobox 1
A_19_P00804762	#N/A	−0.48298	0.000449	0.02053	−5.55088	Down	NA
A_32_P233950	DPPA5	−0.82268	0.000449	0.02053	−5.55041	Down	developmental pluripotency associated 5
A_33_P3418185	LYNX1	−0.5229	0.000457	0.02064	−5.53525	Down	Ly6/neurotoxin 1
A_33_P3360271	EVX2	−0.52194	0.000458	0.02064	−5.53386	Down	even-skipped homeobox 2
A_24_P690811	ASPDH	−0.41954	0.000463	0.020678	−5.52511	Down	aspartate dehydrogenase domain containing
A_33_P3228709	KRTAP5-7	−0.35642	0.000464	0.020678	−5.52349	Down	keratin associated protein 5–7
A_19_P00331839	#N/A	−0.29212	0.00047	0.020745	−5.51346	Down	NA
A_19_P00331107	#N/A	−0.80169	0.000474	0.020853	−5.50635	Down	NA
A_23_P88331	DLGAP5	−0.90972	0.000475	0.020853	−5.50474	Down	DLG associated protein 5
A_33_P3258392	EDN1	−0.3889	0.000475	0.020853	−5.50367	Down	endothelin 1
A_19_P00804522	#N/A	−0.57	0.000476	0.020853	−5.50304	Down	NA
A_19_P00317565	#N/A	−0.46065	0.000476	0.020853	−5.50268	Down	NA
A_19_P00802508	#N/A	−0.59414	0.000477	0.020888	−5.49991	Down	NA
A_19_P00803716	#N/A	−0.79278	0.000484	0.021054	−5.4892	Down	NA

A_19_P00320023	#N/A	−0.53921	0.000495	0.021302	−5.47115	Down	NA
A_19_P00320253	#N/A	−0.4143	0.000504	0.021479	−5.45555	Down	NA
A_19_P00315793	AL138781.1	−0.49995	0.000507	0.021538	−5.45163	Down	NA
A_19_P00320113	#N/A	−1.04798	0.000508	0.021586	−5.44901	Down	NA
A_19_P00322033	#N/A	−0.67146	0.000518	0.021761	−5.43368	Down	NA
A_19_P00332215	#N/A	−0.31011	0.00052	0.021814	−5.43076	Down	NA
A_19_P00808792	#N/A	−0.31009	0.000523	0.021814	−5.42635	Down	NA
A_19_P00804356	RNU6-485P	−0.54041	0.000525	0.021814	−5.42362	Down	RNA, U6 small nuclear 485, pseudogene
A_19_P00808315	#N/A	−0.41158	0.000532	0.021983	−5.41287	Down	NA
A_19_P00317210	#N/A	−0.53067	0.000535	0.022069	−5.40738	Down	NA
A_23_P256956	KIF20A	−0.95407	0.000536	0.022073	−5.40566	Down	kinesin family member 20A
A_19_P00325343	#N/A	−0.32421	0.000544	0.022276	−5.39456	Down	NA
A_33_P3217357	LINC01415	−0.53677	0.000545	0.022276	−5.39301	Down	long intergenic non-protein coding RNA 1415
A_23_P153183	CNKSR2	−0.3422	0.00055	0.022384	−5.38593	Down	connector enhancer of kinase suppressor of Ras 2
A_24_P918175	LOC653581	−0.5441	0.000551	0.022408	−5.3843	Down	uncharacterized LOC653581
A_33_P3259440	GOLGA6A	−0.6024	0.000555	0.022454	−5.37827	Down	golgin A6 family member A
A_19_P00322834	#N/A	−0.52403	0.000568	0.022671	−5.35948	Down	NA
A_24_P228228	B4GALT6	−0.29383	0.000573	0.022761	−5.35212	Down	beta-1,4-galactosyltransferase 6
A_33_P3299110	ARHGAP42	−0.53734	0.000576	0.022762	−5.34871	Down	Rho GTPase activating protein 42
A_32_P81676	LINC00599	−0.78125	0.000576	0.022762	−5.34835	Down	long intergenic non-protein coding RNA 599
A_19_P00321820	#N/A	−0.74238	0.000587	0.023011	−5.33401	Down	NA
A_32_P489662	KRT28	−0.76908	0.000591	0.023113	−5.32808	Down	keratin 28
A_19_P00323720	#N/A	−0.58573	0.000591	0.023113	−5.32801	Down	NA
A_24_P49896	GDPD4	−0.51369	0.0006	0.023374	−5.31609	Down	glycerophosphodiesterphosphodiesterase domain containing 4

A_19_P00317028	#N/A	−0.43091	0.0006	0.023374	−5.31543	Down	NA
A_19_P00807254	#N/A	−0.44738	0.000607	0.023523	−5.30748	Down	NA
A_33_P3397935	CALML3-AS1	−0.33578	0.000607	0.023523	−5.30746	Down	CALML3 antisense RNA 1
A_33_P3307903	CDKN3	−0.66678	0.000613	0.023662	−5.29862	Down	cyclin dependent kinase inhibitor 3
A_19_P00324976	#N/A	−0.71172	0.000617	0.023765	−5.29352	Down	NA
A_23_P253752	MTFR2	−0.604	0.000622	0.023868	−5.28721	Down	mitochondrial fission regulator 2
A_32_P890614	LOC100268168	−0.58393	0.000624	0.023883	−5.28531	Down	uncharacterized LOC100268168
A_33_P3386414	DCAF4L2	−0.56011	0.000627	0.023974	−5.28158	Down	DDB1 and CUL4 associated factor 4 like 2
A_33_P3261695	TEX22	−0.57797	0.000629	0.024007	−5.27886	Down	testis expressed 22
A_32_P812268	CALHM1	−0.71959	0.000634	0.02408	−5.27239	Down	calcium homeostasis modulator 1
A_23_P421436	ADD2	−0.42187	0.000645	0.024339	−5.25827	Down	adducin 2
A_19_P00808731	#N/A	−0.32277	0.000654	0.02451	−5.24812	Down	NA
A_33_P3422732	LOC100131180	−0.49937	0.000659	0.024583	−5.24219	Down	uncharacterized LOC100131180
A_23_P48669	CDKN3	−0.49224	0.00066	0.024583	−5.24078	Down	cyclin dependent kinase inhibitor 3
A_33_P3251727	RYR2	−0.45157	0.000667	0.024715	−5.23158	Down	ryanodine receptor 2
A_33_P3252444	#N/A	−0.59301	0.000671	0.024806	−5.22785	Down	NA
A_19_P00323152	#N/A	−0.4622	0.000672	0.024806	−5.22606	Down	NA
A_19_P00316888	#N/A	−0.47099	0.000674	0.024821	−5.22358	Down	NA
A_33_P3323979	LOC339874	−0.3826	0.000677	0.024843	−5.22	Down	uncharacterized LOC339874
A_33_P3261530	AC087491.1	−1.00413	0.000689	0.025123	−5.20636	Down	NA
A_32_P509677	C20orf203	−0.77482	0.000697	0.025302	−5.19763	Down	chromosome 20 open reading frame 203
A_19_P00316667	LINC02099	−0.33593	0.000704	0.025461	−5.18922	Down	long intergenic non-protein coding RNA 2099
A_19_P00324450	#N/A	−0.38324	0.000709	0.025533	−5.18432	Down	NA
A_33_P3407103	YTHDF1	−0.33159	0.000724	0.025806	−5.1674	Down	YTH N6-methyladenosine RNA binding protein 1

A_33_P3382648	RBMY1B	−0.46814	0.000726	0.025851	−5.16537	Down	RNA binding motif protein, Y-linked, family 1, member B
A_33_P3348328	#N/A	−0.45257	0.000727	0.02587	−5.16416	Down	NA
A_23_P151150	FOXM1	−0.63036	0.000731	0.025919	−5.16005	Down	forkhead box M1
A_19_P00811940	#N/A	−0.41876	0.000733	0.025954	−5.15836	Down	NA
A_19_P00800868	#N/A	−0.32832	0.000751	0.026166	−5.13882	Down	NA
A_23_P1083	GJA4	−0.54207	0.000752	0.026166	−5.1378	Down	gap junction protein alpha 4
A_33_P3217619	AC078923.1	−0.55897	0.000782	0.026859	−5.10793	Down	NA
A_33_P3298173	DEFA7P	−0.44812	0.000783	0.026893	−5.10632	Down	defensin alpha 7, pseudogene
A_24_P79403	PF4	−0.6126	0.000785	0.026893	−5.10463	Down	platelet factor 4
A_33_P3300142	ANKFY1	−0.30215	0.000785	0.026893	−5.10422	Down	ankyrin repeat and FYVE domain containing 1
A_23_P14035	AKAP3	−0.92292	0.000792	0.026979	−5.09727	Down	A-kinase anchoring protein 3
A_32_P168349	#N/A	−0.4856	0.000793	0.026979	−5.09635	Down	NA
A_33_P3309999	DCAF12L2	−0.32282	0.000797	0.027003	−5.09317	Down	DDB1 and CUL4 associated factor 12 like 2
A_32_P7316	BDNF	−1.4327	0.000798	0.02703	−5.09156	Down	brain derived neurotrophic factor
A_19_P00323487	#N/A	−0.33195	0.000802	0.027092	−5.08752	Down	NA
A_23_P118815	BIRC5	−0.45791	0.000808	0.027224	−5.08189	Down	baculoviral IAP repeat containing 5
A_19_P00318567	#N/A	−0.43335	0.000816	0.027426	−5.07411	Down	NA
A_33_P3385006	SLC39A5	−0.44216	0.000817	0.027426	−5.0737	Down	solute carrier family 39 member 5
A_19_P00809507	#N/A	−0.98202	0.00082	0.027463	−5.0702	Down	NA
A_19_P00321075	PVT1	−1.26911	0.000829	0.02766	−5.06179	Down	Pvt1 oncogene (non-protein coding)
A_33_P3349265	GLYATL3	−0.50093	0.000838	0.027779	−5.05349	Down	glycine-N-acyltransferase like 3
A_33_P3374443	L1CAM	−0.56749	0.000842	0.027859	−5.05006	Down	L1 cell adhesion molecule
A_33_P3314301	SV2C	−0.47211	0.000843	0.027861	−5.04938	Down	synaptic vesicle glycoprotein 2C
A_23_P80902	KIF15	−0.4448	0.000853	0.028079	−5.04014	Down	kinesin family member 15

A_23_P143994	FANCD2	−0.60919	0.000858	0.02818	−5.03582	Down	Fanconianemia complementation group D2
A_19_P00319532	LINC00963	−0.30593	0.00086	0.028195	−5.03397	Down	long intergenic non-protein coding RNA 963
A_32_P186474	RACGAP1	−0.42374	0.000861	0.028195	−5.03302	Down	RacGTPase activating protein 1
A_33_P3346635	FAM20C	−0.51837	0.000867	0.028288	−5.02725	Down	FAM20C, golgi associated secretory pathway kinase
A_23_P74349	NUF2	−0.61002	0.000873	0.028388	−5.0222	Down	NUF2, NDC80 kinetochore complex component
A_19_P00321690	AL390755.1	−0.8147	0.000878	0.02848	−5.01818	Down	NA
A_19_P00324561	#N/A	−0.30458	0.000889	0.028637	−5.00807	Down	NA
A_33_P3354783	#N/A	−0.43482	0.000901	0.028915	−4.99774	Down	NA
A_33_P3220475	QSOX1	−0.34597	0.00091	0.0291	−4.99053	Down	quiescin sulfhydryl oxidase 1
A_19_P00316565	TSIX	−0.70329	0.000918	0.029195	−4.9834	Down	TSIX transcript, XIST antisense RNA
A_33_P3230189	SLITRK6	−0.43506	0.000921	0.029279	−4.98064	Down	SLIT and NTRK like family member 6
A_19_P00323540	#N/A	−0.29165	0.000924	0.029301	−4.97835	Down	NA
A_33_P3591761	FAM91A1	−0.29173	0.000929	0.029399	−4.97455	Down	family with sequence similarity 91 member A1
A_19_P00324420	H19_1	−0.3534	0.000939	0.029592	−4.9662	Down	H19 conserved region 1
A_33_P3269854	AL662860.1	−0.35472	0.00094	0.029592	−4.96563	Down	NA
A_19_P00315768	#N/A	−0.36314	0.000942	0.02963	−4.96409	Down	NA
A_23_P103532	GPR161	−0.44277	0.000943	0.029642	−4.96303	Down	G protein-coupled receptor 161
A_19_P00321398	AC131254.3	−0.52376	0.000948	0.02973	−4.95867	Down	NA
A_19_P00317780	LINC01539	−0.87469	0.00095	0.029758	−4.95741	Down	long intergenic non-protein coding RNA 1539
A_23_P500000	SCEL	−0.88136	0.00096	0.029949	−4.94922	Down	sciellin
A_33_P3389230	CXCR3	−0.34048	0.000961	0.029949	−4.9487	Down	C-X-C motif chemokine receptor 3
A_33_P3267320	RFT1	−0.30228	0.000964	0.029993	−4.94632	Down	RFT1 homolog
A_19_P00322508	AL355112.1	−0.72741	0.000974	0.030261	−4.93841	Down	NA
A_33_P3271276	PSG5	−0.53567	0.000982	0.030383	−4.93212	Down	pregnancy specific beta-1-glycoprotein 5

A_19_P00320343	AC091182.1	−0.29208	0.000982	0.030383	−4.93201	Down	NA
A_23_P88731	RAD51	−0.39275	0.000998	0.030653	−4.91932	Down	RAD51 recombinase
A_33_P3302025	ADAMTSL1	−0.46865	0.000999	0.030653	−4.91862	Down	ADAMTS like 1
A_23_P11697	HMGB4	−0.58702	0.001005	0.030726	−4.91408	Down	high mobility group box 4
A_33_P3236218	AC233724.12	−0.31016	0.00102	0.031027	−4.90284	Down	NA
A_23_P150935	TROAP	−0.33946	0.001025	0.031057	−4.89966	Down	trophinin associated protein
A_19_P00800553	#N/A	−0.4439	0.001027	0.031057	−4.89751	Down	NA
A_33_P3237744	SBK3	−0.30291	0.001028	0.031057	−4.89705	Down	SH3 domain binding kinase family member 3
A_33_P3399634	RASSF10	−0.42863	0.001028	0.031057	−4.89694	Down	Ras association domain family member 10
A_33_P3293138	SYPL1P2	−0.34807	0.001029	0.031057	−4.89668	Down	synaptophysin like 1 pseudogene 2
A_33_P3362178	PROX2	−0.52367	0.001042	0.031306	−4.88685	Down	prosperohomeobox 2
A_33_P3210418	SLC5A10	−0.36279	0.001045	0.031339	−4.88498	Down	solute carrier family 5 member 10
A_19_P00317612	LINC00211	−0.39501	0.001047	0.031385	−4.88288	Down	long intergenic non-protein coding RNA 211
A_19_P00801257	#N/A	−0.66576	0.00105	0.031385	−4.88109	Down	NA
A_23_P57110	SLC52A3	−0.69684	0.001051	0.031385	−4.88057	Down	solute carrier family 52 member 3
A_19_P00329700	#N/A	−0.31138	0.001085	0.032116	−4.8563	Down	NA
A_19_P00802359	#N/A	−0.45195	0.001105	0.032437	−4.84251	Down	NA
A_33_P3419399	ZC3H12D	−0.50596	0.001106	0.032437	−4.84152	Down	zinc finger CCCH-type containing 12D
A_33_P3423590	LOC100129275	−0.30291	0.001109	0.03248	−4.83993	Down	uncharacterized LOC100129275
A_19_P00813611	#N/A	−0.6284	0.001117	0.032517	−4.83402	Down	NA
A_33_P3807062	HJURP	−0.92005	0.001122	0.032573	−4.83105	Down	Holliday junction recognition protein
A_19_P00803592	#N/A	−0.30787	0.001123	0.032575	−4.83036	Down	NA
ERCC−00120_69	#N/A	−0.36448	0.001124	0.032575	−4.82936	Down	NA
A_19_P00326471	#N/A	−0.41537	0.001136	0.032752	−4.82119	Down	NA

A_19_P00327444	#N/A	−0.90056	0.001139	0.032752	−4.81923	Down	NA
A_24_P15325	XKR6	−0.40376	0.001144	0.032822	−4.81636	Down	XK related 6
A_23_P216118	UNC5D	−0.33057	0.001147	0.03288	−4.81426	Down	unc-5 netrin receptor D
A_23_P124300	BCO1	−0.39361	0.001155	0.03292	−4.80866	Down	beta-carotene oxygenase 1
A_19_P00809534	#N/A	−0.8039	0.001156	0.03292	−4.80844	Down	NA
A_33_P3250944	C1orf189	−0.54453	0.001156	0.03292	−4.80833	Down	chromosome 1 open reading frame 189
A_33_P3253139	VN1R33P	−0.69788	0.001159	0.03292	−4.80636	Down	vomeronasal 1 receptor 33 pseudogene
A_19_P00325561	LINC01814	−0.31826	0.001174	0.033199	−4.7967	Down	long intergenic non-protein coding RNA 1814
A_24_P322353	PSTPIP2	−0.38392	0.001175	0.033199	−4.79573	Down	proline-serine-threonine phosphatase interacting protein 2
A_23_P431505	XKR6	−0.45688	0.001181	0.033313	−4.79232	Down	XK related 6
A_33_P3320022	GALNTL6	−0.3736	0.001184	0.033352	−4.79001	Down	polypeptide N-acetylgalactosaminyltransferase-like 6
A_19_P00810308	#N/A	−0.52859	0.001185	0.033352	−4.78995	Down	NA
A_23_P376036	EXD1	−0.69087	0.001197	0.033513	−4.7819	Down	exonuclease 3'-5' domain containing 1
A_32_P70056	SPATA42	−1.07945	0.001203	0.033572	−4.77858	Down	spermatogenesis associated 42 (non-protein coding)
A_24_P924816	SLC2A13	−0.83023	0.001211	0.033718	−4.77338	Down	solute carrier family 2 member 13
A_19_P00326183	#N/A	−0.50802	0.001214	0.033752	−4.77163	Down	NA
A_23_P126888	KIF21B	−0.35514	0.001222	0.033852	−4.76647	Down	kinesin family member 21B
A_33_P3252083	GRIP1	−0.30525	0.001235	0.03403	−4.75838	Down	glutamate receptor interacting protein 1
A_33_P3231908	POU3F3	−1.22314	0.001245	0.034205	−4.75272	Down	POU class 3 homeobox 3
A_23_P305245	C3orf80	−0.92969	0.001246	0.034205	−4.75191	Down	chromosome 3 open reading frame 80
A_23_P98147	CPN1	−0.39421	0.00125	0.034238	−4.74955	Down	carboxypeptidase N subunit 1
A_23_P51085	SPC25	−0.89359	0.001265	0.0345	−4.74067	Down	SPC25, NDC80 kinetochore complex component
A_33_P3294911	C12ORF66	−0.75124	0.001272	0.03457	−4.73677	Down	chromosome 12 open reading frame 66
A_23_P94422	MELK	−1.00289	0.001289	0.034824	−4.72646	Down	maternal embryonic leucine zipper kinase

A_23_P94494	TLE4	−0.40005	0.00129	0.034824	−4.7262	Down	transducin like enhancer of split 4
A_23_P133123	MND1	−0.49815	0.00129	0.034824	−4.72607	Down	meiotic nuclear divisions 1
A_23_P113283	ZMAT3	−0.30962	0.001308	0.035031	−4.71552	Down	zinc finger matrin-type 3
A_19_P00321002	#N/A	−0.66499	0.001315	0.035072	−4.71182	Down	NA
A_24_P202319	ATP2A3	−0.53341	0.001317	0.03508	−4.71075	Down	ATPase sarcoplasmic/endoplasmic reticulum Ca2+ transporting 3
A_19_P00808287	#N/A	−0.58374	0.001319	0.035104	−4.70957	Down	NA
A_19_P00322255	AC027031.2	−0.30074	0.001324	0.035191	−4.70652	Down	NA
A_23_P113382	GRIP1	−0.48946	0.00133	0.035335	−4.70303	Down	glutamate receptor interacting protein 1
A_33_P3387831	CENPM	−0.34165	0.001335	0.03541	−4.70031	Down	centromere protein M
A_19_P00812133	#N/A	−0.46632	0.001337	0.035432	−4.69913	Down	NA
A_33_P3307133	GRIK2	−0.39228	0.00134	0.035451	−4.6979	Down	glutamate ionotropic receptor kainate type subunit 2
A_33_P3265872	AC002454.1	−0.41286	0.001341	0.035467	−4.697	Down	NA
A_33_P3233085	AL049775.3	−0.76663	0.001343	0.035471	−4.69629	Down	NA
A_33_P3229276	ZSCAN2	−0.34477	0.001347	0.035471	−4.69368	Down	zinc finger and SCAN domain containing 2
A_23_P305581	GPR26	−0.92643	0.001354	0.035473	−4.68974	Down	G protein-coupled receptor 26
A_33_P3363245	NXPH4	−0.59888	0.001356	0.035473	−4.68905	Down	neurexophilin 4
A_33_P3378972	UNC5D	−0.48731	0.001364	0.035577	−4.68463	Down	unc-5 netrin receptor D
A_19_P00315659	#N/A	−0.50683	0.001364	0.035577	−4.68448	Down	NA
A_33_P3301291	FOXB2	−0.65239	0.001367	0.035577	−4.68318	Down	forkhead box B2
A_33_P3273732	BX649632.1	−0.29717	0.001374	0.035707	−4.67918	Down	NA
A_33_P3354291	RNF215	−0.40503	0.001375	0.035711	−4.67845	Down	ring finger protein 215
A_19_P00320972	STMN1	−0.29389	0.001376	0.035711	−4.67818	Down	stathmin 1
A_23_P202894	ACRV1	−0.7613	0.00138	0.035747	−4.67571	Down	acrosomal vesicle protein 1
A_33_P3212316	#N/A	−0.3866	0.00138	0.035747	−4.67563	Down	NA

A_19_P00317630	AC007221.1	−0.88118	0.001395	0.036006	−4.668	Down	NA
A_19_P00328506	#N/A	−0.31695	0.001419	0.036362	−4.65535	Down	NA
A_23_P422933	ARHGAP20	−0.36989	0.001429	0.036533	−4.64983	Down	Rho GTPase activating protein 20
A_32_P151800	FAM72D	−0.61437	0.00144	0.036674	−4.64458	Down	family with sequence similarity 72 member D
A_19_P00810205	CCNB2P1	−0.39557	0.001449	0.036853	−4.63977	Down	cyclin B2 pseudogene 1
A_24_P14974	HTR3D	−0.5183	0.001466	0.036934	−4.63098	Down	5-hydroxytryptamine receptor 3D
A_23_P18152	ATP2B2	−0.37455	0.001473	0.037025	−4.62743	Down	ATPase plasma membrane Ca^{2+} transporting 2
A_19_P00321410	CCNG2	−0.46522	0.00148	0.037043	−4.6241	Down	cyclin G2
A_19_P00803108	#N/A	−0.77984	0.001502	0.037422	−4.61306	Down	NA
A_23_P46309	RCC1	−0.30159	0.001539	0.038129	−4.59539	Down	regulator of chromosome condensation 1
A_23_P59654	NPVF	−0.82435	0.00154	0.038143	−4.5947	Down	neuropeptide VF precursor
A_33_P3311755	KIF23	−0.8856	0.001544	0.038198	−4.59276	Down	kinesin family member 23
A_24_P22800	PSG11	−0.71926	0.001546	0.038198	−4.59192	Down	pregnancy specific beta-1-glycoprotein 11
A_33_P3210762	ADAMTS7	−0.32513	0.001549	0.038203	−4.59046	Down	ADAM metallopeptidase with thrombospondin type 1 motif 7
A_33_P3403075	PRR11	−0.3487	0.001559	0.038381	−4.58586	Down	proline rich 11
A_33_P3236077	CLRN2	−0.46427	0.00156	0.038397	−4.58513	Down	clarin 2
A_33_P3356816	LCE1B	−0.72876	0.001573	0.038509	−4.57935	Down	late cornified envelope 1B
A_19_P00813597	#N/A	−0.54523	0.001574	0.038509	−4.57879	Down	NA
A_19_P00800229	CRNDE	−0.91999	0.001577	0.038509	−4.57744	Down	colorectal neoplasia differentially expressed
A_24_P357468	TRAV8-2	−0.29523	0.001579	0.038509	−4.57665	Down	T-cell receptor alpha variable 8–2
A_19_P00329473	#N/A	−0.31401	0.00158	0.038509	−4.57618	Down	NA
A_19_P00322479	LINC01829	−0.63842	0.001582	0.038509	−4.57496	Down	long intergenic non-protein coding RNA 1829
A_19_P00328410	#N/A	−0.83049	0.001585	0.038521	−4.57347	Down	NA
A_33_P3390187	NHLRC4	−0.84198	0.001596	0.038662	−4.5687	Down	NHL repeat containing 4

A_23_P48414	CCNA1	−0.39159	0.001602	0.038728	−4.56561	Down	cyclin A1
A_33_P3302681	TLE4	−0.37711	0.001623	0.039071	−4.55641	Down	transducin like enhancer of split 4
A_33_P3772739	LINC00309	−0.82706	0.001638	0.03928	−4.54961	Down	long intergenic non-protein coding RNA 309
A_33_P3336552	AC063952.1	−1.05823	0.001655	0.039498	−4.54185	Down	NA
A_33_P3901921	PARPBP	−0.63626	0.001668	0.039656	−4.53606	Down	PARP1 binding protein
A_33_P3230188	#N/A	−0.85776	0.00167	0.039673	−4.53535	Down	NA
A_23_P207354	HIGD1B	−0.74427	0.001673	0.039727	−4.53395	Down	HIG1 hypoxia inducible domain family member 1B
A_19_P00316321	#N/A	−0.63455	0.001678	0.039754	−4.53181	Down	NA
A_19_P00315956	OXR1	−0.4835	0.001686	0.039774	−4.5284	Down	oxidation resistance 1
A_23_P59988	SLC35G5	−0.29464	0.001686	0.039774	−4.52825	Down	solute carrier family 35 member G5
A_23_P31816	DEFA3	−1.15384	0.001694	0.039849	−4.5248	Down	defensin alpha 3
A_33_P3400918	DISC1	−0.47671	0.001704	0.039925	−4.5206	Down	disrupted in schizophrenia 1
A_19_P00804027	#N/A	−0.60459	0.001719	0.040069	−4.51438	Down	NA
A_19_P00329041	#N/A	−0.56472	0.00172	0.040069	−4.51368	Down	NA
A_33_P3221019	ZAN	−0.31361	0.001755	0.040509	−4.49904	Down	zonadhesin (gene/pseudogene)
A_33_P3274731	CLTC	−0.79472	0.001759	0.040546	−4.49749	Down	clathrin heavy chain
A_23_P357794	MTBP	−0.50871	0.001772	0.040725	−4.49222	Down	MDM2 binding protein
A_19_P00810560	#N/A	−0.48498	0.001777	0.040786	−4.49004	Down	NA
A_19_P00320185	#N/A	−0.38737	0.001789	0.040916	−4.48506	Down	NA
A_23_P251486	COLEC10	−0.75326	0.001797	0.040993	−4.48197	Down	collectin subfamily member 10
A_23_P426835	LOC729652	−0.46781	0.001799	0.041013	−4.48122	Down	uncharacterized LOC729652
A_33_P3338047	PNPLA7	−0.35602	0.001802	0.041058	−4.48005	Down	patatin like phospholipase domain containing 7
A_33_P3238182	LINC00588	−0.73748	0.001803	0.041075	−4.47935	Down	long intergenic non-protein coding RNA 588
A_33_P3210827	RAD51D	−0.32694	0.00182	0.041335	−4.47282	Down	RAD51 paralog D

A_33_P3302662	TLE4	−0.46781	0.001846	0.041758	−4.46245	Down	transducin like enhancer of split 4
A_24_P119609	MYO1D	−0.33354	0.001857	0.041848	−4.45822	Down	myosin ID
A_33_P3214550	CXCR2	−0.69558	0.001859	0.041851	−4.45717	Down	C-X-C motif chemokine receptor 2
A_19_P00328681	#N/A	−0.48834	0.001873	0.041976	−4.45179	Down	NA
A_23_P114210	POU3F4	−0.72576	0.00188	0.042085	−4.44914	Down	POU class 3 homeobox 4
A_23_P212595	DCAF1	−0.51936	0.00188	0.042085	−4.44902	Down	DDB1 and CUL4 associated factor 1
A_32_P27917	KIF26A	−0.39357	0.001885	0.042112	−4.44716	Down	kinesin family member 26A
A_33_P3284247	GYG2P1	−0.35135	0.001887	0.042112	−4.44634	Down	glycogenin 2 pseudogene 1
A_33_P3223602	SATL1	−1.01787	0.001889	0.042112	−4.44575	Down	spermidine/spermine N1-acetyl transferase-like 1
A_33_P3357773	LINC01311	−0.64577	0.001899	0.042255	−4.44192	Down	long intergenic non-protein coding RNA 1311
A_19_P00800783	#N/A	−0.34959	0.0019	0.04226	−4.44147	Down	NA
A_33_P3307267	VWF	−1.17271	0.001905	0.042285	−4.43963	Down	von Willebrand factor
A_19_P00802201	CCNB2P1	−0.32921	0.001912	0.042367	−4.43691	Down	cyclin B2 pseudogene 1
A_23_P323898	ZSCAN12	−0.32731	0.001921	0.042445	−4.43356	Down	zinc finger and SCAN domain containing 12
A_23_P116587	OMP	−0.39895	0.001921	0.042445	−4.43341	Down	olfactory marker protein
A_33_P3377519	HOXA6	−0.30358	0.001936	0.042586	−4.42808	Down	homeobox A6
A_19_P00808893	#N/A	−0.39011	0.001937	0.042586	−4.42764	Down	NA
A_33_P3391126	SPINK5	−0.73247	0.001939	0.042586	−4.4269	Down	serine peptidase inhibitor, Kazal type 5
A_24_P276385	SPACA5	−0.33114	0.001943	0.042617	−4.42524	Down	sperm acrosome associated 5
A_24_P203315	CNIH2	−0.36997	0.001955	0.042739	−4.42083	Down	cornichon family AMPA receptor auxiliary protein 2
A_33_P3278053	MYBPC1	−0.40909	0.001956	0.042739	−4.42059	Down	myosin binding protein C, slow type
A_33_P3311235	ANKDD1B	−0.52417	0.001967	0.042841	−4.41666	Down	ankyrin repeat and death domain containing 1B
A_19_P00813205	#N/A	−0.39074	0.00197	0.042878	−4.41527	Down	NA
A_33_P3214446	RFC3	−0.37587	0.001981	0.043063	−4.41143	Down	replication factor C subunit 3

A_19_P00321076	PVT1	−0.46956	0.001992	0.043214	−4.40743	Down	Pvt1 oncogene (non-protein coding)
A_19_P00321010	LINC01133	−0.35717	0.002008	0.043379	−4.40148	Down	long intergenic non-protein coding RNA 1133
A_33_P3256287	FAM95B1	−0.73977	0.002011	0.04338	−4.40064	Down	family with sequence similarity 95 member B1
A_33_P3348794	FAM231A	−0.29791	0.002027	0.043605	−4.39472	Down	family with sequence similarity 231 member A
A_33_P3233886	#N/A	−0.46784	0.002052	0.043878	−4.38587	Down	NA
A_19_P00804574	#N/A	−0.3931	0.002065	0.044095	−4.38161	Down	NA
A_19_P00324503	#N/A	−0.31167	0.002074	0.044256	−4.37826	Down	NA
A_33_P3314341	KIAA1958	−0.42171	0.002101	0.044677	−4.36894	Down	KIAA1958
A_33_P3381613	#N/A	−0.32689	0.002116	0.044804	−4.36394	Down	NA
A_33_P3391418	TMEM40	−0.29537	0.002117	0.044804	−4.36359	Down	transmembrane protein 40
A_23_P148541	CTAG1A	−1.15888	0.002127	0.044805	−4.36026	Down	cancer/testis antigen 1A
A_19_P00321443	AC018742.1	−0.70496	0.002131	0.044805	−4.35898	Down	NA
A_19_P00812873	#N/A	−0.7287	0.002135	0.044826	−4.35764	Down	NA
A_33_P3719083	CHRNA7	−0.31719	0.002139	0.044894	−4.3562	Down	cholinergic receptor nicotinic alpha 7 subunit
A_24_P74070	PARD6G	−0.31074	0.002146	0.044971	−4.3539	Down	Par-6 family cell polarity regulator gamma
A_19_P00317759	AC104461.1	−0.89737	0.002151	0.045027	−4.35231	Down	NA
A_33_P3345309	PRSS56	−0.35326	0.002158	0.045125	−4.34969	Down	protease, serine 56
A_19_P00804601	TSSC2	−0.58666	0.002163	0.045173	−4.34823	Down	tumor suppressing subtransferable candidate 2 pseudogene
A_19_P00329437	#N/A	−0.59541	0.002165	0.045202	−4.34741	Down	NA
A_23_P429491	DDIAS	−0.4245	0.002191	0.045511	−4.33903	Down	DNA damage induced apoptosis suppressor
A_32_P196115	ZSCAN25	−0.29268	0.002196	0.045572	−4.33738	Down	zinc finger and SCAN domain containing 25
A_23_P208240	ZNF160	−0.37222	0.002215	0.045759	−4.33107	Down	zinc finger protein 160
A_19_P00326743	AL121655.1	−0.3691	0.002223	0.045831	−4.32845	Down	NA
A_33_P3407631	AC133565.1	−0.65338	0.002226	0.045834	−4.32755	Down	NA

A_33_P3296181	CCL3L3	−0.572	0.002227	0.045834	−4.32736	Down	C-C motif chemokine ligand 3 like 3
A_19_P00802881	#N/A	−0.41452	0.002228	0.045845	−4.32685	Down	NA
A_19_P00324996	#N/A	−0.29106	0.002233	0.045897	−4.32536	Down	NA
A_32_P62090	CCDC182	−0.31268	0.00225	0.046061	−4.32007	Down	coiled-coil domain containing 182
A_33_P3267270	TCEANC2	−0.33479	0.002255	0.04608	−4.31822	Down	transcription elongation factor A N-terminal and central domain containing 2
A_23_P71727	CKS2	−0.30604	0.002256	0.04608	−4.31802	Down	CDC28 protein kinase regulatory subunit 2
A_32_P120791	LOC727916	−0.38371	0.002261	0.04611	−4.31653	Down	uncharacterized LOC727916
A_23_P51580	HSD3B2	−0.56617	0.002263	0.046111	−4.31586	Down	hydroxy-delta-5-steroid dehydrogenase, 3 beta- and steroid delta-isomerase 2
A_33_P3415156	#N/A	−0.44703	0.002272	0.046219	−4.31314	Down	NA
A_33_P3419567	RAD21L1	−0.49999	0.00228	0.046295	−4.31051	Down	RAD21 cohesin complex component like 1
A_23_P118158	HS3ST2	−0.78188	0.002283	0.046327	−4.30943	Down	heparansulfate-glucosamine 3-sulfotransferase 2
A_24_P83899	GRM5	−0.6979	0.002291	0.046451	−4.30718	Down	glutamate metabotropic receptor 5
A_33_P3343496	OR5M1	−1.13651	0.002299	0.046535	−4.30454	Down	olfactory receptor family 5 subfamily M member 1
A_23_P252388	SPEF2	−0.34362	0.002329	0.046876	−4.29526	Down	sperm flagellar 2
A_33_P3254579	KRTAP1-5	−0.53907	0.002339	0.04696	−4.29225	Down	keratin associated protein 1-5
A_33_P3340926	KIR3DX1	−0.35799	0.002354	0.047043	−4.28758	Down	killer cell immunoglobulin like receptor, three Ig domains X1
A_33_P3305456	SSU72P2	−0.8223	0.002355	0.047043	−4.28742	Down	SSU72 pseudogene 2
A_33_P3281041	#N/A	−0.30238	0.002357	0.047054	−4.28686	Down	NA
A_24_P944973	EYS	−0.99854	0.002366	0.047061	−4.28409	Down	eyes shut homolog (Drosophila)
A_33_P3278896	#N/A	−0.3572	0.002381	0.047187	−4.27953	Down	NA
A_33_P3315979	OR4Q3	−0.71413	0.002409	0.047533	−4.27135	Down	olfactory receptor family 4 subfamily Q member 3
A_19_P00320885	AC091182.1	−0.34391	0.002412	0.04755	−4.27044	Down	NA
A_33_P3311676	LINC00703	−0.70239	0.002438	0.047883	−4.26282	Down	long intergenic non-protein coding RNA 703
A_33_P3363690	LINC01182	−0.7252	0.002439	0.047883	−4.26253	Down	long intergenic non-protein coding RNA 1182

A_33_P3213982	OR13J1	−0.85672	0.002442	0.0479	−4.26162	Down	olfactory receptor family 13 subfamily J member 1
A_23_P401024	LINC00304	−0.35474	0.002447	0.047944	−4.26028	Down	long intergenic non-protein coding RNA 304
A_24_P297537	MAMSTR	−0.40407	0.002451	0.047944	−4.2591	Down	MEF2 activating motif and SAP domain containing transcriptional regulator
A_23_P110802	CENPH	−0.52312	0.002457	0.047944	−4.25737	Down	centromere protein H
A_23_P58009	C3orf52	−0.46294	0.002461	0.047975	−4.25616	Down	chromosome 3 open reading frame 52
A_24_P472007	UNC13A	−0.32027	0.002474	0.048119	−4.25251	Down	unc-13 homolog A
A_24_P221485	PSAT1P3	−0.97228	0.002488	0.04829	−4.2483	Down	phosphoserine aminotransferase 1 pseudogene
A_33_P3298267	RUFY4	−0.33366	0.002489	0.04829	−4.24805	Down	RUN and FYVE domain containing 4
A_33_P3367606	FLJ13224	−0.47084	0.002493	0.048337	−4.24705	Down	uncharacterized LOC79857
A_24_P361427	TBC1D29	−0.37791	0.002515	0.048568	−4.24082	Down	TBC1 domain family member 29
A_33_P3256317	MYT1L-AS1	−0.71248	0.002517	0.048568	−4.24024	Down	MYT1L antisense RNA 1
A_19_P00317146	AC006116.8	−0.29745	0.00252	0.048568	−4.23944	Down	NA
A_33_P3236846	GPR17	−0.61794	0.002521	0.048568	−4.23892	Down	G protein-coupled receptor 17
A_33_P3372376	#N/A	−0.48123	0.002522	0.048568	−4.2387	Down	NA
A_33_P3328921	LINC00596	−0.29513	0.002524	0.048568	−4.23823	Down	long intergenic non-protein coding RNA 596
A_19_P00315998	#N/A	−1.11027	0.002554	0.048927	−4.22988	Down	NA
A_33_P3417474	CR381653.2	−1.16255	0.002554	0.048927	−4.22977	Down	NA
A_33_P3419577	RAD21L1	−0.45966	0.002558	0.04897	−4.22884	Down	RAD21 cohesin complex component like 1
A_19_P00801549	#N/A	−0.40986	0.00258	0.049254	−4.22271	Down	NA
A_33_P3325330	#N/A	−0.6559	0.002582	0.049254	−4.2222	Down	NA
A_33_P3292565	OR11H7	−0.35919	0.002597	0.049431	−4.21804	Down	olfactory receptor family 11 subfamily H member 7 (gene/pseudogene)
A_33_P3244096	CASK	−0.39817	0.002618	0.049598	−4.2122	Down	calcium/calmodulin dependent serine protein kinase
A_33_P3414107	AC123595.1	−0.86069	0.002619	0.049598	−4.21197	Down	NA
A_19_P00322418	OXR1	−0.41218	0.002621	0.049598	−4.21162	Down	oxidation resistance 1

A_19_P00802684	AC009413.1	−0.64286	0.002634	0.049766	−4.20802	Down	NA
A_33_P3401397	C7orf65	−0.62876	0.002646	0.049904	−4.20474	Down	chromosome 7 open reading frame 65

Table 2. The enriched pathway terms of the up-regulated differentially expressed genes.

Pathway ID	Pathway Name	P-value	FDR B&H	FDR B&Y	Bonferroni	Gene Count	Gene
BIOCYC							
545296	retinoate biosynthesis II	1.62E-03	4.52E-02	1.78E-01	4.52E-02	02	XDH,RBP4
545295	retinoate biosynthesis I	7.21E-03	1.01E-01	3.97E-01	2.02E-01	02	ALDH1A3,RBP4
142437	nicotine degradation IV	1.13E-02	1.06E-01	4.16E-01	3.18E-01	02	FMO5,AOX1
545294	the visual cycle I (vertebrates)	2.51E-02	1.54E-01	6.05E-01	7.02E-01	02	LRAT,RBP4
545293	retinol biosynthesis	3.18E-02	1.54E-01	6.05E-01	8.89E-01	02	LRAT,RBP4
142238	catecholamine biosynthesis	3.30E-02	1.54E-01	6.05E-01	9.24E-01	01	PNMT
545318	L-carnitine biosynthesis	4.91E-02	1.72E-01	6.74E-01	1.00E+00	01	BBOX1
545363	guanosine nucleotides degradation	4.91E-02	1.72E-01	6.74E-01	1.00E+00	01	XDH
KEGG							
1026136	Signaling pathways regulating pluripotency of stem cells	5.18E-04	5.12E-02	2.94E-01	8.97E-02	09	FGFR3,FGFR2,WNT5A,ID4,SOX2,INHBA,LEFTY2,PAX6,SMAD9
83053	Neuroactive ligand-receptor interaction	7.68E-04	5.12E-02	2.94E-01	1.33E-01	13	TSHB,CSH1,CSH2,FSHB,GALR1,SSTR2,GH1,GH2,GHRHR,KISS1R,GNRHR,CCKBR,P2RY2
83032	Drug metabolism - cytochrome P450	1.06E-03	5.12E-02	2.94E-01	1.84E-01	06	UGT2A2,MGST1,ALDH1A3,FMO5,AOX1,UGT2A1
83077	Jak-STAT signaling pathway	1.18E-03	5.12E-02	2.94E-01	2.05E-01	09	AOX1,CSH1,CSH2,IFNE,IL6R,IL20RA,IL22RA1,GH1,GH2
83031	Metabolism of xenobiotics by cytochrome P450	7.71E-03	2.56E-01	1.00E+00	1.00E+00	05	UGT2A2,MGST1,HSD11B1,ALDH1A3,UGT2A1
83096	Maturity onset diabetes of the young	8.89E-03	2.56E-01	1.00E+00	1.00E+00	03	NEUROD1,NKX2-2,PAX6
673221	Chemical carcinogenesis	1.18E-02	2.80E-01	1.00E+00	1.00E+00	05	UGT2A2,MGST1,HSD11B1,ALDH1A3,UGT2A1

83064	TGF-beta signaling pathway	1.29E-02	2.80E-01	1.00E+00	1.00E+00	05	ID4,INHBA,BMP7,LEFTY2,SMAD9
82940	Steroid hormone biosynthesis	1.57E-02	2.95E-01	1.00E+00	1.00E+00	04	UGT2A2,HSD11B1,HSD17B2,UGT2A1
172847	Protein digestion and absorption	1.70E-02	2.95E-01	1.00E+00	1.00E+00	05	COL4A6,COL13A1,SLC15A1,CTRB1,COL21A1
Pathway Interaction Database							
137983	ALK2 signaling events	1.37E-02	5.30E-01	1.00E+00	6.72E-01	02	BMP7,SMAD9
137948	BMP receptor signaling	2.16E-02	5.30E-01	1.00E+00	1.00E+00	03	BMP7,CHRDL1,SMAD9
REACTOME							
1268753	Peptide hormone biosynthesis	2.15E-06	1.11E-03	7.59E-03	1.11E-03	05	CGB3,TSHB,POMC,FSHB,INHBA
1268754	Glycoprotein hormones	3.31E-05	8.15E-03	5.56E-02	1.71E-02	04	CGB3,TSHB,FSHB,INHBA
1269559	Hormone ligand-binding receptors	4.72E-05	8.15E-03	5.56E-02	2.44E-02	04	TSHB,FSHB,GPHA2,GNRHR
1269328	Prolactin receptor signaling	1.16E-04	1.38E-02	9.41E-02	5.98E-02	04	CSH1,CSHL1,GH1,GH2
1268746	Peptide hormone metabolism	1.33E-04	1.38E-02	9.41E-02	6.89E-02	08	PCSK2,CGB3,TSHB,POMC,FSHB,INHBA,GH1,PAX6
1269327	Growth hormone receptor signaling	7.14E-04	6.16E-02	4.21E-01	3.70E-01	04	CSH1,CSHL1,GH1,GH2
1269545	Class A/1 (Rhodopsin-like receptors)	9.87E-04	7.30E-02	4.99E-01	5.11E-01	14	GPR37L1,TSHB,POMC,FSHB,GALR1,GPHA2,SSTR2,KISS1R,GNRHR,FFAR3,CCK,CCKBR,GAL,P2RY2
1270347	POU5F1 (OCT4), SOX2, NANOG activate genes related to proliferation	1.15E-03	7.42E-02	5.07E-01	5.93E-01	03	SALL1,SOX2,DPPA4
1269544	GPCR ligand binding	1.56E-03	8.98E-02	6.13E-01	8.08E-01	17	GPR37L1,TSHB,WNT5A,POMC,FSHB,IHH,GALR1,GPHA2,SSTR2,GHRHR,KISS1R,GNRHR,FFAR3,CCK,CCKBR,GAL,P2RY2
1270340	Regulation of gene expression in endocrine-committed (NEUROG3+) progenitor cells	2.66E-03	1.23E-01	8.36E-01	1.00E+00	02	NEUROD1,NKX2-2
GenMAPP							
MAP00350	Tyrosine metabolism	1.32E-02	2.47E-01	8.99E-01	2.78E-01	03	ALDH1A3,PNMT,AOX1

MAP00150	Androgen and estrogen metabolism	3.18E-02	2.47E-01	8.99E-01	6.67E-01	02	HSD11B1,HSD17B2
MAP00380	Tryptophan metabolism	4.08E-02	2.47E-01	8.99E-01	8.56E-01	03	AOX1,CYP4B1,TDO2
MAP00910	Nitrogen metabolism	4.70E-02	2.47E-01	8.99E-01	9.86E-01	02	CA8,CA12
MSigDB C2 BIOCARTA (v6.0)							
M5889	Ensemble of genes encoding extracellular matrix and extracellular matrix-associated proteins	1.18E-09	5.44E-08	2.40E-07	5.44E-08	45	S100A14,VIT,S100A1,S100B,MFAP4,FBN2,EFEMP1,FCN2,SBSPON,SPON1,FGF1,ITIH6,COL4A6,COL8A2,FRAS1,SFRP2,COL13A1,ITLN1,WNT5A,ADAM32,CSH1,CSH2,CSHL1,SLIT1,SLPI,IFNE,IHH,MEGF11,INHBA,LAMA1,ITIH2,GH1,GH2,BMP7,COL21A1,ADAMTS18,CHRDL1,CLEC9A,NTF4,OGN,LEFTY2,RSPO3,MEGF6,CBLN2,LGI1
M5883	Genes encoding secreted soluble factors	2.79E-07	6.41E-06	2.83E-05	1.28E-05	21	S100A14,S100A1,S100B,FGF1,SFRP2,WNT5A,CSH1,CSH2,CSHL1,IFNE,IHH,MEGF11,INHBA,GH1,GH2,BMP7,CHRDL1,NTF4,LEFTY2,MEGF6,CBLN2
M5884	Ensemble of genes encoding core extracellular matrix including ECM glycoproteins, collagens and proteoglycans	1.42E-05	2.18E-04	9.63E-04	6.54E-04	16	VIT,MFAP4,FBN2,EFEMP1,SBSPON,SPON1,COL4A6,COL8A2,FRAS1,COL13A1,SLIT1,LAMA1,COL21A1,OGN,RSPO3,LGI1
M5885	Ensemble of genes encoding ECM-associated proteins including ECM-affilaited proteins, ECM regulators and secreted factors	1.94E-05	2.23E-04	9.84E-04	8.91E-04	29	S100A14,S100A1,S100B,FCN2,FGF1,ITIH6,SFRP2,ITLN1,WNT5A,ADAM32,CSH1,CSH2,CSHL1,SLPI,IFNE,IHH,MEGF11,INHBA,ITIH2,GH1,GH2,BMP7,ADAMTS18,CHRDL1,CLEC9A,NTF4,LEFTY2,MEGF6,CBLN2
M3008	Genes encoding structural ECM glycoproteins	4.35E-04	4.00E-03	1.77E-02	2.00E-02	11	VIT,MFAP4,FBN2,EFEMP1,SBSPON,SPON1,FRAS1,SLIT1,LAMA1,RSPO3,LGI1

M4557	Biosynthesis of neurotransmitters	3.95E-03	3.03E-02	1.34E-01	1.82E-01	02	PNMT,TPH1
M3005	Genes encoding collagen proteins	5.99E-03	3.94E-02	1.74E-01	2.76E-01	04	COL4A6,COL8A2,COL13A1,COL21A1
M16518	Wnt/Ca2+/cyclic GMP signaling.	4.30E-02	2.47E-01	1.00E+00	1.00E+00	02	PDE6A,TF
PantherDB							
P02723	Adenine and hypoxanthine salvage pathway	5.47E-03	2.19E-01	9.36E-01	2.19E-01	02	AOX1,XDH
P04371	5-Hydroxytryptamine biosynthesis	4.91E-02	6.50E-01	1.00E+00	1.00E+00	01	TPH1
Pathway Ontology							
PW:0000365	melanocortin system	1.13E-02	1.52E-01	6.35E-01	4.08E-01	02	PCSK2,POMC
PW:0000040	androgen and estrogen metabolic	1.37E-02	1.52E-01	6.35E-01	4.94E-01	02	HSD11B1,UGT2A1
PW:0000371	calcium homeostasis	1.69E-02	1.52E-01	6.35E-01	6.09E-01	01	S100A1
PW:0000138	Vitamin B6 metabolic	1.69E-02	1.52E-01	6.35E-01	6.09E-01	01	AOX1
PW:0000200	the planar cell polarity Wntsignaling	2.51E-02	1.80E-01	7.53E-01	9.02E-01	02	CELSR1,VANGL2
PW:0000190	porphyrin and chlorophyll metabolic	4.91E-02	2.07E-01	8.63E-01	1.00E+00	01	UGT2A1
SMPDB							
SMP00063	Tryptophan Metabolism	2.59E-03	1.16E-01	5.13E-01	1.19E-01	03	AOX1,TDO2,TPH1
SMP00220	Xanthine Dehydrogenase Deficiency (Xanthinuria)	1.69E-02	1.16E-01	5.13E-01	7.78E-01	01	XDH
SMP00203	Molybdenium Cofactor Deficiency	1.69E-02	1.16E-01	5.13E-01	7.78E-01	01	XDH
SMP00246	Pirenzepine Pathway	3.18E-02	1.16E-01	5.13E-01	1.00E+00	02	ATP4A,CCKBR
SMP00232	Cimetidine Pathway	3.18E-02	1.16E-01	5.13E-01	1.00E+00	02	ATP4A,CCKBR
SMP00028	Caffeine Metabolism	3.30E-02	1.16E-01	5.13E-01	1.00E+00	01	XDH
SMP00233	Nizatidine Pathway	3.53E-02	1.16E-01	5.13E-01	1.00E+00	02	ATP4A,CCKBR
SMP00226	Omeprazole Pathway	3.53E-02	1.16E-01	5.13E-01	1.00E+00	02	ATP4A,CCKBR

SMP00231	Famotidine Pathway	3.53E-02	1.16E-01	5.13E-01	1.00E+00	02	ATP4A,CCKBR
SMP00227	Lansoprazole Pathway	3.53E-02	1.16E-01	5.13E-01	1.00E+00	02	ATP4A,CCKBR

Table 3 The enriched pathway terms of the down-regulated differentially expressed genes

Pathway ID	Pathway Name	P-value	FDR B&H	FDR B&Y	Bonferroni	Gene Count	Gene
BIOCYC							
545291	vitamin D3 biosynthesis	2.55E-02	2.81E-01	8.48E-01	2.81E-01	01	CYP27B1
KEGG							
83054	Cell cycle	1.09E-03	1.56E-01	8.62E-01	1.56E-01	07	TTK,CDC45,PLK1,PTTG2,BUB1,CCNA1,CCNB2
169306	Pancreatic secretion	7.88E-03	5.44E-01	1.00E+00	1.00E+00	05	RYR2,ATP2A3,ATP2B2,PLA2G2D,CELA3A
83055	p53 signaling pathway	1.19E-02	5.44E-01	1.00E+00	1.00E+00	04	GTSE1,CCNG2,ZMAT3,CCNB2
413367	Lactosylceramide biosynthesis	2.55E-02	5.44E-01	1.00E+00	1.00E+00	01	B4GALT6
413391	Cholecalciferol biosynthesis	2.55E-02	5.44E-01	1.00E+00	1.00E+00	01	CYP27B1
83050	Calcium signaling pathway	3.01E-02	5.44E-01	1.00E+00	1.00E+00	06	CHRNA7,RYR2,ATP2A3,ATP2B2,PLCD4,GRM5
377262	Fanconianemia pathway	3.36E-02	5.44E-01	1.00E+00	1.00E+00	03	FANCA,FANCD2,RAD51
119304	Progesterone-mediated oocyte maturation	3.51E-02	5.44E-01	1.00E+00	1.00E+00	04	PLK1,BUB1,CCNA1,CCNB2
199556	Vitamin digestion and absorption	3.77E-02	5.44E-01	1.00E+00	1.00E+00	02	SLC19A1,SLC52A3
413396	Steroid hormone biosynthesis, cholesterol =>prognenolone => progesterone	3.81E-02	5.44E-01	1.00E+00	1.00E+00	01	HSD3B2
83096	Maturity onset diabetes of the young	4.36E-02	5.67E-01	1.00E+00	1.00E+00	02	PKLR,PAX4
Pathway Interaction Database							
138080	Aurora B signaling	1.16E-09	4.30E-08	1.81E-07	4.30E-08	09	CENPA,KLHL13,KIF23,BIRC5,RACGAP1,NCAPG,BUB1,STMN1,KIF20A
137935	FOXM1 transcription factor network	6.86E-06	1.27E-04	5.33E-04	2.54E-04	06	CENPA,PLK1,FOXM1,BIRC5,NEK2,CCNB2

138007	PLK1 signaling events	1.93E-05	2.38E-04	1.00E-03	7.15E-04	06	PLK1,SPC24,TPX2,BUB1,CENPU,KIF20A
137925	Aurora A signaling	6.31E-04	5.83E-03	2.45E-02	2.33E-02	04	CENPA,BIRC5,TPX2,DLGAP5
137959	BARD1 signaling events	5.96E-03	4.41E-02	1.85E-01	2.20E-01	03	FANCA,FANCD2,RAD51
REACTOME							
1269820	Mitotic Prometaphase	1.04E-11	5.07E-09	3.43E-08	5.07E-09	15	KIF18A,CENPA,SPC25,CENPM,PLK1,BIRC5,SPC24,CENPH,CENPI,CDCA5,NUF2,NCAPG,BUB1,CENPU,CCNB2
1269821	Resolution of Sister Chromatid Cohesion	4.85E-11	1.18E-08	7.97E-08	2.36E-08	14	KIF18A,CENPA,SPC25,CENPM,PLK1,BIRC5,SPC24,CENPH,CENPI,CDCA5,NUF2,BUB1,CENPU,CCNB2
1269741	Cell Cycle	2.94E-10	4.76E-08	3.22E-07	1.43E-07	30	KIF18A,CENPA,HJURP,SPC25,MND1,HMMR,CDC45,CENPM,PLK1,FOXM1,KIF23,GTSE1,BIRC5,SPC24,CENPH,CENPI,TPX2,CDCA5,HAUS6,NUF2,NEK2,NCAPG,BUB1,CCNA1,RAD51,CENPU,RFC3,KIF20A,CCNB2,TOP2A
1269763	Cell Cycle, Mitotic	4.25E-10	5.16E-08	3.49E-07	2.07E-07	27	KIF18A,CENPA,SPC25,HMMR,CDC45,CENPM,PLK1,FOXM1,KIF23,GTSE1,BIRC5,SPC24,CENPH,CENPI,TPX2,CDCA5,HAUS6,NUF2,NEK2,NCAPG,BUB1,CCNA1,CENPU,RFC3,KIF20A,CCNB2,TOP2A
1269519	RHO GTPases Activate Formins	3.21E-08	3.12E-06	2.11E-05	1.56E-05	12	KIF18A,CENPA,SPC25,CENPM,PLK1,BIRC5,SPC24,CENPH,CENPI,NUF2,BUB1,CENPU
1269378	Kinesins	6.18E-08	5.01E-06	3.39E-05	3.00E-05	08	KIF18A,KIF23,RACGAP1,KIF21B,KIF26A,KIF15,KIF11,KIF20A
1269826	Separation of Sister Chromatids	2.79E-07	1.94E-05	1.31E-04	1.36E-04	13	KIF18A,CENPA,SPC25,CENPM,PLK1,BIRC5,SPC24,CENPH,CENPI,CD

							CA5,NUF2,BUB1,CENPU
1269810	M Phase	5.07E-07	2.98E-05	2.01E-04	2.46E-04	17	KIF18A,CENPA,SPC25,CENPM,PLK1,KIF23,BIRC5,SPC24,CENPH,CENPI,CDCA5,NUF2,NCAPG,BUB1,CENPU,KIF20A,CCNB2
1269825	Mitotic Anaphase	5.75E-07	2.98E-05	2.01E-04	2.79E-04	13	KIF18A,CENPA,SPC25,CENPM,PLK1,BIRC5,SPC24,CENPH,CENPI,CDCA5,NUF2,BUB1,CENPU
1269823	Mitotic Metaphase and Anaphase	6.12E-07	2.98E-05	2.01E-04	2.98E-04	13	KIF18A,CENPA,SPC25,CENPM,PLK1,BIRC5,SPC24,CENPH,CENPI,CDCA5,NUF2,BUB1,CENPU
1269799	G2/M Transition	6.15E-04	1.42E-02	9.62E-02	2.99E-01	09	HMMR,PLK1,FOXM1,GTSE1,TPX2,HAUS6,NEK2,CCNA1,CCNB2
MSigDB C2 BIOCARTA (v6.0)							
M17370	Role of Ran in mitotic spindle regulation	2.34E-04	8.19E-03	3.40E-02	8.19E-03	03	RCC1,TPX2,KIF15
M16334	Eph Kinases and ephrins support platelet aggregation	6.90E-03	1.21E-01	5.01E-01	2.42E-01	02	L1CAM,EPHB1
M9703	Role of BRCA1, BRCA2 and ATR in Cancer Susceptibility	2.94E-02	3.43E-01	1.00E+00	1.00E+00	02	FANCD2,RAD51
PantherDB							
P00031	Inflammation mediated by chemokine and cytokine signaling pathway	1.17E-02	3.74E-01	1.00E+00	3.74E-01	07	PF4,VWF,CCL3L3,CASK,CXCR2,CXCR3,PLCD4
Pathway Ontology							
PW:0000194	O-Glycans biosynthetic	1.30E-02	5.02E-02	1.95E-01	3.52E-01	01	B4GALT6
PW:0000164	ganglioside biosynthetic	1.30E-02	5.02E-02	1.95E-01	3.52E-01	01	B4GALT6
PW:0000196	globoside metabolic	1.30E-02	5.02E-02	1.95E-01	3.52E-01	01	B4GALT6
PW:0000197	glycosphingolipid metabolic	1.30E-02	5.02E-02	1.95E-01	3.52E-01	01	B4GALT6
PW:0000161	glycosylphosphatidylinositol anchor biosynthetic	1.30E-02	5.02E-02	1.95E-01	3.52E-01	01	B4GALT6
PW:0000162	glycosphingolipid biosynthetic-lactoseries	1.30E-02	5.02E-02	1.95E-01	3.52E-01	01	B4GALT6

PW:0000163	glycosphingolipid biosynthetic-neolactoseries	1.30E-02	5.02E-02	1.95E-01	3.52E-01	01	B4GALT6
PW:0000192	N-Glycans biosynthetic	2.55E-02	8.04E-02	3.13E-01	6.90E-01	01	B4GALT6
PW:0000017	Huntington disease	2.68E-02	8.04E-02	3.13E-01	7.24E-01	02	CLTC,BDNF
SMPDB							
SMP00266	Eptifibatide Pathway	9.96E-03	9.96E-03	1.05E-01	4.94E-01	02	RYR2,PLCD4
SMP00261	Ticlopidine Pathway	9.96E-03	9.96E-03	1.05E-01	4.94E-01	02	RYR2,PLCD4
SMP00265	Abciximab Pathway	9.96E-03	9.96E-03	1.05E-01	4.94E-01	02	RYR2,PLCD4
SMP00264	Dipyridamole Pathway	9.96E-03	9.96E-03	1.05E-01	4.94E-01	02	RYR2,PLCD4
SMP00263	Cilostazol Pathway	9.96E-03	9.96E-03	1.05E-01	4.94E-01	02	RYR2,PLCD4
SMP00267	Tirofiban Pathway	9.96E-03	9.96E-03	1.05E-01	4.94E-01	02	RYR2,PLCD4
SMP00336	Vitamin A Deficiency	1.30E-02	1.30E-02	1.17E-01	5.54E-01	01	BCO1
SMP00260	Clopidogrel Pathway	2.19E-02	2.19E-02	1.73E-01	8.17E-01	02	RYR2,PLCD4
SMP00367	Carvedilol Pathway	2.94E-02	2.94E-02	1.85E-01	8.75E-01	02	RYR2,PLCD4
SMP00368	Labetalol Pathway	2.94E-02	2.94E-02	1.85E-01	8.75E-01	02	RYR2,PLCD4

Table 4. The enriched GO terms of the up-regulated differentially expressed genes.

GO ID	CATEGORY	GO Name	P Value	FDR B&H	FDR B&Y	Bonferroni	Gene Count	Gene
GO:0090596	BP	sensory organ morphogenesis	1.00E-12	4.43E-09	3.97E-08	4.43E-09	28	EYA4,FBN2,EFEMP1,SALL1,CLIC5,TMIE,FGFR3,FGFR2,ALDH1A3,CLRN1,PRRX2,COL8A2,SDK2,WNT5A,CELSR1,IHH,MEGF11,SOX2,SOX9,GAS1,BMP7,FOXL2,STRA6,CALB1,ROR2,RBP4,VANGL2,PAX6

GO:0048562	BP	embryonic organ morphogenesis	1.04E-11	2.29E-08	2.06E-07	4.58E-08	28	EYA4,GRHL2,OVOL2,FBN2,EFEMP1,SALL1,CLIC5,TMIE,FGFR2,ALDH1A3,CLRN1,PRRX2,WNT5A,CELSR1,IHH,RNF207,SOX2,SOX9,GAS1,BMP7,NEUROD1,FOXL2,TBXT,STRA6,ROR2,RBP4,VANGL2,PAX6
GO:0048568	BP	embryonic organ development	5.52E-11	7.17E-08	6.43E-07	2.44E-07	33	EYA4,GRHL2,OVOL2,FBN2,EFEMP1,SALL1,CLIC5,TMIE,FGFR2,ALDH1A3,CLRN1,PRRX2,WNT5A,CELSR1,IHH,RNF207,SOX2,SOX9,PLAC1,GAS1,BMP7,NEUROD1,FOXL2,NRK,TBXT,STRA6,KRT19,ROR2,RBP4,RUNX1,VANGL2,RSPO3,PAX6
GO:0007423	BP	sensory organ development	6.48E-11	7.17E-08	6.43E-07	2.87E-07	37	EYA4,PDE6A,GRHL2,FBN2,EFEMP1,SALL1,CLIC5,TMIE,FGFR3,FGFR2,ALDH1A3,CLRN1,PRRX2,COL8A2,SDK2,WNT5A,CELSR1,IHH,PRDM16,MEGF11,SOX2,SOX9,INHBA,GAS1,LAMA1,BMP7,NEUROD1,FOXL2,ADAMTS18,CHRDL1,STRA6,CALB1,NTF4,ROR2,RBP4,VANGL2,PAX6
GO:0048598	BP	embryonic morphogenesis	7.43E-10	6.57E-07	5.89E-06	3.29E-06	37	EYA4,EYA2,GRHL2,OVOL2,FBN2,EFEMP1,SALL1,CLIC5,TMIE,AMOT,FGFR2,ALDH1A3,CLRN1,PRRX2,FRAS1,SFRP2,WNT5A,TRIM71,IRX1,CELSR1,IHH,RNF207,SOX2,SOX9,INHBA,GAS1,BMP7,NEUROD1,FOXL2,TBXT,STRA6,ROR2,RBP4,VANGL2,RSPO3,PAX6,IRX2
GO:0009887	BP	animal organ morphogenesis	3.37E-09	2.28E-06	2.05E-05	1.49E-05	50	EYA4,GRHL2,PGR,OVOL2,FBN2,EFEMP1,SALL1,CLIC5,TMIE,FGF1,FGFR3,FGFR2,FLRT2,ALDH1A3,CLRN1,PRRX2,COL8A2,FRAS1,SDK2,

								SFRP2,COL13A1,WNT5A,ID4,IRX1,CELSR1,SLIT1,IHH,RNF207,MEGF11,SOX2,SOX9,INHBA,GAS1,LAMA1,BMP7,NEUROD1,FOXL2,NGFR,TBXT,STRA6,CALB1,NTF4,ROR2,RBP4,VANGL2,RSPO3,TGFBR3,PAX6,LY6H,IRX2
GO:0048592	BP	eye morphogenesis	3.61E-09	2.28E-06	2.05E-05	1.60E-05	18	FBN2,EFEMP1,FGFR3,ALDH1A3,COL8A2,SDK2,WNT5A,IHH,MEGF11,SOX2,SOX9,GAS1,BMP7,FOXL2,STRA6,CALB1,RBP4,PAX6
GO:0009790	BP	embryo development	5.07E-09	2.80E-06	2.51E-05	2.24E-05	50	LRAT,ERBB4,EYA4,EYA2,GRHL2,OVOL2,FBN2,EFEMP1,SALL1,CLIC5,TMIE,AMOT,FGFR2,ALDH1A3,HSD17B2,CLRN1,PRRX2,FRAS1,SFRP2,WNT5A,TRIM71,IRX1,CELSR1,IHH,RNF207,SOX2,SOX5,SOX9,PLAC1,INHBA,GAS1,LAMA1,DLK1,BMP7,NEUROD1,FOXL2,NRK,TBXT,STRA6,KRT19,ROR2,RBP4,RUNX1,CUBN,VANGL2,RSPO3,TGFBR3,P2RY2,PAX6,IRX2
GO:0060429	BP	epithelium development	7.84E-09	3.85E-06	3.45E-05	3.47E-05	54	ERBB4,DACT2,PTER,GRHL2,PGR,OVOL2,SALL1,CLIC5,NR0B1,FGF1,FGFR3,FGFR2,ALDH1A3,CLRN1,DEUP1,FRAS1,SFRP2,WNT5A,AGR2,ID4,XDH,TRIM71,IRX1,CELSR1,IHH,RNF207,SOX2,SOX9,PRSS8,INHBA,GAS1,LAMA1,BMP7,NEUROD1,FOXL2,NGFR,TBXT,NKX2-2,KAZN,STRA6,KRT14,CALB1,NTF4,ROR2,OCA2,RUNX1,VANGL2,RSPO3,TGFBR3,ZNF750,GAL,PAX6,IRX2,SMAD9
GO:0072089	BP	stem cell proliferation	1.16E-08	5.13E-06	4.60E-05	5.13E-05	17	OVOL2,FGFR2,PRRX2,SFRP2,WNT5A,ID4,TRIM71,CSHL1,IHH,SOX2,SOX5,SOX9,GAS1,BMP7,KCNA1,RUNX1,

								PAX6
GO:0031012	CC	extracellular matrix	1.21E-08	4.58E-06	2.98E-05	4.58E-06	28	VIT,MFAP4,FBN2,EFEMP1,SBSPON,SPON1,FGF1,FGFR2,FLRT2,COL4A6,COL8A2,FRAS1,SFRP2,WNT5A,SLIT1,SLPI,IHH,LAMA1,BMP7,COL21A1,ADAMTS18,CPXM2,LAD1,RUNX1,OGN,TF,LEFTY2,TGFBR3
GO:0005615	CC	extracellular space	1.37E-07	2.60E-05	1.69E-04	5.19E-05	55	PCSK2,CRISP2,SCRG1,TAC4,F5,S100B,EFEMP1,FCN2,SPON1,FGF1,FLRT2,SPINK8,SFRP2,PI16,WNT5A,AGR2,POMC,PON3,XDH,APOD,CARTPT,SCGB1D2,SCGB1D1,CSHL1,ASIP,SLIT1,SLPI,C2orf40,IFNE,IHH,CTRB1,ATP4A,IL6R,PRSS8,INHBA,LAMA1,DLK1,ITIH2,SMR3B,GH1,BMP7,KLK11,CPXM2,RBP4,KLK12,FAM3B,OGN,TF,CCK,LEFTY2,TGFBR3,GAL,DMKN,CBLN2,LGI1
GO:0044420	CC	extracellular matrix component	2.57E-04	3.26E-02	2.13E-01	9.78E-02	10	MFAP4,FBN2,EFEMP1,COL4A6,COL8A2,FRAS1,LAMA1,LAD1,RUNX1,TF
GO:0005604	CC	basement membrane	8.42E-04	8.00E-02	5.21E-01	3.20E-01	08	EFEMP1,COL4A6,COL8A2,FRAS1,LAMA1,LAD1,RUNX1,TF
GO:0043204	CC	perikaryon	1.16E-03	8.84E-02	5.76E-01	4.42E-01	09	PCSK2,PGR,RBFOX3,PNMT,CNTNAP2,KCNA1,NGFR,CACNA1A,CCK
GO:0005788	CC	endoplasmic reticulum lumen	1.46E-03	9.27E-02	6.04E-01	5.56E-01	11	TRDN,F5,SPON1,SRL,COL4A6,COL8A2,COL13A1,WNT5A,ERP27,COL21A1,NTF4
GO:0034703	CC	cation channel complex	4.96E-03	2.47E-01	1.00E+00	1.00E+00	09	TRDN,SCN2B,CNTNAP2,CACNA2D3,KCNA1,KCNG1,KCNJ16,KCNS2,CACNA1A
GO:0005783	CC	endoplasmic reticulum	7.05E-03	2.47E-01	1.00E+00	1.00E+00	45	SPTLC3,LRAT,CPED1,TRDN,F5,PGR,S100A1,MGST1,SPON1,TMPRSS3,SRL,PLA2G4A,FGFR3,FLRT2,HSD11B1,HSD17B2,SLC51A,FXYD3,ALDOB,COL4A6,COL8A2,COL13A1,FMO5,WNT5A,AGR2

								,XDH,APOD,CSH1,CSH2,AGMO,PRSS50,CYP4B1,KCNA1,ERP27,COL21A1,NGFR,NTF4,CH25H,AGR3,OCA2,RNF175,CUBN,TGFBR3,RASGRP1,SEZ6L
GO:0090665	CC	glycoprotein complex	7.07E-03	2.47E-01	1.00E+00	1.00E+00	03	SGCD,KRT19,SNTG2
GO:0031226	CC	intrinsic component of plasma membrane	0.02817	3.24E-01	1.00E+00	1.00E+00	42	KLRF1,FCER1A,SCN2B,FGFR3,FGFR2,FLRT2,FXYD3,COL13A1,SGCD,AQP4,CELSR1,ART3,SLC15A1,CNTNAP2,SLC13A5,ATP4A,IL6R,TRABD2B,PRSS8,GALR1,GAS1,SSTR2,SLC16A12,SLC39A8,KCNA1,KISS1R,KCNG1,KCNJ12,KCNJ16,NGFR,KCNS2,GNRHR,GPR12,FFAR3,ROR2,CCKBR,TGFBR3,SLC39A12,CD8B,P2RY2,TSPAN8,SLC6A14
GO:0005179	MF	hormone activity	4.87E-08	3.94E-05	2.87E-04	3.94E-05	14	CGB3,TSHB,POMC,CARTPT,CSH1,CSH2,CSHL1,FSHB,INHBA,GPHA2,GH1,GH2,CCK,GAL
GO:0005102	MF	signaling receptor binding	4.24E-06	1.71E-03	1.25E-02	3.43E-03	54	ERBB4,CGB3,S100A14,TSHB,TAC4,TRDN,PGR,S100B,EFEMP1,PPP1R1B,NR0B1,NGEF,FGF1,FLRT2,SFRP2,WNT5A,AGR2,POMC,ALKAL2,CARTPT,CSH1,CSH2,CSHL1,ASIP,FSHB,SLIT1,RASL11B,IFNE,IHH,IL6R,INHBA,LAMA1,GPHA2,GH1,GH2,BMP7,FOXL2,NGFR,NTF4,ROR2,FAM3B,OGN,TF,CCK,CCKBR,LEFTY2,RSPO3,TGFBR3,GAL,CD8B,P2RY2,TSPAN8,SNTG2,LGI1
GO:0004854	MF	xanthine dehydrogenase activity	3.25E-04	6.58E-02	4.79E-01	2.63E-01	2	AOX1,XDH
GO:0016726	MF	oxidoreductase activity, acting on	3.25E-04	6.58E-02	4.79E-01	2.63E-01	2	AOX1,XDH

		CH or CH2 groups, NAD or NADP as acceptor						
GO:0004864	MF	protein phosphatase inhibitor activity	5.51E-04	8.91E-02	6.48E-01	4.46E-01	5	PPP1R1B,PHACTR3,PHACTR2,PPP1R17,PPP1R27
GO:0019212	MF	phosphatase inhibitor activity	7.87E-04	1.04E-01	7.58E-01	6.37E-01	5	PPP1R1B,PHACTR3,PHACTR2,PPP1R17,PPP1R27
GO:0044548	MF	S100 protein binding	1.42E-03	1.04E-01	7.58E-01	1.00E+00	3	S100A1,S100B,FGF1
GO:0008236	MF	serine-type peptidase activity	1.45E-03	1.04E-01	7.58E-01	1.00E+00	12	PCSK2,F5,FCN2,TMPRSS3,PRSS45,PRSS50,CTRB1,PRSS8,RHBDL3,KLK11,CPXM2,KLK12
GO:0017171	MF	serine hydrolase activity	1.61E-03	1.04E-01	7.58E-01	1.00E+00	12	PCSK2,F5,FCN2,TMPRSS3,PRSS45,PRSS50,CTRB1,PRSS8,RHBDL3,KLK11,CPXM2,KLK12
GO:0004252	MF	serine-type endopeptidase activity	1.77E-03	1.04E-01	7.58E-01	1.00E+00	11	PCSK2,F5,FCN2,TMPRSS3,PRSS45,PRSS50,CTRB1,PRSS8,RHBDL3,KLK11,KLK12
GO:0031781	MF	type 3 melanocortin receptor binding	1.87E-03	1.04E-01	7.58E-01	1.00E+00	2	POMC,ASIP

Note: Biological Process(BP), Cellular Component(CC) and Molecular Functions (MF).

Table 5. The enriched GO terms of the down-regulated differentially expressed genes.

GO ID	CATEGORY	GO Name	P Value	FDR B&H	FDR B&Y	Bonferroni	Gene Count	Gene
GO:0000280	BP	nuclear division	2.87E-18	6.44E-15	5.58E-14	9.45E-15	41	NUSAP1,KIF18A,SPC25,RCC1,TTK,FANCA,FANCD2,CKS2,KLHL13,CLTC,PLK1,KIF23,BIRC5,SPC24,CENPH,TPX2,RACGAP1,MTBP,CDCA5,PTTG2,HAUS6,DLGAP5,PBK,RAD21L1,NUF2,NEK2,PTTG3P,NCAPG,KIF15,BUB1,CCNA1,KIF14,KIF11,RAD51,RAD51D,PRC1,EDN1,CCNF,CCNG2,CCNB2,TOP2A

GO:0048285	BP	organelle fission	3.91E-18	6.44E-15	5.58E-14	1.29E-14	42	NUSAP1,KIF18A,SPC25,RCC1,TTK,FANCA,FANCD2,CKS2,KLHL13,CLTC,PLK1,KIF23,BIRC5,SPC24,CENPH,TPX2,RACGAP1,MTBP,MTFR2,CDCA5,PTTG2,HAUS6,DLGAP5,PBK,RAD21L1,NUF2,NEK2,PTTG3P,NCAPG,KIF15,BUB1,CCNA1,KIF14,KIF11,RAD51,RAD51D,PRC1,EDN1,CCNF,CCNG2,CCNB2,TOP2A
GO:0007059	BP	chromosome segregation	1.04E-17	1.14E-14	9.91E-14	3.43E-14	31	NUSAP1,KIF18A,CENPA,HJURP,SPC25,RCC1,TTK,FANCD2,CENPM,PLK1,KIF23,BIRC5,SPC24,CENPH,CENPI,RACGAP1,MTBP,CDCA5,PTTG2,DLGAP5,RAD21L1,NUF2,NEK2,PTTG3P,NCAPG,BUB1,KIF14,KIF11,CENPU,PRC1,TOP2A
GO:0098813	BP	nuclear chromosome segregation	8.97E-17	7.37E-14	6.40E-13	2.95E-13	28	NUSAP1,KIF18A,CENPA,SPC25,TTK,FANCD2,CENPM,PLK1,KIF23,BIRC5,SPC24,CENPH,CENPI,RACGAP1,MTBP,CDCA5,PTTG2,DLGAP5,RAD21L1,NUF2,NEK2,PTTG3P,NCAPG,BUB1,KIF14,CENPU,PRC1,TOP2A
GO:0022402	BP	cell cycle process	1.35E-15	8.90E-13	7.72E-12	4.45E-12	58	NUSAP1,CDKN3,KIF18A,CENPA,SPC25,RCC1,HMMR,TTK,CDC45,FANCA,FANCD2,CKS2,CENPM,KLHL13,ZC3H12D,CLTC,PLK1,FOXM1,KIF23,GTSE1,BIRC5,SPC24,CENPH,CENPI,TPX2,RACGAP1,MTBP,CDCA5,PTTG2,SOX4,HAUS6,CYP27B1,DLGAP5,PBK,RAD21L1,NUF2,MELK,NEK2,PTTG3P,NCAPG,KIF15,BUB1,CCNA1,KIF14,SLC39A5,KIF11,RAD51,RAD51D,CENPU,STMN1,PRC1,EDN1,KIF20A,CCNF,CCNG2,DDIAS,CCNB2,TOP2A
GO:0000819	BP	sister chromatid segregation	2.90E-15	1.59E-12	1.38E-11	9.53E-12	24	NUSAP1,KIF18A,CENPA,SPC25,TTK,CENPM,PLK1,KIF23,BIRC5,SPC24,CENPH,CENPI,RACGAP1,MTBP,CDCA5,DLGAP5,NUF2,NEK2,NCAPG,BUB1,KIF14

							,CENPU,PRC1,TOP2A
GO:1903047	BP	mitotic cell cycle process	2.55E-14	1.20E-11	1.04E-10	8.38E-11	45	NUSAP1,CDKN3,KIF18A,CENPA,SPC25,RCC1,HMMR,TTK,CDC45,CKS2,KLHL13,ZC3H12D,CLTC,PLK1,FOXM1,KIF23,GTSE1,BIRC5,SPC24,CENPH,TPX2,RACGAP1,MTBP,CDCA5,SOX4,HAUS6,DLGAP5,PBK,NUF2,MELK,NEK2,NCAPG,KIF15,BUB1,CCNA1,KIF14,KIF11,STMN1,PRC1,EDN1,KIF20A,CCNF,CCNG2,CCNB2,TOP2A
GO:0007049	BP	cell cycle	3.07E-14	1.26E-11	1.09E-10	1.01E-10	64	NUSAP1,CDKN3,KIF18A,CENPA,EXD1,HJURP,SPC25,RCC1,MND1,HMMR,TTK,CDC45,FANCA,FANCD2,CKS2,CENPM,KLHL13,ZC3H12D,CLTC,PLK1,FOXM1,KIF23,GTSE1,BIRC5,SPC24,CENPH,CENPI,TPX2,RACGAP1,MTBP,PRR11,CDCA5,PTTG2,SOX4,HAUS6,CYP27B1,DLGAP5,PBK,RAD21L1,PARD6G,NUF2,MELK,PTP4A1,NEK2,PTTG3P,NCAPG,KIF15,BUB1,CCNA1,KIF14,SLC39A5,KIF11,RAD51,RAD51D,CENPU,STMN1,PRC1,EDN1,KIF20A,CCNF,CCNG2,DDIAS,CCNB2,TOP2A
GO:0000278	BP	mitotic cell cycle	5.56E-13	2.03E-10	1.76E-09	1.83E-09	45	NUSAP1,CDKN3,KIF18A,CENPA,SPC25,RCC1,HMMR,TTK,CDC45,CKS2,KLHL13,ZC3H12D,CLTC,PLK1,FOXM1,KIF23,GTSE1,BIRC5,SPC24,CENPH,TPX2,RACGAP1,MTBP,CDCA5,SOX4,HAUS6,DLGAP5,PBK,NUF2,MELK,NEK2,NCAPG,KIF15,BUB1,CCNA1,KIF14,KIF11,STMN1,PRC1,EDN1,KIF20A,CCNF,CCNG2,CCNB2,TOP2A
GO:0051301	BP	cell division	2.86E-12	9.41E-10	8.16E-09	9.41E-09	35	NUSAP1,SPC25,RCC1,TTK,CKS2,KLHL13,PLK1,KIF23,BIRC5,POU3F2,POU3F3,PPBP,SPC24,TPX2,RACGAP1,MTBP,CDCA5,HAUS6,DLGAP5,PARD6G,NUF2,NEK2,NCAPG,BUB1,CCNA1,KIF14,KIF11,STMN1,PRC1,ED

								N1,KIF20A,CCNF,CCNG2,CCNB2,TOP2A
GO:0000793	CC	condensed chromosome	5.65E-11	2.22E-08	1.46E-07	2.22E-08	19	CENPA,HJURP,SPC25,RCC1,FANCD2,CENPM,PLK1,BIRC5,SPC24,CENPH,CDCA5,RAD21L1,NUF2,NEK2,NCAPG,BUB1,RAD51,CENPU,TOP2A
GO:0000776	CC	kinetochore	1.20E-10	2.36E-08	1.55E-07	4.72E-08	15	KIF18A,CENPA,HJURP,SPC25,CENPM,PLK1,BIRC5,SPC24,CENPH,CENPI,MTBP,NUF2,NEK2,BUB1,CENPU
GO:0000775	CC	chromosome, centromeric region	4.29E-09	5.33E-07	3.49E-06	1.69E-06	16	KIF18A,CENPA,HJURP,SPC25,CENPM,PLK1,BIRC5,SPC24,CENPH,CENPI,MTBP,CDCA5,NUF2,NEK2,BUB1,CENPU
GO:0000777	CC	condensed chromosome kinetochore	5.42E-09	5.33E-07	3.49E-06	2.13E-06	12	CENPA,HJURP,SPC25,CENPM,PLK1,BIRC5,SPC24,CENPH,NUF2,NEK2,BUB1,CENPU
GO:0005819	CC	spindle	7.20E-09	5.66E-07	3.71E-06	2.83E-06	20	NUSAP1,KIF18A,TTK,CLTC,PLK1,KIF23,POC1A,BIRC5,TPX2,RACGAP1,HAUS6,DLGAP5,PTP4A1,NEK2,KIF15,KIF14,KIF11,RASSF10,PRC1,KIF20A
GO:0000779	CC	condensed chromosome, centromeric region	1.72E-08	1.13E-06	7.38E-06	6.76E-06	12	CENPA,HJURP,SPC25,CENPM,PLK1,BIRC5,SPC24,CENPH,NUF2,NEK2,BUB1,CENPU
GO:0005871	CC	kinesin complex	4.58E-08	2.57E-06	1.69E-05	1.80E-05	09	KIF18A,KIF23,KIF21B,KIF26A,DISC1,KIF15,KIF14,KIF11,KIF20A
GO:0015630	CC	microtubule cytoskeleton	1.43E-07	7.01E-06	4.59E-05	5.61E-05	38	NUSAP1,KIF18A,SPEF2,TTK,CDC45,CLTC,C12orf66,PLK1,KIF23,POC1A,GTSE1,BIRC5,TPX2,RACGAP1,KIF21B,HAUS6,KIF26A,DISC1,DLGAP5,DAB1,PTP4A1,NEK2,NCAPG,KIF15,CCNA1,KIF14,RASSF3,KIF11,RAD51,RAD51D,CENPU,RASSF10,STMN1,PRC1,KIF20A,CCNF,CCNB2,TOP2A
GO:0005694	CC	chromosome	4.51E-07	1.97E-05	1.29E-04	1.77E-04	33	NUSAP1,KIF18A,CENPA,HJURP,SPC25,RCC1,CDC45,FANCD2,SINHCAF,CENPM,CLTC,PLK1,

								BIRC5,SPC24,CENPH,CENPI,MTBP,POLQ,CDCA5,HMGB4,RAD21L1,NUF2,TRIM24,NEK2,NCAPG,BUB1,PARPBP,RAD51,RAD51D,CENPU,RFC3,ASXL2,TOP2A
GO:0044430	CC	cytoskeletal part	9.84E-07	3.65E-05	2.39E-04	3.87E-04	44	NUSAP1,KIF18A,SPEF2,TTK,ADD2,CDC45,CLTC,PLK1,KIF23,POC1A,CARMIL1,GTSE1,BIRC5,KRT87P,TPX2,RACGAP1,MYBPC1,KIF21B,HAUS6,MYO1D,KIF26A,DISC1,DLGAP5,DAB1,PTP4A1,NEK2,NCAPG,KIF15,KIF14,RASSF3,KRTAP5-7,KIF11,RAD51,RAD51D,KRT28,CENPU,RASSF10,STMN1,PRC1,KIF20A,CCNF,CCNB2,KRTAP1-5,TOP2A
GO:0008017	MF	microtubule binding	6.06E-06	2.40E-03	1.73E-02	4.63E-03	13	NUSAP1,KIF18A,PLK1,KIF23,BIRC5,RACGAP1,KIF21B,KIF26A,KIF15,KIF14,KIF11,PRC1,KIF20A
GO:0003777	MF	microtubule motor activity	8.30E-06	2.40E-03	1.73E-02	6.34E-03	08	KIF18A,KIF23,KIF21B,KIF26A,KIF15,KIF14,KIF11,KIF20A
GO:0015631	MF	tubulin binding	9.41E-06	2.40E-03	1.73E-02	7.19E-03	15	NUSAP1,KIF18A,PLK1,KIF23,BIRC5,RACGAP1,KIF21B,KIF26A,KIF15,KIF14,KIF11,RAD51D,STMN1,PRC1,KIF20A
GO:0008574	MF	ATP-dependent microtubule motor activity, plus-end-directed	6.32E-05	1.05E-02	7.57E-02	4.83E-02	04	KIF18A,KIF26A,KIF14,KIF11
GO:0003774	MF	motor activity	6.86E-05	1.05E-02	7.57E-02	5.24E-02	09	KIF18A,KIF23,KIF21B,MYO1D,KIF26A,KIF15,KIF14,KIF11,KIF20A
GO:1990939	MF	ATP-dependent microtubule motor activity	1.01E-04	1.14E-02	8.19E-02	7.71E-02	04	KIF18A,KIF26A,KIF14,KIF11
GO:0016887	MF	ATPase activity	1.04E-04	1.14E-02	8.19E-02	7.95E-02	17	KIF18A,KIF23,POLQ,ATP2A3,ATP2B2,KIF21B,MYO1D,KIF26A

								,DHX15,KIF15,KIF14,KIF11,RAD51,RAD51D,RFC3,KIF20A,TOP2A
GO:0043142	MF	single-stranded DNA-dependent ATPase activity	7.57E-04	6.64E-02	4.79E-01	5.79E-01	03	POLQ,RAD51,RFC3
GO:0030165	MF	PDZ domain binding	8.28E-04	6.64E-02	4.79E-01	6.33E-01	07	ARHGAP29,NLGN1,CASK,ATP2B2,KIF14,L1CAM,GRIK2
GO:0044877	MF	protein-containing complex binding	8.70E-04	6.64E-02	4.79E-01	6.64E-01	37	NUSAP1,DOK6,KIF18A,NRG1,CENPA,RBPJL,RCC1,ADD2,CDC45,CKS2,PLK1,KIF23,VWF,CARMIL1,BIRC5,CASK,CENPH,RACGAP1,POLQ,CDCA5,KIF21B,MYO1D,KIF26A,DISC1,TRIM24,YTHDF1,PRDM11,KIF15,KIF14,KIF11,RAD51,L1CAM,PRC1,KIF20A,TLE4,TOP2A,EPHB1

Note: Biological Process(BP), Cellular Component(CC) and Molecular Functions (MF).

Table 6. Topology table for up and down regulated genes.

Category	Node	Degree	Betweenness	Stress	Closeness	Clustering Coefficient
Up	SALL1	20	6.73E-03	599040	0.270852	0.026316
Up	BMP7	42	1.85E-02	2547692	0.241805	0
Up	FAM83F	2	5.08E-04	43304	0.20099	0
Up	SGCD	8	3.55E-03	808402	0.171417	0
Up	C1orf168	1	0.00E+00	0	1	0
Up	GRHL2	7	1.70E-03	324668	0.243088	0
Up	TDO2	18	3.51E-03	741118	0.221367	0
Up	SMR3B	11	2.91E-03	241848	0.209856	0
Up	SCRG1	1	0.00E+00	0	0.171291	0
Up	IL20RA	36	1.30E-02	3240444	0.233049	0
Up	FCN2	7	2.56E-03	197754	0.189402	0
Up	DPPA4	105	5.28E-02	8404518	0.278355	1.90E-04
Up	ATP4A	91	4.54E-02	4727962	0.263514	0
Up	GPR12	2	5.02E-04	50910	0.215258	0
Up	SDK2	11	1.32E-03	347148	0.206891	0
Up	SEPT14	9	3.56E-03	546322	0.189457	0
Up	RSPO3	5	2.03E-03	178044	0.172718	0
Up	SLC13A5	2	1.07E-04	16672	0.203829	0
Up	FGFR2	93	5.79E-02	6781948	0.291174	0.003175
Up	MGST1	7	1.66E-03	259370	0.209421	0

Up	FABP7	10	3.53E-03	348144	0.198368	0
Up	PON3	2	1.00E+00	2	1	0
Up	GAL	9	2.89E-03	341670	0.219065	0
Up	PAX6	49	2.00E-02	1487900	0.266617	0.002775
Up	SYPL2	12	3.97E-03	648624	0.216559	0
Up	LGI1	2	5.08E-04	71602	0.185225	0
Up	SLC15A1	64	2.63E-02	6462618	0.251196	0
Up	TMPRSS3	33	1.58E-02	3314720	0.24797	0
Up	ROR2	49	1.54E-02	2469916	0.257031	0
Up	DACT2	4	1.06E-03	191246	0.177576	0
Up	SMTNL2	16	4.38E-03	927394	0.241701	0
Up	KRT40	313	1.59E-01	22565808	0.304829	0
Up	FBN2	24	6.88E-03	1381044	0.220957	0
Up	NTF4	5	2.03E-03	147964	0.13475	0
Up	GBA3	2	5.08E-04	52696	0.148844	0
Up	SLIT1	2	5.08E-04	49644	0.176676	0
Up	OR7A5	1	0.00E+00	0	0.214321	0
Up	BBOX1	19	6.49E-03	440000	0.226003	0
Up	ART3	24	8.13E-03	976682	0.239993	0
Up	ID4	13	3.35E-03	782844	0.231447	0
Up	PLIN4	3	7.56E-05	37378	0.213461	0
Up	TRDN	37	1.31E-02	2095642	0.247674	0
Up	CLIC5	8	2.39E-03	360044	0.208391	0
Up	IRX1	1	0.00E+00	0	0.200969	0
Up	FCER1A	3	3.26E-05	5636	0.185557	0
Up	NGEF	21	7.48E-03	1282594	0.208005	0
Up	CCKBR	17	6.42E-03	1313620	0.225189	0
Up	ITLN1	11	3.22E-03	584478	0.223274	0
Up	S100A1	23	5.90E-03	824942	0.251533	0
Up	TMEM139	3	3.55E-03	258886	0.184289	0
Up	PMAIP1	14	4.74E-03	615246	0.230621	0
Up	GHRHR	1	0.00E+00	0	1	0
Up	XDH	2	5.08E-04	44858	0.200601	0
Up	TRIM29	104	6.13E-02	9041322	0.297553	0
Up	UGT2A2	1	0.00E+00	0	1	0
Up	SOX5	37	1.43E-02	1508376	0.275378	0
Up	S100A1	23	5.90E-03	824942	0.251533	0
Up	GALR1	1	0.00E+00	0	0.179707	0
Up	GAS1	3	1.01E-03	43314	0.226575	0
Up	PPP1R17	1	0.00E+00	0	0.214321	0
Up	IFNE	10	2.80E-03	416368	0.217901	0
Up	CELSR1	17	4.71E-03	588542	0.223388	0
Up	C4orf19	7	5.94E-04	75814	0.20404	0
Up	POMC	13	5.46E-03	738136	0.221941	0
Up	LRAT	3	0.00E+00	0	1	0
Up	KLK11	27	1.36E-02	2408872	0.236335	0

Up	F5	15	4.61E-03	881738	0.220265	0
Up	CNTNAP2	8	2.06E-03	345082	0.217348	0
Up	ENPP6	40	1.39E-02	3610524	0.233408	0
Up	GBP6	3	1.02E-03	53086	0.167077	0
Up	OBP2A	7	3.04E-03	662994	0.170129	0
Up	RBP4	15	4.72E-03	550068	0.226992	0
Up	PPP1R27	1	0.00E+00	0	0.166294	0
Up	ITIH2	16	5.09E-03	851246	0.219884	0
Up	NEURL3	1	0.00E+00	0	1	0
Up	CLIC5	8	2.39E-03	360044	0.208391	0
Up	PPP1R27	1	0.00E+00	0	0.166294	0
Up	RASL11B	6	1.08E-03	251748	0.233312	0
Up	KCNJ12	17	4.14E-03	1228964	0.207786	0
Up	AQP4	3	5.64E-04	54624	0.198508	0
Up	CD8B	2	5.08E-04	43304	0.20099	0
Up	P2RY2	4	9.10E-04	137648	0.233077	0
Up	IL22RA1	7	2.15E-03	456438	0.199181	0
Up	RNF207	4	6.33E-04	99124	0.213496	0
Up	ADAMTS18	5	7.80E-04	175548	0.216858	0
Up	NGFR	46	1.93E-02	2626710	0.258024	0
Up	KCNJ16	1	0.00E+00	0	1	0
Up	EFEMP1	56	3.53E-02	3383126	0.271749	0
Up	CHN2	13	3.42E-03	578204	0.234506	0
Up	GPHA2	21	6.38E-03	1083752	0.214718	0
Up	GHRHR	1	0.00E+00	0	1	0
Up	KRT40	313	1.59E-01	22565808	0.304829	0
Up	C6orf132	1	0.00E+00	0	0.195135	0
Up	NRAP	7	2.54E-03	723962	0.182437	0
Up	PHACTR2	16	5.85E-03	1042524	0.242609	0
Up	EVC2	86	4.01E-02	3002590	0.269961	0
Up	FOXL2	70	2.86E-02	8356260	0.257889	0
Up	FAM3B	9	5.47E-03	400338	0.24077	0
Up	DLK1	69	3.09E-02	5339202	0.257485	0
Up	BLID	12	2.22E-03	427182	0.219345	0
Up	CALB1	8	1.87E-03	281870	0.227635	0
Up	OGN	10	3.36E-03	588086	0.221218	0
Up	TF	59	3.30E-02	5546776	0.282609	0
Up	SMAD9	114	7.87E-02	8103704	0.303093	0
Up	SOX2	355	2.07E-01	37524600	0.318174	0
Up	KRT14	53	2.76E-02	6537242	0.277277	0
Up	DLGAP2	14	8.62E-03	607018	0.259128	0
Up	LRRC2	2	1.61E-05	2042	0.164853	0
Up	AMOT	111	6.81E-02	13889668	0.286702	0
Up	ZNF385C	7	2.12E-03	396460	0.198308	0
Up	CCK	2	5.08E-04	109288	0.183825	0
Up	HSD11B1	16	5.27E-03	800492	0.218251	0

Up	SGCD	8	3.55E-03	808402	0.171417	0
Up	PRSS8	1	0.00E+00	0	1	0
Up	C6orf118	5	1.11E-03	182260	0.220228	0
Up	LAD1	7	2.00E-03	282468	0.222492	0
Up	MYL10	3	3.70E-04	73004	0.215777	0
Up	CRISP2	5	2.02E-03	232114	0.218082	0
Up	IHH	2	5.08E-04	70496	0.159532	0
Up	MCOLN2	1	0.00E+00	0	1	0
Up	SLC39A12	70	3.22E-02	4986186	0.249178	0
Up	VIT	2	5.08E-04	69608	0.209376	0
Up	PPP1R17	1	0.00E+00	0	0.214321	0
Up	TSHB	15	6.40E-03	1264174	0.237848	0
Up	GADD45G	56	2.74E-02	5419566	0.271843	0
Up	FXYD3	7	1.80E-03	219094	0.20662	0
Up	LIX1	2	1.00E+00	2	1	0
Up	PRSS45	3	1.00E+00	6	1	0
Up	MFAP4	6	1.38E-03	246964	0.216631	0
Up	SPON1	3	1.02E-03	109098	0.214367	0
Up	NFIX	22	5.02E-03	1428620	0.232403	0
Up	COL8A2	18	6.18E-03	653142	0.221205	0
Up	GNRHR	1	0.00E+00	0	1	0
Up	SULT1C2	10	4.91E-03	344354	0.215375	0
Up	CA8	33	1.30E-02	1324240	0.262215	0
Up	CHRDL1	6	7.97E-04	108788	0.198089	0
Up	MEGF6	6	1.43E-03	100130	0.228083	0
Up	RPRM	9	3.81E-03	39669	0.211331	0
Up	OCA2	3	8.78E-05	7862	0.168788	0
Up	MPPED1	26	8.03E-03	1622472	0.222492	0
Up	CARTPT	3	1.00E+00	6	1	0
Up	CSH1	13	5.61E-03	475316	0.257014	0
Up	GALNT9	1	0.00E+00	0	0.189969	0
Up	OGN	10	3.36E-03	588086	0.221218	0
Up	FAM153B	14	5.40E-03	935250	0.2049	0
Up	GADD45G	56	2.74E-02	5419566	0.271843	0
Up	VSNL1	10	1.31E-03	162184	0.199151	0
Up	KISS1R	3	1.02E-03	144830	0.182319	0
Up	FGFR3	49	2.12E-02	2766462	0.268434	0
Up	WNT5A	22	7.80E-03	881282	0.206349	0
Up	CD8B	2	5.08E-04	43304	0.20099	0
Up	SFRP2	17	3.65E-03	988952	0.223769	0
Up	HSPB3	24	9.14E-03	1066592	0.24811	0
Up	PAX6	49	2.00E-02	1487900	0.266617	0.002775
Up	CYBRD1	21	7.61E-03	1486198	0.221255	0
Up	FXYD3	7	1.80E-03	219094	0.20662	0
Up	PGR	68	3.40E-02	5124700	0.28689	0
Up	HSD17B2	2	5.08E-04	61020	0.152911	0

Up	OPCML	11	9.23E-03	415810	0.253949	0
Up	RFX4	4	5.08E-04	68768	0.194519	0
Up	AGR3	14	3.65E-03	622642	0.231135	0
Up	SLIT1	2	5.08E-04	49644	0.176676	0
Up	SOX9	19	4.66E-03	1135172	0.228918	0
Up	CSHL1	3	1.02E-03	142558	0.195484	0
Up	CHST9	4	4.30E-04	57912	0.2136	0
Up	SULT1C2	10	4.91E-03	344354	0.215375	0
Up	DYDC2	1	0.00E+00	0	1	0
Up	SLIT1	4	1.52E-03	98748	0.177265	0
Up	SNTG2	14	4.17E-03	686616	0.201051	0
Up	ZNF750	1	0.00E+00	0	0.196381	0
Up	TAC4	3	1.00E+00	6	1	0
Up	PTER	14	4.59E-03	989784	0.223477	0
Up	CTRB1	2	1.00E+00	2	1	0
Up	COL21A1	2	0.00E+00	0	0	0
Up	ATF7IP2	4	7.03E-04	79612	0.215258	0
Up	NEFM	50	2.12E-02	4143078	0.272012	0
Up	S100A14	21	8.84E-03	986808	0.265288	0
Up	AOX1	6	1.58E-03	130420	0.202895	0
Up	KLRF1	5	6.70E-04	77804	0.192503	0
Up	CCDC68	13	5.14E-03	436104	0.208269	0
Up	CNKSR3	9	2.74E-03	263976	0.196244	0
Up	CRYM	4	5.65E-04	44096	0.209666	0
Up	MYZAP	1	0.00E+00	0	0.213484	0
Up	PDE6A	4	1.43E-04	8434	0.238597	0
Up	INHBA	8	2.57E-03	734598	0.176407	0
Up	CNTNAP3	33	1.34E-02	1433542	0.235939	0
Up	FAM153C	2	5.08E-04	85808	0.170078	0
Up	C6orf141	74	3.72E-02	9207512	0.260292	0
Up	RUNX1	79	3.50E-02	4622022	0.286015	0
Up	TRIM71	17	6.52E-03	713150	0.220623	0
Up	PNMT	3	5.88E-04	55878	0.215493	0
Up	PRSS50	25	9.88E-03	1981826	0.256663	0
Up	SLC47A1	27	1.28E-02	765214	0.222832	0
Up	PHACTR3	29	1.17E-02	1887264	0.234506	0
Up	IL20RA	36	1.30E-02	3240444	0.233049	0
Up	LEFTY2	3	1.02E-03	95398	0.147528	0
Up	RHBDL3	4	1.52E-03	323400	0.165874	0
Up	GH1	85	3.79E-02	5815610	0.26716	0
Up	STRA6	2	5.08E-04	57684	0.184652	0
Up	FRAS1	33	1.76E-02	1896950	0.244978	0
Up	CT45A1	3	1.00E+00	6	1	0
Up	FGFR3	49	2.12E-02	2766462	0.268434	0
Up	EYA4	26	6.49E-03	1286994	0.239075	0
Up	AMZ1	12	3.35E-03	644006	0.193934	0

Up	CACNA1A	98	5.07E-02	4392082	0.272841	0
Up	CA12	13	3.67E-03	625692	0.218106	0
Up	GAS7	28	9.64E-03	1499288	0.218894	0
Up	HPGD	1	0.00E+00	0	0.214321	0
Up	FMO5	5	9.24E-04	141874	0.217516	0
Up	SCGB1D2	9	2.67E-03	252442	0.209376	0
Up	PLIN5	6	2.54E-03	488350	0.178558	0
Up	KCNA1	7	2.68E-03	237802	0.195435	0
Up	ALDOB	28	1.11E-02	1644614	0.253084	0
Up	RHOD	19	7.80E-03	1084918	0.189786	0
Up	GJB7	51	2.16E-02	2788870	0.243992	0
Up	ALDH1A3	9	2.42E-03	378026	0.220327	0
Up	EYA2	88	4.15E-02	6323538	0.281237	0
Up	KRT19	59	2.60E-02	7305954	0.276926	0
Up	LY6H	3	5.57E-04	60178	0.176092	0
Up	MYBPHL	7	2.32E-03	246776	0.198308	0
Up	RASGRP1	4	3.51E-04	53352	0.195019	0
Up	GH2	2	8.82E-06	644	0.197325	0
Up	FERMT1	15	4.58E-03	653358	0.227097	0
Up	ASXL3	2	5.08E-04	44514	0.172627	0
Up	TEPP	1	0.00E+00	0	0.209354	0
Up	PDZRN3	11	4.13E-03	858088	0.195387	0
Up	KCNS2	1	0.00E+00	0	0.148569	0
Up	VANGL2	33	1.46E-02	1934604	0.232321	0
Up	PRDM16	6	1.44E-03	306010	0.223972	0
Up	PLAC1	20	6.67E-03	1205836	0.224957	0
Up	SH3BP4	43	1.67E-02	2668100	0.262618	0
Up	COL4A6	9	4.29E-03	288964	0.222241	0
Up	NEUROD1	15	3.84E-03	758734	0.219016	0
Up	IL6R	17	6.73E-03	876290	0.223845	0
Up	FSHB	2	1.00E+00	2	1	0
Up	CSH2	13	5.61E-03	475316	0.257014	0
Up	CNTNAP3B	16	7.15E-03	582922	0.190741	0
Up	CHRDL1	6	7.97E-04	108788	0.198089	0
Up	NWD1	1	0.00E+00	0	0.175339	0
Up	WWC2	18	3.48E-03	975000	0.230041	0
Up	APOD	50	3.56E-02	2147634	0.271412	0
Up	GJB7	51	2.16E-02	2788870	0.243992	0
Up	RXRG	32	1.14E-02	2426724	0.228268	0
Up	RNF175	2	2.24E-05	1134	0.179355	0
Up	IGSF11	2	0.00E+00	0	0	0
Up	SCN2B	86	3.56E-02	7895890	0.255896	0
Up	OVOL2	1	0.00E+00	0	0.216108	0
Up	CCDC158	12	2.91E-03	441576	0.225705	0
Up	ASXL3	2	5.08E-04	44514	0.172627	0
Up	CYP4B1	2	1.00E+00	2	1	0

Up	DLGAP2	14	8.62E-03	607018	0.259128	0
Up	TPH1	7	1.87E-03	396120	0.227635	0
Up	AOX1	6	1.58E-03	130420	0.202895	0
Up	SLC39A8	26	1.05E-02	1259100	0.220179	0
Up	S100B	33	1.07E-02	1384834	0.252922	0
Up	SLPI	7	4.10E-03	388202	0.209143	0
Up	PGR	68	3.40E-02	5124700	0.28689	0
Up	SSTR2	7	2.14E-03	321652	0.229425	0
Up	FLRT2	3	0.00E+00	0	1	0
Up	C2orf71	5	2.03E-03	289652	0.182386	0
Up	COL13A1	2	0.00E+00	0	0	0
Up	CACNA2D3	1	0.00E+00	0	1	0
Up	NR0B1	15	5.59E-03	309134	0.247425	0
Up	CALB2	4	1.64E-05	1926	0.187706	0
Up	STMN2	27	1.22E-02	1546160	0.247254	0
Up	SCGB1D1	71	4.35E-02	6754916	0.254063	0
Up	FGF1	27	1.23E-02	832698	0.262916	0
Up	KCNA1	7	2.68E-03	237802	0.195435	0
Up	FOXL2	70	2.86E-02	8356260	0.257889	0
Up	MAP3K15	7	1.18E-03	196726	0.213172	0
Up	MKX	10	2.04E-03	492958	0.220302	0
Up	TXLNB	43	1.53E-02	4523946	0.233769	0
Up	T	1	0.00E+00	0	1	0
Up	ERBB4	84	3.50E-02	8327424	0.269131	0
Up	PPP1R1B	8	1.69E-03	176268	0.187885	0
Up	PCSK2	2	0.00E+00	0	0	0
Up	DMKN	7	2.21E-03	390378	0.236123	0
Up	TGFBR3	22	9.08E-03	805594	0.23413	0
Up	MN1	3	4.47E-05	9412	0.20941	0
Up	SLIT1	2	5.08E-04	49644	0.176676	0
Up	KCNG1	5	1.09E-03	200078	0.194567	0
Up	MEGF11	2	5.16E-05	7530	0.192871	0
Up	PLA2G4A	35	1.68E-02	3125428	0.264079	0
Up	ERP27	5	6.80E-04	96440	0.20941	0
Up	GPR37L1	1	0.00E+00	0	1	0
Up	SMR3B	11	2.91E-03	241848	0.209856	0
Up	CLIC3	9	3.25E-03	417244	0.21813	0
Up	SYT12	16	5.27E-03	975528	0.226783	0
Up	CUBN	10	2.40E-03	351968	0.206793	0
Up	PNMA3	5	7.32E-04	128516	0.189685	0
Up	FAM198A	3	9.84E-05	8632	0.182912	0
Up	FAM124A	29	1.00E-02	2057940	0.226796	0
Up	CPLX3	17	7.35E-03	422424	0.250732	0
Up	ADARB2	9	2.11E-03	368998	0.226171	0
Up	DMKN	7	2.21E-03	390378	0.236123	0
Up	BANK1	4	1.46E-04	22828	0.217336	0

Up	HIF3A	3	6.24E-04	67108	0.19472	0
Up	CNTNAP3	33	1.34E-02	1433542	0.235939	0
Up	CCNJL	39	1.50E-02	3448762	0.260533	0
Up	ADAM32	21	6.74E-03	1279704	0.223185	0
Up	PHYHD1	3	7.04E-04	88272	0.214905	0
Up	LAMA1	31	1.32E-02	3129918	0.248298	0
Up	DNAI2	12	3.01E-03	467486	0.2	0
Down	TRIM24	66	2.39E-02	7566812	0.292625	0
Down	KLHL13	30	8.55E-03	2607752	0.279499	0
Down	MAGEB2	46	1.08E-02	4326748	0.268579	0
Down	PPBP	22	7.30E-03	2718118	0.22063	0
Down	TTK	77	3.23E-02	12267556	0.31332	7.21E-04
Down	FAM71D	3	6.39E-05	15292	0.197561	0
Down	TOP2A	102	4.15E-02	13126864	0.323325	0.002912
Down	KIF18A	27	9.22E-03	1047074	0.272947	0.002849
Down	TPX2	45	1.33E-02	5212220	0.283324	0.00303
Down	POC1A	3	2.10E-04	38988	0.263713	0
Down	ARHGAP29	14	2.99E-03	1435976	0.254092	0
Down	NRG1	66	2.98E-02	8652224	0.277873	0
Down	PLCD4	3	1.07E-03	235394	0.224878	0
Down	CADPS2	15	4.01E-03	1430040	0.22412	0
Down	CCNF	13	2.36E-03	321772	0.266733	0
Down	GTSE1	141	5.29E-02	7085030	0.337622	0.008713
Down	TRHDE	15	4.61E-03	1973410	0.23257	0
Down	SOX4	27	1.24E-02	7096428	0.258212	0
Down	DLEU1	21	6.78E-03	2289500	0.253469	0
Down	PLK1	276	1.64E-01	39488508	0.35244	0.001016
Down	HAUS6	53	1.32E-02	7866150	0.288256	0
Down	BUB1	95	4.02E-02	4659062	0.318162	0.008649
Down	SERINC2	8	2.18E-03	1257316	0.206187	0
Down	KIF14	62	1.75E-02	10376292	0.284688	0
Down	VWA1	14	4.26E-03	366250	0.21001	0
Down	PTTG2	1	0.00E+00	0	0.224824	0
Down	SEL1L	58	2.60E-02	9557720	0.270569	0
Down	NUSAP1	35	1.32E-02	2253376	0.300363	0.008913
Down	CELA3A	12	3.00E-03	434718	0.234812	0
Down	DOK6	6	7.47E-04	300368	0.234664	0
Down	NRG1	66	2.98E-02	8652224	0.277873	0
Down	DHX15	208	9.39E-02	39966314	0.320847	0
Down	NEK2	52	1.53E-02	5682386	0.277397	0
Down	TAS2R7	14	5.78E-03	635150	0.247921	0.010989
Down	SPC24	41	1.22E-02	1425412	0.291526	0.039024
Down	HMMR	44	1.23E-02	3764124	0.283002	0.003171
Down	PTP4A1	53	2.21E-02	5500792	0.256435	0
Down	DAZAP2	99	4.35E-02	9779248	0.28839	0.003651
Down	CDCA5	55	1.97E-02	1719302	0.3	0.002903

Down	ANTXR2	12	3.86E-03	876552	0.218842	0
Down	FBXL5	41	1.34E-02	1287978	0.299253	0.006748
Down	PLA2G2D	1	0.00E+00	0	0.206084	0
Down	DAB1	67	2.85E-02	5666990	0.279457	0.007689
Down	RBPJL	2	5.37E-04	117698	0.224851	0
Down	FANCA	86	2.87E-02	13664846	0.284167	0
Down	NLGN1	11	3.82E-03	881670	0.215525	0
Down	POU3F2	10	2.82E-03	567692	0.225258	0
Down	PPP4R4	3	5.68E-04	157816	0.205041	0
Down	GPR6	2	5.37E-04	60688	0.210938	0
Down	PAX4	3	1.07E-03	234714	0.21372	0
Down	ASXL2	18	6.05E-03	1686090	0.242263	0
Down	IGFL3	1	0.00E+00	0	1	0
Down	GRIK2	13	4.44E-03	430606	0.243976	0.025641
Down	NCAPG	52	1.62E-02	5737472	0.285517	0
Down	SLC19A1	3	5.43E-04	132382	0.188974	0
Down	EPHB1	14	3.21E-03	1135208	0.242942	0
Down	INHBE	15	7.30E-03	1600282	0.257445	0
Down	FIBCD1	1	0.00E+00	0	1	0
Down	RGS5	7	2.09E-03	462166	0.237446	0
Down	CCNB2	36	8.70E-03	3365996	0.275673	0.009524
Down	ADAMTSL1	7	2.82E-03	352088	0.208506	0
Down	FAM83A	7	1.40E-03	115518	0.279038	0.047619
Down	LRFN1	20	9.73E-03	1618782	0.237416	0
Down	PKLR	19	4.89E-03	591642	0.267308	0.005848
Down	SLC25A15	15	5.26E-03	1022014	0.258069	0
Down	PRC1	29	6.84E-03	1142838	0.294452	0.031339
Down	AMIGO2	6	1.08E-03	410596	0.178235	0
Down	KIF11	134	5.97E-02	17181634	0.31282	0
Down	G2E3	6	1.27E-03	455226	0.218061	0
Down	OR6T1	1	0.00E+00	0	0.225613	0
Down	POLQ	1	0.00E+00	0	1	0
Down	GRIK2	13	4.44E-03	430606	0.243976	0.025641
Down	RASSF3	28	7.78E-03	3540288	0.258176	0
Down	PBK	107	4.20E-02	12464178	0.300775	0
Down	CENPA	88	3.07E-02	4765456	0.300799	0.006293
Down	LYNX1	1	0.00E+00	0	0.189426	0
Down	ASPDH	10	3.69E-03	974500	0.235242	0
Down	DLGAP5	23	7.53E-03	2047406	0.302705	0.019763
Down	KIF20A	38	9.99E-03	3761204	0.277108	0
Down	CNKSR2	10	2.74E-03	698616	0.245083	0
Down	GOLGA6A	1	0.00E+00	0	0.194204	0
Down	B4GALT6	4	1.00E+00	12	1	0
Down	KRT28	1	0.00E+00	0	1	0
Down	CDKN3	29	1.03E-02	1383840	0.274414	0.002849
Down	DCAF4L2	7	1.14E-03	263466	0.229603	0

Down	CALHM1	2	0.00E+00	0	0	0
Down	ADD2	17	5.29E-03	1384972	0.251723	0
Down	CDKN3	29	1.03E-02	1383840	0.274414	0.002849
Down	RYR2	28	8.08E-03	2614302	0.258642	0
Down	C20orf203	2	1.00E+00	2	1	0
Down	YTHDF1	61	2.13E-02	7937228	0.298366	0
Down	FOXM1	80	2.74E-02	8443400	0.298749	0.002331
Down	GJA4	3	1.07E-03	514922	0.181296	0
Down	PF4	11	5.89E-03	2016954	0.188248	0.027778
Down	ANKFY1	41	1.24E-02	5747558	0.26362	0
Down	AKAP3	10	3.76E-03	892188	0.240046	0
Down	DCAF12L2	11	3.30E-03	771012	0.231472	0
Down	BDNF	11	4.67E-03	1264752	0.242373	0
Down	BIRC5	61	2.10E-02	6990168	0.290549	0
Down	SLC39A5	45	1.94E-02	2952184	0.228407	0
Down	GLYATL3	1	0.00E+00	0	0.190715	0
Down	L1CAM	24	6.90E-03	2825930	0.267884	0
Down	SV2C	7	2.26E-03	545018	0.20922	0
Down	KIF15	27	7.88E-03	1833408	0.267865	0
Down	FANCD2	74	3.14E-02	6501308	0.312978	0.004813
Down	RACGAP1	89	3.18E-02	7802394	0.304263	0.005107
Down	FAM20C	12	4.87E-03	828322	0.200852	0
Down	NUF2	44	1.42E-02	2296298	0.301725	3.38E-02
Down	QSOX1	15	4.90E-03	1223750	0.241087	0
Down	SLITRK6	1	0.00E+00	0	0.206759	0
Down	FAM91A1	23	7.07E-03	2542312	0.2639	0
Down	GPR161	16	8.15E-03	864880	0.246363	0.008333
Down	SCEL	14	5.37E-03	1611186	0.249883	0
Down	CXCR3	6	2.15E-03	733476	0.158492	0.066667
Down	RFT1	21	6.86E-03	2273166	0.225517	0
Down	PSG5	4	1.24E-03	315510	0.199775	0
Down	RAD51	120	4.54E-02	8161594	0.307324	0.001883
Down	ADAMTSL1	7	2.82E-03	352088	0.208506	0
Down	HMGB4	3	6.06E-04	146328	0.226988	0
Down	TROAP	33	1.24E-02	1251972	0.293872	0.006452
Down	RASSF10	38	1.21E-02	3884098	0.259851	0
Down	ZC3H12D	1	0.00E+00	0	1	0
Down	HJURP	21	4.31E-03	873270	0.271041	0.035088
Down	XKR6	1	0.00E+00	0	0.225613	0
Down	UNC5D	1	0.00E+00	0	0.201809	0
Down	C1orf189	2	1.00E+00	2	1	0
Down	PSTPIP2	8	1.87E-03	472358	0.247427	0
Down	XKR6	1	0.00E+00	0	0.225613	0
Down	EXD1	1	0.00E+00	0	1	0
Down	KIF21B	30	9.70E-03	2431650	0.256842	0
Down	GRIP1	53	1.84E-02	6233398	0.270372	0.001569

Down	POU3F3	3	5.62E-04	84710	0.208378	0
Down	CPN1	6	1.71E-03	328228	0.22172	0
Down	SPC25	35	6.24E-03	630312	0.27207	0.052101
Down	C12ORF66	4	1.61E-03	77742	0.201711	0
Down	MELK	23	6.63E-03	1668988	0.250673	0
Down	TLE4	28	9.56E-03	2438214	0.252507	0
Down	MND1	7	2.70E-03	788252	0.224986	0
Down	ZMAT3	24	7.69E-03	2968830	0.271931	0
Down	ATP2A3	53	2.39E-02	6619182	0.256171	0
Down	GRIP1	53	1.84E-02	6233398	0.270372	0.001569
Down	CENPM	15	2.31E-03	174586	0.25524	0.114286
Down	GRIK2	13	4.44E-03	430606	0.243976	0.025641
Down	ZSCAN2	1	0.00E+00	0	1	0
Down	GPR26	1	0.00E+00	0	1	0
Down	NXPH4	4	1.61E-03	1133406	0.17246	0
Down	UNC5D	1	0.00E+00	0	0.201809	0
Down	RNF215	1	0.00E+00	0	1	0
Down	STMN1	65	2.51E-02	7293058	0.286065	4.81E-04
Down	ARHGAP20	2	1.00E-05	3872	0.219564	0
Down	FAM72D	1	0.00E+00	0	0.225613	0
Down	HTR3D	4	7.06E-04	153078	0.180646	0
Down	ATP2B2	118	5.57E-02	10270314	0.275124	2.90E-04
Down	CCNG2	11	3.40E-03	744714	0.210806	0
Down	RCC1	102	3.87E-02	10546556	0.314165	0.001941
Down	NPVF	3	3.46E-05	17228	0.217653	0
Down	KIF23	62	2.33E-02	4491558	0.306515	0.015819
Down	PSG11	4	1.12E-03	136444	0.188324	0
Down	PRR11	95	3.70E-02	16341014	0.259923	0
Down	LCE1B	22	1.04E-02	1365886	0.209408	0
Down	CCNA1	83	2.81E-02	8701494	0.2972	2.47E-03
Down	TLE4	28	9.56E-03	2438214	0.252507	0
Down	HIGD1B	3	1.00E+00	6	1	0
Down	OXR1	10	1.57E-03	396854	0.225914	0
Down	DEFA3	1	0.00E+00	0	0.184876	0
Down	DISC1	138	5.45E-02	23062588	0.288949	0
Down	CLTC	333	1.73E-01	59969246	0.345833	0.001483
Down	MTBP	7	9.00E-04	308238	0.25191	0
Down	COLEC10	14	5.78E-03	496564	0.230569	0
Down	PNPLA7	1	0.00E+00	0	1	0
Down	TLE4	28	9.56E-03	2438214	0.252507	0
Down	MYO1D	58	2.01E-02	6943106	0.297723	0
Down	CXCR2	9	2.70E-03	571596	0.206862	0
Down	POU3F4	3	3.10E-04	10712	0.214335	0
Down	KIF26A	3	5.58E-04	216568	0.213757	0
Down	VWF	20	6.90E-03	3379006	0.226877	0
Down	ZSCAN12	40	1.72E-02	4511560	0.249014	0

Down	OMP	1	0.00E+00	0	1	0
Down	SPINK5	1	0.00E+00	0	0.200236	0
Down	MYBPC1	24	1.02E-02	2208902	0.223047	0
Down	RFC3	48	1.19E-02	6744340	0.286615	0
Down	KIAA1958	14	4.06E-03	1021098	0.246641	0
Down	TMEM40	1	0.00E+00	0	1	0
Down	CTAG1A	2	5.37E-04	160340	0.182031	0
Down	CHRNA7	5	1.20E-03	315000	0.238785	0
Down	PARD6G	21	6.07E-03	2092448	0.251349	0
Down	ZNF160	4	1.00E+00	12	1	0
Down	CCL3L3	1	1.00E+00	6	1	0
Down	CKS2	36	8.66E-03	1293372	0.279227	2.50E-02
Down	HSD3B2	19	6.19E-03	434014	0.258678	0.005848
Down	RAD21L1	1	0.00E+00	0	0.198223	0
Down	HS3ST2	11	4.28E-03	1134682	0.226849	0
Down	GRM5	10	2.07E-03	756604	0.225846	0
Down	SPEF2	3	1.07E-03	311542	0.166704	0
Down	KIR3DX1	3	5.37E-04	151612	0.229872	0
Down	EYS	1	0.00E+00	0	0.217653	0
Down	CENPH	38	1.29E-02	1191544	0.293478	0.022222
Down	C3orf52	11	3.74E-03	897310	0.232454	0
Down	UNC13A	7	2.18E-03	1007070	0.215862	0
Down	RUFY4	3	5.58E-04	112984	0.219927	0
Down	GPR17	1	0.00E+00	0	1	0
Down	RAD21L1	1	0.00E+00	0	0.198223	0
Down	CASK	79	3.54E-02	8491074	0.295926	0
Down	OXR1	10	1.57E-03	396854	0.225914	0

Table 7. miRNA-target gene interaction table.

Regulation	target Genes	Degree	Top MicroRNA	Regulation	target Genes	Degree	Top MicroRNA
Up	SALL1	53	hsa-mir-548b-3p	Down	TRIM24	21	hsa-mir-4477a
Up	NKX2-2	20	hsa-mir-6829-3p	Down	KLHL13	1	hsa-mir-218-5p
Up	PTCHD1	60	hsa-mir-548a-3p	Down	MAGEB2	2	hsa-mir-98-5p
Up	BMP7	22	hsa-mir-6890-3p	Down	C3orf80	10	hsa-mir-3153
Up	TMEM154	49	hsa-mir-3187-3p	Down	PPBP	1	hsa-mir-335-5p
Up	SERTM1	13	hsa-mir-3924	Down	TTK	7	hsa-mir-376a-3p
Up	FAM83F	94	hsa-mir-612	Down	TOP2A	17	hsa-mir-708-5p
Up	SGCD	41	hsa-mir-4761-5p	Down	KIF18A	2	hsa-mir-192-5p
Up	C1orf168	2	hsa-mir-148b-3p	Down	TPX2	1	hsa-mir-193b-3p
Up	GRHL2	1	hsa-mir-26b-5p	Down	POC1A	69	hsa-mir-3605-5p
Up	SMR3B	1	hsa-mir-495-3p	Down	ARHGAP29	25	hsa-mir-6814-5p
Up	SCRG1	33	hsa-mir-1304-3p	Down	NRG1	1	hsa-mir-124-3p
Up	IL20RA	1	hsa-mir-26b-5p	Down	CADPS2	4	hsa-mir-30c-5p
Up	FCN2	8	hsa-mir-8064	Down	ACER2	46	hsa-let-7d-5p
Up	DPPA4	1	hsa-mir-26b-5p	Down	CCNF	102	hsa-mir-324-3p

Up	SPTLC3	32	hsa-mir-206	Down	GTSE1	2	hsa-mir-484
Up	ATP4A	1	hsa-mir-1289	Down	SOX4	160	hsa-mir-519a-3p
Up	SDK2	33	hsa-mir-644a	Down	DLEU1	31	hsa-mir-616-5p
Up	RSPO3	1	hsa-mir-335-5p	Down	PLK1	17	hsa-mir-3120-3p
Up	FGFR2	5	hsa-mir-19b-1-5p	Down	HAUS6	14	hsa-mir-5582-3p
Up	IQCA1	1	hsa-mir-193b-3p	Down	BUB1	67	hsa-mir-497-3p
Up	MGST1	2	hsa-mir-652-3p	Down	SERINC2	1	hsa-mir-335-5p
Up	FABP7	7	hsa-mir-6823-3p	Down	KIF14	14	hsa-mir-3133
Up	PON3	1	hsa-mir-335-5p	Down	VWA1	30	hsa-mir-592
Up	GAL	8	hsa-mir-4671-5p	Down	FRMPD3	18	hsa-mir-6511a-3p
Up	PAX6	23	hsa-mir-599	Down	SEL1L	12	hsa-mir-548as-3p
Up	SYPL2	1	hsa-mir-615-3p	Down	NUSAP1	1	hsa-let-7b-5p
Up	SLC15A1	19	hsa-mir-7977	Down	DOK6	23	hsa-mir-3679-3p
Up	TMPRSS3	2	hsa-mir-204-5p	Down	NRG1	1	hsa-mir-124-3p
Up	ROR2	3	hsa-mir-451a	Down	DHX15	7	hsa-mir-221-3p
Up	DACT2	2	hsa-mir-128-3p	Down	NEK2	2	hsa-mir-92a-3p
Up	SMTNL2	43	hsa-mir-3652	Down	TAS2R7	1	hsa-mir-142-3p
Up	FBN2	16	hsa-mir-23c	Down	SPC24	26	hsa-mir-3664-5p
Up	NTF4	1	hsa-mir-124-3p	Down	HMMR	5	hsa-mir-106b-5p
Up	SRL	2	hsa-mir-18a-3p	Down	PTP4A1	132	hsa-mir-186-5p
Up	SLIT1	33	hsa-mir-5787	Down	DAZAP2	109	hsa-mir-511-5p
Up	BBOX1	4	hsa-mir-4477a	Down	CDCA5	4	hsa-mir-331-3p
Up	ART3	2	hsa-mir-122-5p	Down	ANTXR2	60	hsa-mir-3065-5p
Up	NRK	1	hsa-mir-335-5p	Down	FBXL5	31	hsa-mir-4731-3p
Up	ID4	91	hsa-mir-4311	Down	PLA2G2D	20	hsa-mir-548au-3p
Up	PLIN4	1	hsa-mir-335-5p	Down	RBPJL	25	hsa-mir-6731-5p
Up	TRDN	17	hsa-mir-501-3p	Down	CENPI	2	hsa-mir-192-5p
Up	CLIC5	54	hsa-mir-4480	Down	NLGN1	8	hsa-mir-670-3p
Up	BTBD17	1	hsa-mir-335-5p	Down	POU3F2	19	hsa-mir-6781-5p
Up	CCKBR	3	hsa-mir-148a-3p	Down	PAX4	8	hsa-mir-4783-5p
Up	S100A1	4	hsa-mir-138-5p	Down	ASXL2	48	hsa-mir-5002-5p
Up	CBLN2	6	hsa-mir-6819-3p	Down	GRIK2	1	hsa-mir-26b-5p
Up	PMAIP1	128	hsa-mir-411-3p	Down	NCAPG	20	hsa-mir-664b-3p
Up	XDH	1	hsa-mir-26b-5p	Down	SLC19A1	9	hsa-mir-4422
Up	TRIM29	18	hsa-mir-1268a	Down	EPHB1	18	hsa-mir-548aw
Up	UGT2A2	9	hsa-mir-548u	Down	INHBE	2	hsa-mir-98-5p
Up	SOX5	38	hsa-mir-555	Down	FIBCD1	12	hsa-mir-6717-5p
Up	S100A1	4	hsa-mir-132-3p	Down	RGS5	42	hsa-mir-4255
Up	GALR1	8	hsa-mir-130b-5p	Down	GUCA2B	1	hsa-mir-335-5p
Up	GAS1	33	hsa-mir-449b-5p	Down	CCNB2	6	hsa-let-7f-5p
Up	PPP1R17	1	hsa-mir-335-5p	Down	ADAMTSL1	2	hsa-mir-25-3p
Up	IFNE	16	hsa-mir-4786-5p	Down	FAM83A	5	hsa-mir-5090
Up	CELSR1	2	hsa-mir-92a-3p	Down	LRFN1	18	hsa-mir-6887-3p
Up	C4orf19	5	hsa-mir-1273f	Down	SLC25A15	20	hsa-mir-6720-3p
Up	POMC	2	hsa-mir-488-3p	Down	GABRQ	1	hsa-mir-335-5p
Up	SLC6A14	1	hsa-mir-26b-5p	Down	PRC1	17	hsa-mir-3977

Up	LRAT	47	hsa-mir-4770	Down	AMIGO2	2	hsa-mir-155-5p
Up	SPTLC3	32	hsa-mir-6514-5p	Down	CDC45	2	hsa-mir-575
Up	KLK11	1	hsa-mir-335-5p	Down	PRDM11	1	hsa-mir-335-5p
Up	F5	4	hsa-mir-634	Down	CYP27B1	12	hsa-mir-1178-5p
Up	CNTNAP2	14	hsa-mir-571	Down	KIF11	15	hsa-mir-4495
Up	PI16	2	hsa-mir-1-1	Down	G2E3	54	hsa-mir-520g-3p
Up	ENPP6	24	hsa-mir-6077	Down	POLQ	86	hsa-mir-664a-5p
Up	GBP6	24	hsa-mir-548j-3p	Down	GRIK2	1	hsa-mir-26b-5p
Up	NEURL3	1	hsa-mir-335-5p	Down	RASSF3	3	hsa-mir-20a-3p
Up	CLIC5	54	hsa-mir-4301	Down	PBK	4	hsa-mir-4699-3p
Up	RASL11B	7	hsa-mir-3137	Down	CENPA	23	hsa-mir-4719
Up	KCNJ12	24	hsa-mir-770-5p	Down	LYNX1	1	hsa-mir-744-5p
Up	AQP4	1	hsa-mir-320a	Down	EVX2	9	hsa-mir-33b-3p
Up	P2RY2	16	hsa-mir-4433b-3p	Down	KRTAP5-7	1	hsa-mir-26b-5p
Up	IL22RA1	1	hsa-mir-26b-5p	Down	DLGAP5	21	hsa-mir-520e
Up	RNF207	10	hsa-mir-3190-5p	Down	EDN1	57	hsa-mir-379-5p
Up	ADAMTS18	16	hsa-mir-588	Down	KIF20A	5	hsa-mir-23a-3p
Up	NGFR	23	hsa-mir-6732-5p	Down	CNKSR2	10	hsa-mir-6808-3p
Up	KCNJ16	1	hsa-mir-148b-3p	Down	GOLGA6A	4	hsa-mir-450b-5p
Up	GPHA2	24	hsa-mir-1180-5p	Down	B4GALT6	19	hsa-mir-1248
Up	C6orf132	48	hsa-mir-4638-5p	Down	ARHGAP42	22	hsa-mir-6131
Up	PHACTR2	47	hsa-mir-590-3p	Down	KRT28	1	hsa-mir-1229-3p
Up	EVC2	18	hsa-mir-6873-3p	Down	CDKN3	18	hsa-mir-4695-3p
Up	FOXL2	10	hsa-mir-1253	Down	DCAF4L2	2	hsa-mir-494-3p
Up	FAM3B	1	hsa-mir-335-5p				
Up	DLK1	1	hsa-mir-15a-5p				
Up	BLID	1	hsa-mir-335-5p				
Up	UGT2A1	10	hsa-mir-4762-5p				
Up	OGN	1	hsa-mir-526b-5p				
Up	TF	2	hsa-mir-19a-3p				
Up	SMAD9	45	hsa-mir-4419b				
Up	LINC00598	209	hsa-mir-890				
Up	SOX2	15	hsa-mir-126-3p				
Up	KLK12	2	hsa-mir-1-3p				
Up	KRT14	2	hsa-mir-124-3p				
Up	DLGAP2	8	hsa-mir-20a-3p				
Up	LRRC2	46	hsa-mir-3914				
Up	AMOT	23	hsa-mir-500a-5p				
Up	LINC00598	209	hsa-mir-518a-5p				
Up	HSD11B1	2	hsa-mir-26b-5p				
Up	SGCD	41	hsa-mir-4698				
Up	PRSS8	1	hsa-mir-335-5p				
Up	C6orf118	2	hsa-mir-1-1				
Up	CRISP2	2	hsa-mir-27a-3p				
Up	IHH	22	hsa-mir-6839-5p				
Up	MCOLN2	18	hsa-mir-6731-3p				

Up	SLC39A12	1	hsa-mir-335-5p
Up	VIT	1	hsa-mir-124-3p
Up	PRRX2	1	hsa-mir-124-3p
Up	PPP1R17	1	hsa-mir-335-5p
Up	TSHB	1	hsa-mir-26b-5p
Up	AGR2	2	hsa-mir-197-3p
Up	KBTBD12	39	hsa-mir-4675
Up	GADD45G	1	hsa-mir-181a-5p
Up	LIX1	3	hsa-mir-4668-5p
Up	PRSS45	8	hsa-mir-765
Up	MFAP4	1	hsa-mir-124-3p
Up	ESPNL	1	hsa-mir-375
Up	NFIX	67	hsa-mir-4488
Up	EVC2	18	hsa-mir-1236-3p
Up	COL8A2	2	hsa-mir-124-3p
Up	GNRHR	4	hsa-mir-1-3p
Up	SULT1C2	2	hsa-mir-7-5p
Up	CA8	54	hsa-mir-646
Up	CHRDL1	54	hsa-mir-583
Up	RPRM	32	hsa-mir-939-5p
Up	OCA2	12	hsa-mir-1299
Up	MPPED1	15	hsa-mir-516a-3p
Up	OGN	1	hsa-mir-526b-5p
Up	GADD45G	1	hsa-mir-181a-5p
Up	VSNL1	25	hsa-mir-6873-5p
Up	FGFR3	9	hsa-mir-425-5p
Up	WNT5A	29	hsa-mir-4680-3p
Up	SFRP2	2	hsa-mir-218-5p
Up	HSPB3	1	hsa-mir-335-5p
Up	PAX6	23	hsa-mir-223-3p
Up	CYBRD1	42	hsa-mir-4517
Up	PGR	2	hsa-mir-181a-5p
Up	HSD17B2	2	hsa-mir-124-3p
Up	OPCML	9	hsa-mir-3149
Up	AGR3	1	hsa-mir-335-5p
Up	SLIT1	33	hsa-mir-4726-5p
Up	SOX9	30	hsa-mir-1323
Up	CSHL1	1	hsa-mir-16-5p
Up	CHST9	2	hsa-mir-92a-3p
Up	NRK	1	hsa-mir-335-5p
Up	SULT1C2	2	hsa-mir-7-5p
Up	SULT1C4	10	hsa-mir-6833-3p
Up	SNTG2	1	hsa-mir-146a-5p
Up	TAC4	1	hsa-mir-335-5p
Up	AGMO	23	hsa-mir-1976
Up	PTER	2	hsa-mir-130b-3p

Up	CTRB1	1	hsa-mir-335-5p
Up	COL21A1	2	hsa-mir-29c-3p
Up	ATF7IP2	11	hsa-mir-548c-3p
Up	NEFM	2	hsa-let-7a-5p
Up	S100A14	2	hsa-mir-16-5p
Up	AOX1	1	hsa-mir-26b-5p
Up	KLRF1	1	hsa-mir-335-5p
Up	CCDC68	10	hsa-mir-6808-5p
Up	IRX2	9	hsa-mir-607
Up	CNKSR3	138	hsa-mir-2276-3p
Up	MYZAP	22	hsa-mir-7153-3p
Up	PDE6A	34	hsa-mir-4757-5p
Up	ABCA8	2	hsa-mir-192-5p
Up	INHBA	40	hsa-mir-548i
Up	LDLRAD2	7	hsa-mir-3974
Up	CNTNAP3	4	hsa-mir-1226-3p
Up	C6orf141	22	hsa-mir-1827
Up	RUNX1	60	hsa-mir-1179
Up	TRIM71	104	hsa-mir-3607-3p
Up	PNMT	1	hsa-mir-26b-5p
Up	PRSS50	1	hsa-mir-124-3p
Up	SLC47A1	52	hsa-mir-637
Up	IL20RA	1	hsa-mir-26b-5p
Up	LEFTY2	10	hsa-mir-4267
Up	ITGBL1	18	hsa-mir-579-3p
Up	RHBDL3	4	hsa-mir-8485
Up	GH1	1	hsa-mir-7-5p
Up	FRAS1	1	hsa-mir-124-3p
Up	CT45A1	1	hsa-mir-335-5p
Up	FGFR3	9	hsa-mir-4764-5p
Up	EYA4	9	hsa-mir-203a-3p
Up	AMZ1	19	hsa-mir-1470
Up	CACNA1A	21	hsa-mir-4734
Up	FAM196A	1	hsa-mir-335-5p
Up	CA12	24	hsa-mir-2355-3p
Up	GAS7	18	hsa-mir-384
Up	HPGD	6	hsa-mir-664a-3p
Up	SCGB1D2	1	hsa-mir-26b-5p
Up	PLIN5	17	hsa-mir-1184
Up	ALDOB	2	hsa-mir-378a-3p
Up	GJB7	38	hsa-mir-653-5p
Up	PRR15L	7	hsa-mir-3679-3p
Up	IRX2	9	hsa-mir-6808-3p
Up	ALDH1A3	31	hsa-mir-1304-5p
Up	EYA2	1	hsa-mir-30a-5p
Up	KRT19	2	hsa-mir-193b-3p

Up	LY6H	17	hsa-mir-3188
Up	MYBPHL	19	hsa-mir-4304
Up	RASGRP1	13	hsa-mir-3161
Up	FERMT1	4	hsa-mir-9-5p
Up	ASXL3	28	hsa-mir-1297
Up	PDZRN3	1	hsa-mir-197-3p
Up	KCNS2	19	hsa-mir-888-3p
Up	VANGL2	34	hsa-mir-4733-5p
Up	PRDM16	6	hsa-mir-133a-3p
Up	SH3BP4	42	hsa-mir-663b
Up	COL4A6	2	hsa-mir-197-3p
Up	NEUROD1	17	hsa-mir-6738-3p
Up	IL6R	55	hsa-mir-509-5p
Up	FSHB	7	hsa-mir-3121-5p
Up	CHRDL1	54	hsa-mir-4510
Up	NWD1	75	hsa-mir-548ap-3p
Up	CPXM2	14	hsa-mir-4539
Up	WWC2	39	hsa-mir-596
Up	GJB7	38	hsa-mir-642a-5p
Up	IGSF11	4	hsa-mir-3163
Up	SCN2B	46	hsa-mir-4708-5p
Up	CCDC158	15	hsa-mir-1261
Up	ASXL3	28	hsa-mir-708-3p
Up	DLGAP2	8	hsa-mir-4635
Up	AOX1	1	hsa-mir-26b-5p
Up	CH25H	1	hsa-mir-26b-5p
Up	SLC39A8	6	hsa-mir-331-3p
Up	S100B	2	hsa-mir-29a-3p
Up	SLPI	26	hsa-mir-298
Up	PGR	2	hsa-mir-126-3p
Up	SSTR2	35	hsa-mir-6787-3p
Up	C2orf71	12	hsa-mir-3065-3p
Up	COL13A1	28	hsa-mir-940
Up	CACNA2D3	15	hsa-mir-6503-3p
Up	NR0B1	1	hsa-mir-561-3p
Up	CALB2	1	hsa-mir-335-5p
Up	SCGB1D1	2	hsa-mir-26b-5p
Up	FGF1	24	hsa-mir-589-3p
Up	FOXL2	10	hsa-mir-520a-5p
Up	MKX	31	hsa-mir-548p
Up	TXLNB	27	hsa-mir-642a-5p
Up	ERBB4	7	hsa-mir-302b-3p
Up	PCSK2	25	hsa-mir-660-5p
Up	DMKN	18	hsa-mir-6720-5p
Up	TGFBR3	67	hsa-mir-548aq-3p
Up	MN1	17	hsa-mir-188-3p

Up	SLIT1	33	hsa-mir-629-3p
Up	KCNG1	1	hsa-mir-324-5p
Up	MEGF11	1	hsa-mir-335-5p
Up	PLA2G4A	35	hsa-mir-2114-5p
Up	GPR37L1	15	hsa-mir-4467
Up	SMR3B	1	hsa-mir-495-3p
Up	CLIC3	2	hsa-mir-124-3p
Up	SYT12	2	hsa-mir-146a-5p
Up	CUBN	11	hsa-mir-140-5p
Up	HFM1	11	hsa-mir-4262
Up	PNMA3	12	hsa-mir-6792-3p
Up	IQCA1	1	hsa-mir-193b-3p
Up	ESPNL	1	hsa-mir-375
Up	FAM198A	11	hsa-mir-1321
Up	EFCAB1	12	hsa-mir-1538
Up	FAM124A	9	hsa-mir-6836-3p
Up	ADARB2	30	hsa-mir-3117-5p
Up	DMKN	18	hsa-mir-6720-5p
Up	BANK1	4	hsa-mir-4662a-5p
Up	HIF3A	17	hsa-mir-210-3p
Up	EFCAB1	12	hsa-mir-6769a-3p
Up	CNTNAP3	4	hsa-mir-1226-3p
Up	CCNJL	2	hsa-let-7c-3p
Up	ADAM32	1	hsa-mir-335-5p
Up	LAMA1	1	hsa-mir-124-3p
Up	SEZ6L	10	hsa-mir-3133

Note: Degree: No of miRNA interact with target gene. We taken any one miRNA in table.

Table 8. TF-target gene interaction table.

Regulation	TF	Degree	Gene	Regulation	TF	Degree	Gene
Up	EZH2	73	IRX1	Down	CREM	10	ACER2
Up	WT1	12	ADAMTS18	Down	TRIM28	14	ARHGAP29
Up	ZNF76	22	ADARB2	Down	GATAD1	11	FLJ13224
Up	SUZ12	30	CACNA2D3	Down	SIN3A	28	ANTXR2
Up	EBF1	14	CSHL1	Down	PHF8	31	AKAP3
Up	FOSL2	10	AGR2	Down	MXD4	20	BIRC5
Up	ZNF217	9	AMZ1	Down	SAP30	31	HOXA4
Up	NFIA	11	ALDOB	Down	STAT1	7	CDCA5
Up	ZNF644	17	ATF7IP2	Down	GLI4	8	ASPDH
Up	GATA4	14	CHN2	Down	KDM5B	31	ATP2A3
Up	NR2F2	6	AOX1	Down	KDM5A	35	CCNF
Up	CREB1	14	CNKSR3	Down	ZEB1	26	C3orf52
Up	ZBTB33	25	APOD	Down	ATF1	23	CKS2
Up	TFE3	14	CNKSR3	Down	NR2C2	19	C1orf189
Up	MBD4	10	DPPA4	Down	TFDP1	26	CADPS2

Up	MYBL2	10	HSD17B2	Down	ZNF589	14	DAZAP2
Up	ZFP64	15	C6orf132	Down	POLR2H	14	CDC45
Up	ATF1	18	CCDC68	Down	ETV1	10	FAM83A
Up	GATAD2A	18	CRYM	Down	SMARCA4	12	CENPH
Up	RCOR2	13	FGFR3	Down	MAZ	34	BDNF
Up	BCL6	11	KISS1R	Down	SIRT6	12	HMMR
Up	FOXA3	15	DMKN	Down	REST	24	CDCA5
Up	YY1	9	FABP7	Down	PPARG	17	BIRC5
Up	HMG20B	16	IL6R	Down	ZNF2	21	BUB1
Up	ZNF7	2	PNMT	Down	BCL11A	22	CENPM
Up	NFIC	4	FMO5	Down	LEF1	13	CCNG2
Up	HNF4G	10	PRR15L	Down	INSM2	18	CKS2
Up	MBD1	27	DACT2	Down	BCL3	1	AMIGO2
Up	THAP1	6	FCN2	Down	NCOR1	19	CLTC
Up	RARA	6	DMKN	Down	FOSL2	6	MYBPC1
Up	ETV4	10	ITIH2	Down	ZNF335	16	CYP27B1
Up	L3MBTL2	19	AMOT	Down	TAF7	21	DAZAP2
Up	CTBP1	2	ASXL3	Down	CREB1	15	DHX15
Up	NONO	3	GRHL2	Down	GABPA	16	CLRN2
Up	PPARG	19	CSH1	Down	L3MBTL2	20	C3orf80
Up	MAZ	28	BMP7	Down	ZNF175	10	EXD1
Up	CBFB	7	FFAR3	Down	ZNF639	16	CRNDE
Up	SMAD4	14	CLIC3	Down	KLF16	22	FANCA
Up	RNF2	12	CCNJL	Down	SMAD5	31	CNIH2
Up	ATF4	4	FRAS1	Down	EED	9	KIF18A
Up	HBP1	16	LOC100288911	Down	NRF1	25	FANCA
Up	ATF3	12	C6orf141	Down	SP7	16	B4GALT6
Up	KLF11	14	LINC00242	Down	ZNF580	23	HOXA6
Up	ZNF580	19	ARMC4	Down	SMARCE1	11	KIF20A
Up	GATAD1	15	PHYHD1	Down	POLR2A	20	C20orf203
Up	CTCF	22	ATP4A	Down	DMAP1	14	FAM20C
Up	ZBTB26	16	FAM3B	Down	ZNF501	26	ARHGAP20
Up	ZNF589	11	CA12	Down	SP3	11	G2E3
Up	PKNOX1	11	CSH2	Down	SUZ12	23	CRNDE
Up	ZEB1	11	AQP4	Down	BCL11B	28	DISC1
Up	ZNF263	15	CLIC3	Down	GFI1B	10	FOXM1
Up	KLF1	17	C9orf152	Down	ZNF324	17	C1orf189
Up	ZNF558	7	ARMC3	Down	ARID4B	17	MYO1D
Up	TBX21	5	DMKN	Down	MXD3	21	DOK6
Up	ZNF324	13	WNT5A	Down	SMAD4	16	LOC339874
Up	ZNF547	2	NGFR	Down	IRF4	13	NHLRC4
Up	ZNF202	2	ARMC4	Down	PRDM10	9	MAMSTR
Up	ZNF175	8	ART3	Down	EGR1	13	NXPH4
Up	ZNF239	4	ASXL3	Down	CREB3L1	25	NUSAP1
Up	ZNF121	11	CELSR1	Down	TCF7L2	1	ASPDH
Up	TARDBP	7	MKX	Down	MTA1	15	YTHDF1

Up	SP3	19	FAM196A	Down	SREBF1	3	PARPBP
Up	PRDM2	6	CPLX3	Down	CDC5L	7	LRFN1
Up	BCOR	8	KISS1R	Down	NR2F1	18	ZSCAN12
Up	POLR2H	2	GJB7	Down	TRIM22	15	VWA1
Up	CEBPA	5	SLC6A14	Down	IKZF1	13	TROAP
Up	TRIM24	4	LDLRAD2	Down	ZNF202	6	TRHDE
Up	NR2F1	13	IL22RA1	Down	HIC1	16	TEX22
Up	ZNF384	7	ID4	Down	ID3	18	TCEANC2
Up	NCOR1	6	KAZN	Down	KLF6	12	TTK
Up	NR4A1	11	FOXL2	Down	ZNF76	27	ZRANB2-AS1
Up	GMEB2	2	PRSS8	Down	TCF7	5	SOX4
Up	THRB	11	CTRB1	Down	DRAP1	13	SERINC2
Up	TGIF2	19	NFIX	Down	KLF9	43	UNC13A
Up	NFYC	14	FMO5	Down	HCFC1	15	TRIM24
Up	ZNF512	7	CT45A1	Down	CEBPA	6	RASSF10
Up	GABPA	9	EVC2	Down	MBD1	10	PLK1
Up	ARID4B	13	PPP1R27	Down	MLX	14	ZSCAN2
Up	KDM1A	13	PLIN4	Down	NFYC	11	TPX2
Up	DMAP1	20	C1orf168	Down	KDM1A	9	SPC24
Up	KLF7	21	ENTPD8	Down	ELF1	24	RGS5
Up	GLIS2	27	KCNJ12	Down	MNT	3	PARPBP
Up	ZNF146	6	OVOL3	Down	PBX2	9	TLE4
Up	SP1	14	NTF4	Down	HBP1	15	SLC39A5
Up	ZNF488	2	FXYD3	Down	KLF8	20	RCC1
Up	BCL11A	12	KRT19	Down	KLF11	12	ZC3H12D
Up	ZNF341	19	COL13A1	Down	CHD1	20	RASSF3
Up	BCL11B	16	EYA4	Down	FOXJ2	13	TCEANC2
Up	ZBTB7A	19	SALL1	Down	TEAD3	10	TRIM24
Up	HIC1	23	FGFR2	Down	HMGN3	16	KIF11
Up	ZNF501	17	FAM198A	Down	ZNF584	9	PF4
Up	SOX13	19	MGST1	Down	NCOA1	10	DAZAP2
Up	ESRRA	8	ESRRA	Down	ZKSCAN1	8	PVT1
Up	INSM2	11	PMAIP1	Down	HDAC1	12	HAUS6
Up	ZNF2	17	GAS1	Down	ZNF423	18	KLHL13
Up	ZNF610	11	ROR2	Down	ZFX	23	HMMR
Up	ETV6	1	BMP7	Down	EZH2	34	POU3F4
Up	ZNF394	15	CACNA2D3	Down	EP300	4	PBK
Up	ZNF18	12	RNF207	Down	GTF2E2	28	KIAA1958
Up	ZNF24	15	DNAI2	Down	NFRKB	12	CCNB2
Up	KLF8	11	NKX2-2	Down	HDGF	17	POLQ
Up	ELF3	14	BTBD17	Down	JUNB	6	LINC00309
Up	DIDO1	2	LEFTY2	Down	WRNIP1	24	CALHM1
Up	RERE	11	SOX9	Down	MLLT1	11	PTP4A1
Up	ZNF197	11	WWC2	Down	ZFP37	23	DISC1
Up	MLX	12	FLJ42393	Down	TBX21	8	SPC24
Up	FOSL1	11	FMO5	Down	SMC3	6	BDNF

Up	ESR1	3	LGI1	Down	ZBTB17	8	POU3F3
Up	ZFP2	12	STRA6	Down	EHMT2	1	BDNF
Up	POLR2A	7	LDLRAD2	Down	MYNN	21	C20orf203
Up	NFIL3	11	NEURL3	Down	KLF13	11	INHBE
Up	KLF16	21	GH2	Down	FOSL1	18	PTP4A1
Up	KLF4	16	GADD45G	Down	CTCF	12	PKLR
Up	REST	13	RHBDL3	Down	ZNF644	17	MAMSTR
Up	RAD21	17	RBP4	Down	SSRP1	9	TRIM24
Up	MXD3	14	S100A1	Down	RERE	18	PTTG2
Up	SCRT1	7	GPR37L1	Down	CEBPG	7	MTBP
Up	ZNF335	10	RPRML	Down	ZHX2	11	LOC339874
Up	ELF1	11	DLEC1	Down	ETS1	18	CNIH2
Up	GATA2	7	CA8	Down	SCRT2	6	SLC52A3
Up	KLF9	26	CCKBR	Down	GMEB2	15	ZSCAN12
Up	RXRB	11	TMEM139	Down	MYC	5	FANCA
Up	DPF2	6	GRHL2	Down	SCRT1	3	STMN1
Up	NR2F6	15	PTER	Down	THRB	12	SLC25A15
Up	ZNF71	10	EFEMP1	Down	ZNF197	18	RASSF10
Up	ZNF101	7	RNF207	Down	ZBTB7A	28	QSOX1
Up	JUN	2	C6orf141	Down	MXI1	6	HJURP
Up	ZNF623	5	HIF3A	Down	ZNF394	24	ZNF160
Up	ZNF140	1	C6orf141	Down	ZBTB40	7	G2E3
Up	ZNF292	1	C6orf141	Down	E2F4	14	FANCD2
Up	ZNF264	2	OVOL3	Down	RNF2	9	PNPLA7
Up	CUX1	9	SYT12	Down	ZNF407	8	CCNB2
Up	GFI1B	6	TGFBR3	Down	ZBTB11	21	RAD51
Up	ZNF382	9	KCNG1	Down	MAFG	3	CLTC
Up	PRDM12	1	C6orf141	Down	POU5F1	2	BUB1
Up	FOS	3	IL6R	Down	RCOR2	10	CDKN3
Up	PRDM10	14	STRA6	Down	NR4A1	12	C20orf203
Up	TEAD3	14	TF	Down	TFAP4	16	RFT1
Up	IRF1	10	TEPP	Down	ZNF101	11	CADPS2
Up	SIRT6	5	CA8	Down	ZNF341	11	NXPH4
Up	MIXL1	3	MYBPHL	Down	MEF2D	12	RCC1
Up	ZBTB11	9	SLC39A8	Down	YY1	10	KIF18A
Up	CREB3L1	9	RASL11B	Down	EBF1	8	KIR3DX1
Up	TFDP1	29	VSNL1	Down	TSHZ1	11	PKLR
Up	HMBOX1	5	TMEM154	Down	PKNOX1	4	LYNX1
Up	BHLHE40	7	PPP1R27	Down	WT1	15	RAD21L1
Up	BACH1	3	PNMT	Down	FOXA3	14	FOXM1
Up	FOXJ2	7	S100B	Down	PRDM1	10	PRDM1
Up	SMARCA4	5	FAM83F	Down	ZNF610	16	SLC19A1
Up	SIN3A	17	PAX6	Down	ZNF18	17	DISC1
Up	ZBTB40	6	HPGD	Down	KLF7	20	NHLRC4
Up	ID3	13	SRL	Down	ZBTB33	15	HOXA6
Up	TRIM28	4	FBN2	Down	GLIS2	19	ZBTB33

Up	HDAC1	13	GADD45G	Down	KLF4	8	PRR11
Up	SMARCE1	8	CYBRD1	Down	SUPT5H	7	EVX2
Up	GLI4	4	RUNX1	Down	ARNT	6	CDC45
Up	SCRT2	3	PDE6A	Down	EGR2	13	FLJ13224
Up	GLIS1	15	STRA6	Down	ZNF382	6	HOXA4
Up	EGR2	15	RPRM	Down	RFX1	7	OMP
Up	ATF2	4	CH25H	Down	ELK1	23	ZMAT3
Up	HHEX	5	SOX9	Down	ZNF71	17	NUF2
Up	STAT3	5	CLEC9A	Down	DDX20	16	TSSC2
Up	CEBPG	14	TSPAN8	Down	SP1	25	LOC100268168
Up	HCFC1	4	CLIC3	Down	TEAD2	1	CDC45
Up	TFAP4	14	TRIM71	Down	SMARCA5	13	RAD21L1
Up	NR2C2	10	MFAP4	Down	ZNF83	9	MAMSTR
Up	HMGN3	8	ENTPD8	Down	ZNF24	22	MTBP
Up	FOXM1	7	NTF4	Down	DNMT1	5	KIF20A
Up	EGR1	7	RHOD	Down	GTF2A2	7	FAM83A
Up	IRF4	7	KISS1R	Down	E2F5	13	DHX15
Up	DRAP1	14	NEURL3	Down	IRF2	7	CNIH2
Up	SP7	10	PPP1R27	Down	HMBOX1	5	CLTC
Up	NCOA1	3	TMPRSS3	Down	CREB3	4	MYO1D
Up	CREM	8	S100A14	Down	ZNF623	9	TCEANC2
Up	HMG20A	12	ZNF385C	Down	JUND	8	MYBPC1
Up	LEF1	3	TF	Down	MBD2	9	PRR11
Up	DNMT1	1	CNKSR3	Down	ATF3	12	CRNDE
Up	ZNF423	18	PPP1R1B	Down	MIXL1	6	SLC35G5
Up	TEAD1	9	SPTLC3	Down	ZNF512	7	HTR3D
Up	ADNP	8	RNF207	Down	ATF2	6	NLGN1
Up	HES1	5	GADD45G	Down	ZFP2	23	GPR161
Up	ARNT	2	TEPP	Down	MAFK	5	STMN1
Up	ZFP37	13	RASGRP1	Down	KLF1	21	ZBTB33
Up	ZBTB17	4	KRT19	Down	HDAC8	1	CDC45
Up	MYNN	10	KISS1R	Down	ETS2	2	TRIM24
Up	ARID1B	7	PON3	Down	RELA	3	PKLR
Up	RFXANK	8	PRSS8	Down	NR2F6	22	QSOX1
Up	ZNF143	4	COL13A1	Down	HES1	2	KIF15
Up	SP2	17	EFCAB1	Down	ZHX1	2	TSSC2
Up	CDC5L	5	PTER	Down	TSC22D4	1	CDC45
Up	ZNF16	4	RUNX1	Down	BACH1	6	ZSCAN12
Up	PRDM1	4	TGFBR3	Down	DIDO1	4	CYP27B1
Up	TRIM22	6	SULT1C4	Down	CUX1	7	PVT1
Up	GTF2F1	11	SYT12	Down	ETV6	3	PRR11
Up	TSHZ1	5	KRT19P2	Down	ARID1B	12	RACGAP1
Up	RFX1	5	PGR	Down	ZNF207	15	RAD51D
Up	PTTG1	1	CRISP2	Down	IRF1	24	CENPH
Up	MEF2D	8	PDE6A	Down	GATA4	6	TPX2
Up	SSRP1	15	TRIM71	Down	NFIA	9	VWA1

Up	NRF1	14	SALL1	Down	TGIF2	13	PLK1
Up	KDM5A	2	CRYM	Down	CBFB	9	PSTPIP2
Up	RCOR1	6	GRHL2	Down	TFE3	14	DISC1
Up	HDAC2	5	ESPNL	Down	ZFP64	9	G2E3
Up	HDAC6	5	SSTR2	Down	SIN3B	9	PTP4A1
Up	SMARCA5	11	GH2	Down	GTF2F1	17	CNIH2
Up	E2F5	11	CSH2	Down	ZBTB26	11	SOX4
Up	HDGF	6	CSHL1	Down	CCNT2	8	PNPLA7
Up	ETV1	5	DLEC1	Down	TEAD1	8	NCAPG
Up	PYGO2	1	CYBRD1	Down	GTF2B	8	KIF11
Up	ELK1	6	FOXL2	Down	RFXANK	9	HAUS6
Up	KDM5B	11	WWC2	Down	SOX5	10	CDKN3
Up	NFRKB	7	LEFTY2	Down	NR2F2	4	SPC24
Up	MITF	2	RASGRP1	Down	GATAD2A	11	LYNX1
Up	PHF8	11	ID4	Down	THAP1	4	CLTC
Up	ZNF639	6	MYBPHL	Down	RFX3	6	CENPA
Up	SAP30	11	LOC100288911	Down	ETV4	15	HMMR
Up	CREB3	6	SRL	Down	ZNF384	13	SLITRK6
Up	KLF6	7	VANGL2	Down	SP2	20	UNC13A
Up	ZKSCAN1	5	DLK1	Down	ZNF143	11	RFC3
Up	CBX8	6	SOX2	Down	MTA2	5	FLJ13224
Up	ZNF138	1	DMKN	Down	ZNF121	6	TMEM40
Up	ZNF366	7	WNT5A	Down	BCL6	12	YTHDF1
Up	SUPT5H	5	S100B	Down	ATF4	7	RAD21L1
Up	USF2	5	SYPL2	Down	USF1	3	PNPLA7
Up	POU5F1	5	DPPA4	Down	PML	4	RCC1
Up	EHMT2	3	DRGX	Down	RAD21	16	DOK6
Up	CTBP2	26	LRAT	Down	MYBL2	4	SEL1L
Up	CEBPB	2	PMAIP1	Down	RARA	7	DLEU1
Up	SREBF2	4	MN1	Down	HMG20A	6	EXD1
Up	MTA1	7	GPR37L1	Down	MBD4	4	SEL1L
Up	CCNT2	6	FGFR3	Down	HNF4G	4	CLRN2
Up	JUND	4	KRT19	Down	HMG20B	12	RAD51
Up	KLF13	6	KISS1R	Down	NFATC1	2	PVT1
Up	MBD2	8	LGI1	Down	NONO	3	PRR11
Up	ZFX	8	PAX6	Down	PPARGC1A	1	CLTC
Up	WRNIP1	5	ERP27	Down	JUN	2	PVT1
Up	MLLT1	8	LY6H	Down	HSF1	1	CLTC
Up	ZNF584	4	EVC2	Down	SOX13	9	STMN1
Up	HLF	2	FAM217A	Down	TAL1	5	HJURP
Up	IKZF1	8	PHYHD1	Down	KAT2A	1	CLTC
Up	TEAD4	5	ITIH2	Down	ARID3A	1	CLTC
Up	CEBPD	5	SLC39A8	Down	GATA1	4	RFT1
Up	FOXA2	5	FAM83F	Down	MTA3	2	PVT1
Up	GATA1	5	OR7A5	Down	GATAD2B	3	FAM91A1
Up	ZNF8	2	FXYD3	Down	ESRRA	9	SLC39A5

Up	MAFK	3	TDO2	Down	RXRB	9	SLC25A15
Up	STAT1	2	FFAR3	Down	CTBP2	15	FOXB2
Up	MXD4	17	SH3BP4	Down	ZNF366	10	TROAP
Up	SMC3	4	FLRT2	Down	RUNX3	1	CXCR3
Up	GTF2A2	1	FOXL2	Down	SPI1	1	CXCR3
Up	MNT	1	FRAS1	Down	NFIC	3	ZC3H12D
Up	ZNF641	1	FXYD3	Down	ELF3	7	NXPH4
Up	ZNF354C	1	FXYD3	Down	ZNF558	8	NLGN1
Up	CHD1	12	IRX2	Down	MAX	4	FAM83A
Up	ZNF592	1	GRHL2	Down	ZNF547	6	SV2C
Up	ZNF207	1	GRHL2	Down	PRDM2	10	NUF2
Up	NFATC1	2	S100A14	Down	ZNF263	17	FIBCD1
Up	FOXK2	2	LY6H	Down	ZBTB1	7	PRC1
Up	GATAD2B	4	RHOD	Down	ZNF641	2	LRFN1
Up	MYC	5	SYT12	Down	TARDBP	3	SLC19A1
Up	SIX4	1	GRHL2	Down	NFIL3	9	NUSAP1
Up	MTA3	2	S100A14	Down	HDAC2	6	ZSCAN2
Up	GTF2E2	5	HSD11B1	Down	ESR1	1	EDN1
Up	TBX3	2	PNMT	Down	HDAC6	6	FANCD2
Up	EED	7	NEURL3	Down	ZNF7	4	G2E3
Up	JUNB	5	KAZN	Down	NFYB	2	PRR11
Up	YBX1	2	KCNJ12	Down	GATA2	9	KIF21B
Up	MAFF	3	KCNJ16	Down	PTRF	1	FAM83A
Up	GTF3C2	1	KISS1R	Down	CEBPD	2	FAM83A
Up	ZBTB1	3	TGFBR3	Down	FOXA2	2	TCEANC2
Up	IRF2	1	KISS1R	Down	USF2	6	L1CAM
Up	ZHX2	3	NFIX	Down	SIX4	2	PVT1
Up	CHD4	1	KISS1R	Down	TEAD4	2	FAM83A
Up	SMAD5	5	RNF207	Down	ZNF217	2	RASSF3
Up	MCM3	1	KLK11	Down	RCOR1	3	MTBP
Up	TAL1	3	TEPP	Down	GLIS1	13	PRSS56
Up	RELA	4	PNMT	Down	BCOR	11	ZAN
Up	PTRF	2	LEFTY2	Down	ADNP	12	TMEM40
Up	ARID3A	1	LY6H	Down	BHLHE40	6	TRHDE
Up	NFE2	2	TMEM139	Down	CTBP1	4	POU3F3
Up	ETS1	2	ZNF385C	Down	CEBPB	3	STMN1
Up	AEBP2	1	OVOL3	Down	CBX8	6	ZRANB2-AS1
Up	NFE2L2	2	RHOD	Down	ZNF488	1	HOXA4
Up	DDX20	3	PDE6A	Down	RXRA	1	HOXA4
Up	EP300	1	PI16	Down	ZNF264	1	HOXA6
Up	MTA2	3	RHBDL3	Down	ZNF16	1	HOXA6
Up	KAT2A	1	PMAIP1	Down	ZNF138	1	HOXA6
Up	POU2F2	1	PMAIP1	Down	ZNF707	1	INHBE
Up	TCF7	1	PMAIP1	Down	NFE2L1	1	KIAA1958
Up	PML	2	S100A1	Down	CHD4	1	KIF11
Up	NR3C1	1	PNMT	Down	TBP	4	NCAPG

Up	MAX	2	RNF207	Down	MAFF	2	KIF18A
Up	ZNF707	1	PPP1R27	Down	GATA3	5	SPEF2
Up	MXI1	2	PRSS8	Down	HLF	2	RAD21L1
Up	NFYB	1	PRR15L	Down	THRAP3	3	RCC1
Up	GATA3	1	PRR15L	Down	ZNF8	1	LRFN1
Up	DEK	2	RHOD	Down	ZNF239	2	ZNF160
Up	E2F6	1	RNF207	Down	SIX5	1	MAMSTR
Up	TAF7	2	S100A1	Down	RBBP5	2	MTBP
Up	HDAC8	1	RUNX1	Down	DPF2	5	PRC1
Up	ZNF83	1	RUNX1	Down	MITF	2	ZBTB33
Up	RUNX3	1	RUNX1	Down	SREBF2	5	PNPLA7
Up	SPI1	1	RUNX1	Down	ZNF585B	1	OXR1
Up	SREBF1	1	RUNX1	Down	NFE2	5	PF4
Up	GTF2B	1	S100A1	Down	MCM7	1	PKLR
Up	PBX2	1	SALL1	Down	FOXK2	1	PRR11
Up	ZNF407	1	SYPL2	Down	ZNF592	1	PVT1
Up	HNF4A	1	TF	Down	ZNF140	1	SLC5A10
Up	RXRA	2	TPPP2	Down	ZNF146	2	TROAP
Up	TEAD2	1	WWC2	Down	TBX3	2	ZC3H12D
				Down	HHEX	1	TPX2
				Down	CEBPZ	1	TSSC2
				Down	SP4	1	ZSCAN12

Note: Degree: No of target gene interact with TF. We taken any one target gene in table. TF transcription factors.
